# Enantioselective Synthesis of Triarylmethanes via Intermolecular C–H Functionalization of Cyclohexadienes with Diaryldiazomethanes

**DOI:** 10.1021/acs.orglett.3c00845

**Published:** 2023-05-30

**Authors:** Maizie Lee, Huw M. L. Davies

**Affiliations:** Department of Chemistry, Emory University, 1515 Dickey Drive, Atlanta, Georgia 30322, United States

## Abstract

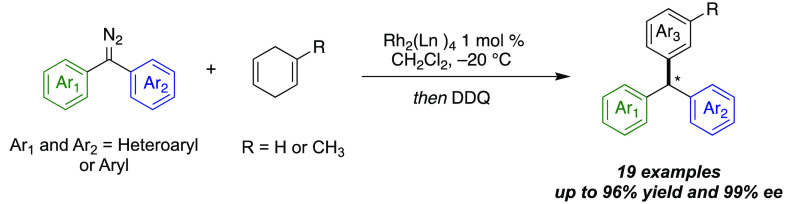

Rhodium-catalyzed C–H functionalization of cyclohexadiene derivatives with diaryldiazomethanes followed by oxidation with DDQ provides ready access to triarylmethanes. Two chiral dirhodium tetracarboxylates, Rh_2_(*S*-PTAD)_4_ and Rh_2_(*S*-TPPTTL)_4_, were found to be the optimum chiral catalysts for these transformations. This method showcases the ability of diaryldiazomethanes to perform intermolecular C–H insertion with high enantioselectivity and good yields. The method has a broad substrate scope, leading to triarylmethane products with a variety of aryl and heteroaryl substituents, including benzofuran and pyridine heterocycles.

The metal-catalyzed reactions of transient metal carbenes have broad utility in organic synthesis.^1^ The structure of the carbene has a dramatic impact on the outcome of the reaction (Scheme 1a).^2^ Most of the early chemistry was conducted on acceptor and acceptor/acceptor carbenes,^3^ but since then, donor/acceptor carbenes have become prominent^4^ because the donor group attenuates the reactivity of the highly electrophilic carbene, enabling numerous highly selective reactions to be viable. More recent studies have explored whether donor/donor carbenes could be another class of highly selective carbenes.^5^ Much of the earlier work focused on intramolecular reactions, presumably because the carbenes are less reactive than the other classes of carbenes.^5b,6^ Very recently, we^5a^ and others^7^ have shown that diarylcarbenes generated from diaryldiazomethanes are capable of enantioselective reactions, such as cyclopropanation, Si–H insertion, and B–H insertion (Scheme 1b).

**Scheme 1:**
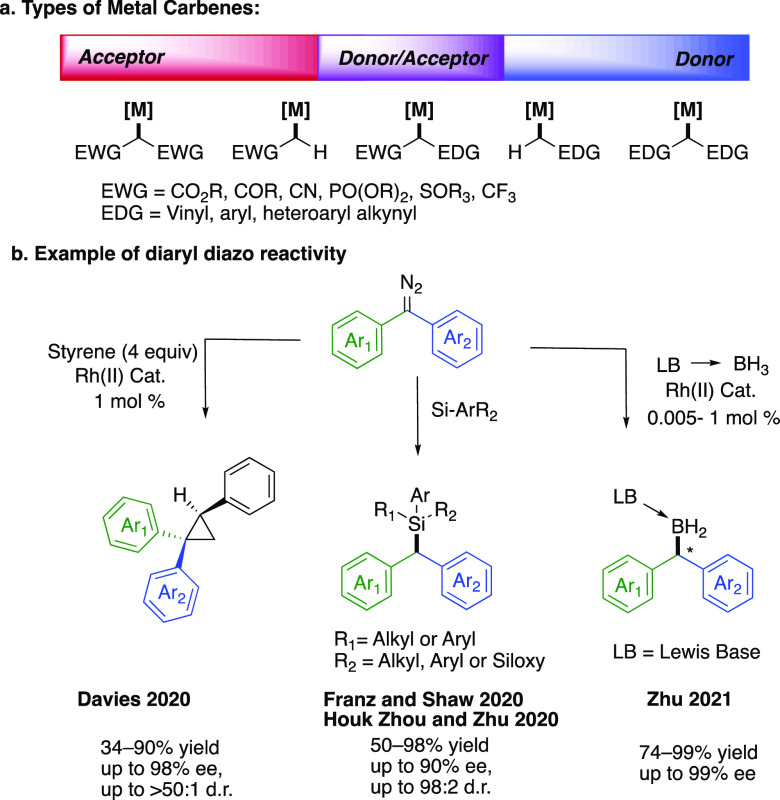
(a) Classes of Carbene Intermediates and (b) Enantioselective Intermolecular Reactions with Diaryldiazomethanes

The diarylcarbenes have been formally considered as donor/donor carbenes,^2c^ but we have proposed it is more appropriate to consider them as donor/acceptor carbenes^4a^ because the two rings cannot be simultaneously in the same plane as the rhodium carbene.^5a^ When a ring is in the same plane as the carbene, it would behave as a donor group, but due to steric constraints, the other ring would need to be orthogonal to the carbene and then would behave as an acceptor group. This behavior is illustrated in Scheme 2, which includes the calculated values for the tilting angles for the two aryl groups in a representative diarylcarbene. Two diarylcarbene systems that strongly exhibit this behavior are those with a strong *para* donor on one ring and those with an *ortho* substituent on one ring. We have demonstrated this concept in our experimental and computational studies on rhodium-catalyzed cyclopropanation with diaryldiazomethanes.^5a^ In this study, we demonstrate the enantioselective reactions can be extended to C–H functionalization of cyclohexadienes,^8^ leading to the enantioselective synthesis of triarylmethanes. Due to their pharmaceutical relevance, methods for their enantioselective synthesis have been extensively explored, but it is still challenging to achieve broad scope.^9^ Our method extends the range of triarylmethanes that can be readily formed with high enantioselectivity.

**Scheme 2:**
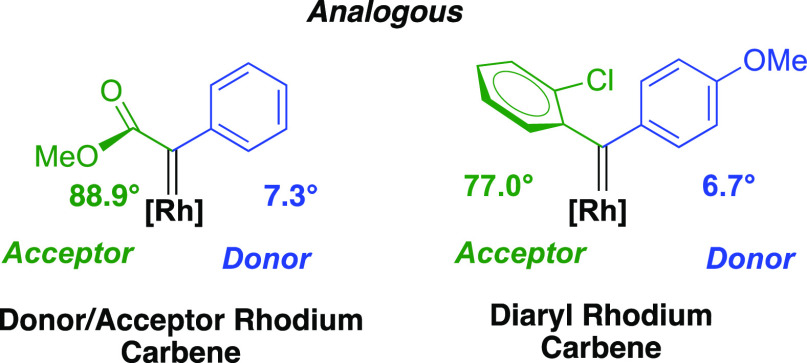
Diarylcarbenes as Donor/Acceptor Carbenes and Our Work

The study began via evaluation of the C–H insertion reaction of 1,4-cyclohexadiene (**2**) with 4-nitro-4′-methoxydiphenyldiazomethane (**1**), a precursor to a prototypical diaryl carbene in which one aryl group has an electron-withdrawing group and the other has an electron-donating group. A series of chiral dirhodium catalysts were examined, and all of the catalysts generated the desired product **3** as summarized in Table 1. As expected, Rh_2_(*S*-DOSP)_4_ gave a very low level of asymmetric induction because it requires an ester group as an acceptor group for a high degree of asymmetric induction.^10^ The triaryl cyclopropane carboxylate catalyst Rh_2_(*S*-2-Cl-5-BrTPCP)_4_^11^ significantly boosted the asymmetric induction of the C–H insertion, with an ee of 79%, but the best class of catalysts was the naphthylimido- and phthalimido-derived catalysts.^12^ The phthalimido-derived catalysts have interesting properties because they self-assemble into bowl-shaped structures that can be relatively rigid.^13^ The naphthylimido catalyst Rh_2_(*S*-NTTL)_4_^14^ gave excellent results, generating **3** in 94% ee, but some of the phthalimido catalysts performed even better, such as Rh_2_(*S*-PTAD)_4_,^15^ which generated **3** in 77% yield and 99% ee.

**Table 1:**
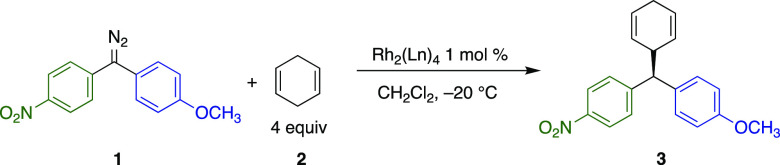
Chiral Catalyst Screening^a^

entry	catalyst	yield (%)	ee (%)
1	Rh_2_(*S*-DOSP)_4_	64	3
2	Rh_2_(*S*-2-Cl 5-BrTPCP)_4_	60	−79
3	Rh_2_(*S*-NTTL)_4_	73	94
4	Rh_2_(*S*-TPPTTL)_4_	85	79
5	Rh_2_(*S*-PTTL)_4_	80	98
6	Rh_2_(*S*-TCPTAD)_4_	80	97
**7**	**Rh_2_(*S*-PTAD)_4_**	**77**	**99**

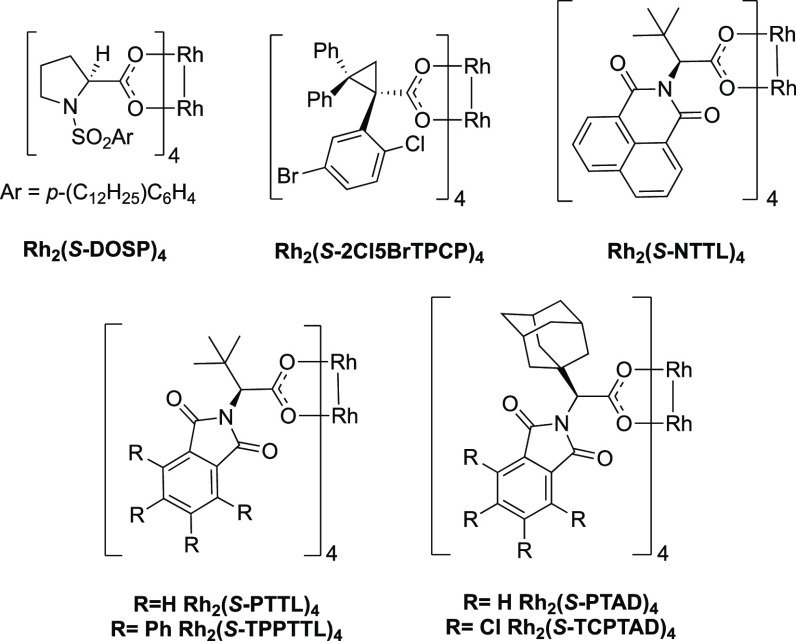

a For the reaction, **1** (0.20 mmol) in a solution of CH_2_Cl_2_ was inversely added to a solution of **2** (4 equiv) and Rh_2_(Ln)_4_ (1 mol %) in CH_2_Cl_2_ (1 mL) over 1 h at −20 °C under N_2_.

The oxidation of 1,3-cyclohexadienes to benzene derivatives is well-established.^16^ Therefore, the C–H functionalization described above was expected to be a convenient asymmetric method for the construction of triarylmethanes. Confirmation that this was indeed feasible was demonstrated by conversion of **3** to triarylmethane **4** using 2,3-dichloro-5,6-dicyano-1,4-benzoquinone (DDQ) as the oxidant (Scheme 3). Most importantly, the oxidation was achieved with no racemization.

**Scheme 3:**
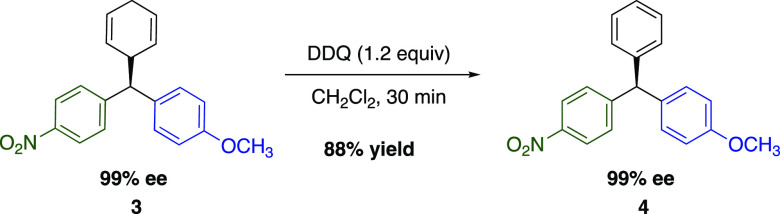
Stereoretentive Oxidation to Triarylmethane **4**

With the optimized conditions set forth, the rhodium-catalyzed reaction with cyclohexadiene combined with DDQ oxidation was applied to a series of diaryldiazomethanes **5**–**9** lacking *ortho* substituents (Scheme 4). The combined reactions were compatible with a variety of substituents, but substrates containing a strong electron-withdrawing group such as nitro or trifluoromethyl gave the highest levels of asymmetric induction, as seen with **5** and **6**. The reactions with a *p*-CF_3_ substituent gave triarylmethane **6** in 80% yield and 85% ee, whereas the reaction with a *p*-chloro substrate generated **7** in 61% and 79% ee. Electron rich or electron deficient heterocyclic substrates can be incorporated into the diazo compound as illustrated in the formation of **8** and **9**, although the enantioselectivity was lower in these cases (45% and 77% ee, respectively). Typical reactions were conducted on a 0.3 mmol scale, but the reaction is easily scalable, as illustrated in the formation of **4** on a 1 mmol scale in 90% yield and 99% ee.

**Scheme 4:**
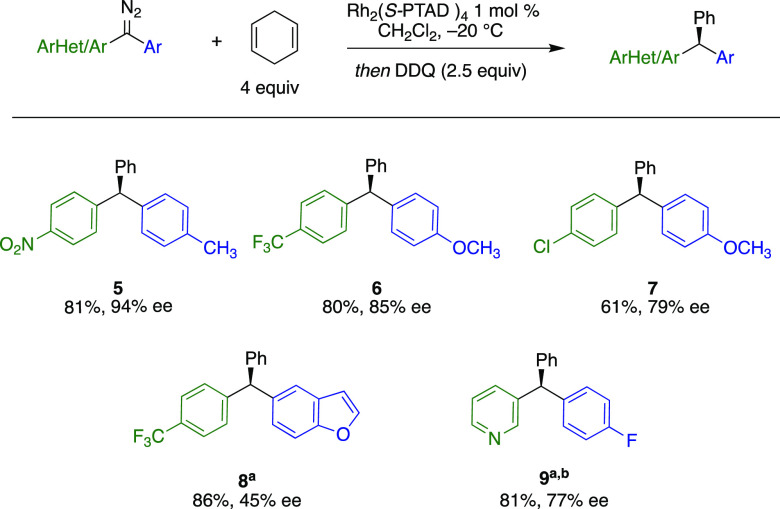
Substrate Scope of Triarylmethane Compounds Triarylmethane compound could not be resolved using chiral HPLC. The ee value is estimated from the analysis of the C–H insertion intermediate. With 20 equiv of HFIP added to the mixture. For the reaction, 0.30 mmol of diazo was inversely added to a solution of Rh_2_(*S*-PTAD)_4_ and 4 equiv of the cyclohexadiene substrate in 1 mL of CHCl_3_.

To further extend the scope of these reactions, the reaction with 1-methyl-1,4-cyclohexadiene was examined (Scheme 5). The C–H functionalization reaction of diaryldiazomethane **10** produces a mixture of diastereomers **11a** and **11b** in a 3:1 ratio. On prolonged standing, the major diastereomer crystallized from the mixture, and the relative and absolute configurations of **11a** could be determined by X-ray crystallography. Major diastereomer **11a** was produced with a higher level of asymmetric induction (86% ee) compared to that of minor diastereomer **11b** (52% ee). The two diastereomers could barely be separated by chromatography. Hence, the combined mixture was oxidized by DDQ to form triarylmethane **12** in 77% ee. This value matches the expected calculated value assuming that both **11a** and **11b** are generated with the same sense of asymmetric induction and that the oxidation of **11a** and **11b** to **12** occurs without a loss of the asymmetric induction. The absolute configuration of **12** is assigned as *R* because it is derived from **11a** of known absolute configuration. The absolute configurations of all of the other triarylmethanes are tentatively assigned by analogy to **12**.

**Scheme 5:**
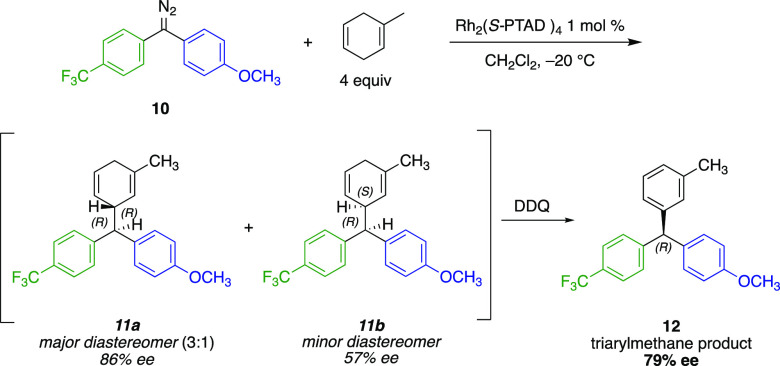
C–H Insertion of 1-Methyl-1,4-cyclohexadiene

The C–H functionalization of 1-methyl-1,4-cyclohexadiene could be applied to a variety of diaryldiazomethanes as illustrated in Scheme 6. A diastereomeric mixture of the C–H insertion products was generated, and these were directly oxidized with DDQ to form the desired triarylmethanes **13**–**15** in 51–89% yields and 75–93% ee. Once again, the highest enantioselectivity was obtained using the 4-nitro-4′-methoxydiaryldiazomethane in which the electronic differentiation between the donor and the acceptor group is most pronounced. In this case, **15** was formed in 93% ee. A catalyst screen of the reaction to afford **15** revealed that Rh_2_(*S*-PTAD)_4_ remained the optimal catalyst (see the Supporting Information and Table 1).

**Scheme 6:**
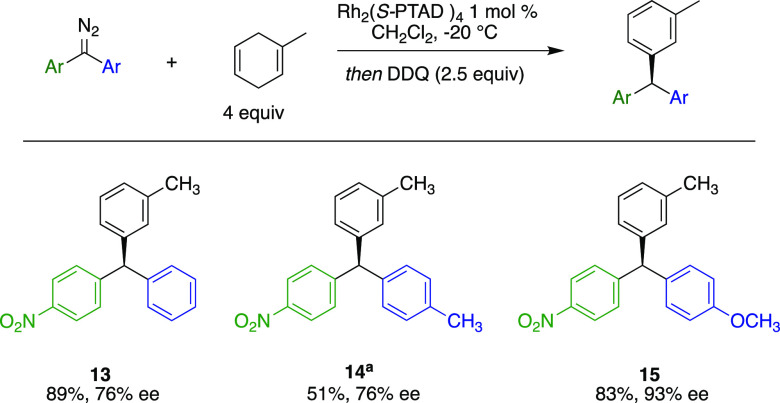
C–H Functionalization of 1-Methyl-1,4-cyclohexadiene The ee value is an estimated value due to the imperfect resolution of peaks in the HPLC spectrum. For the reaction, 0.30 mmol of diazo was inversely added to a solution of Rh_2_(*S*-PTAD)_4_ and 4 equiv of the cyclohexadiene substrate in 1 mL of CHCl_3_.

In our previous studies,^5a^ addition of an *o*-chloro substituent was found to enhance diastereoselectivity in the case of cyclopropanation of styrene. We employed a catalyst screen on 2-chloro-4-nitrodiphenyldiazomethane to verify if enhanced enantioselectivity of the final triarylmethane product could be obtained using some of the other bowl-shaped catalysts (Table 2).^17^ While Rh_2_(*S*-PTAD)_4_ was found to give the desired product in 74% ee (entry 1), Rh_2_(*S*-TPPTTL)_4_^18^ gave a significant increase to 88% ee. Rh_2_(*S*-TPPTTL) has been previously shown to give superior asymmetric induction in cyclopropanation reactions with *ortho*-substituted aryldiazoacetates,^19^ and similar characteristics are observed here in the reaction with *ortho*-substituted diaryldiazomethanes.

**Table 2:**
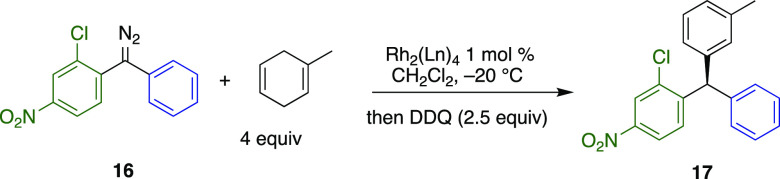
Catalyst Screen of C–H Insertion of 1-Methyl-1,4-cyclohexadiene^a^

entry	catalyst	yield (%)	ee (%)
1	Rh_2_(*S*-PTAD)_4_	94	74
2	Rh_2_(*S*-TCPTAD)_4_	87	6
3	Rh_2_(*S*-NTTL)_4_	78	–6
4	Rh_2_(*S*-PTTL)_4_	92	51
5	Rh_2_(*S*-TPPTTL)_4_	94	88

a For the reaction, 0.20 mmol of diazo **16** was inversely added to a solution of Rh_2_(*S*-PTAD)_4_ and 4 equiv of the cyclohexadiene substrate in 1 mL of CHCl_3_.

In Scheme 7, diazo compounds containing an *o*-chloro substituent were employed for the C–H functionalization of a variety of substituted cyclohexadiene compounds, which could then be oxidized to the triarylmethane. Triarylmethane **18**, derived from a diaryldiazomethane bearing an electron-donating methoxy group- was produced in 82% yield and 87% ee. Triarylmethane **19**, derived from a diaryldiazomethane lacking the methoxy group, was generated with a slightly lower enantioselectivity (83% ee). Triarylmethanes **20** and **22**, derived from diaryldiazomethanes having an electron-donating group, an electron-withdrawing group, and an *o*-chloro group, were generated with the highest levels of enantioselectivity (98% and 91% ee, respectively). These trends are consistent with our understanding that diaryl diazo compounds that closely model that of a donor/acceptor diazo compound through electronic and steric properties give the higher levels of asymmetric induction. The reaction could be also extended to various substituted cyclohexadienes, resulting in the formation of **23**–**25**. In addition to the 1-methyl-substituted cyclohexadiene, three other substituted 1,4-cyclohexadiene derivatives were shown to produce triarylmethane products. The formation of **25** is an interesting transformation because the C–H functionalization was site selective, favoring by a 12:1 ratio insertion α to the methyl group versus insertion α to the isopropyl group in γ-terpene.

**Scheme 7:**
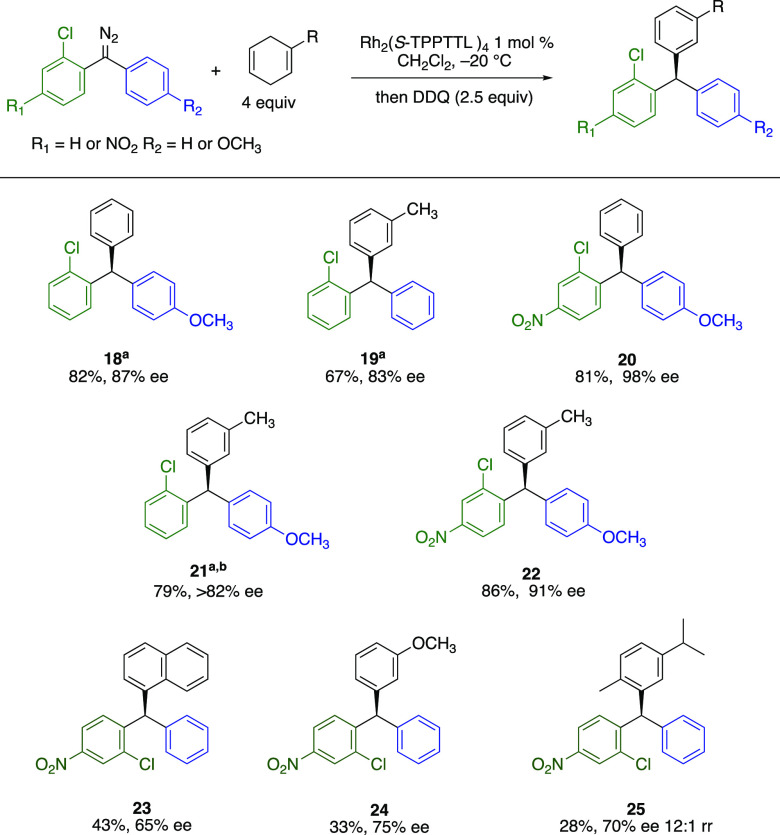
C–H Functionalization of Cyclohexadienes with *o*-Cl Diaryldiazomethanes The triarylmethane compound could not be resolved using chiral HPLC. The ee value is estimated from the analysis of the C–H insertion intermediate. The ee value was assigned on the basis of the analysis shown in Scheme 5 and the crystal structure of **11a**. The ee value is an estimated value due to the imperfect resolution of peaks in the HPLC spectrum. For the reaction, 0.30 mmol of diazo was inversely added to a solution of Rh_2_(*S*-PTAD)_4_ and 4 equiv of the cyclohexadiene substrate in 1 mL of CHCl_3_.

In conclusion, we have developed a facile enantioselective synthesis of triarylmethanes using rhodium-catalyzed C–H functionalization of cyclohexadienes with diaryldiazomethanes. Our system allows a variety of diaryl precursors to be incorporated into new triarylmethane scaffolds with high enantioselectivity, a previously difficult task with current literature methods. This method can tolerate a variety of electron rich and poor aryl substituents and bulky *o*-chloro substituents and is compatible with two heterocycles. Furthermore, the diaryl system, previously used for mainly intermolecular reactions, now has been shown to have the ability to perform C–H functionalization on activated systems, which broadens their synthetic potential.

## Data Availability

The data underlying this study are available in the published article and its online Supporting Information.

## Supporting Information Available

Complete experimental procedures and compound characterization (PDF)
