# Supplementary material for: Enantioselective Synthesis of Triarylmethanes via Intermolecular C–H Functionalization of Cyclohexadienes with Diaryldiazomethanes
Source: Org Lett. 2023 May 30;25(22):4000–4. doi: 10.1021/acs.orglett.3c00845 (PMC10262276; doi:10.1021/acs.orglett.3c00845)
Supplement: Supplementary file 1 — ol3c00845_si_001.pdf [file ol3c00845_si_001.pdf]

Supporting Information

Enantioselective Synthesis of Triarylmethanes *via* Intermolecular C-H Functionalization of Cyclohexadienes with Diaryldiazomethanes

Maizie Lee and Huw M. L. Davies*

Department of Chemistry, Emory University, 1515 Dickey Drive, Atlanta, Georgia 30322.

Email: hmdavie@emory.edu

Table of Contents

1. General Considerations **S-2**

2. Catalysts and Diazo Compounds **S-3**

3. SI Table 1. Catalyst Screen **S-4**

4. Experimental procedures **S-5**

5. ^{1}H ^{13}C and ^{19}F NMR Spectra for Characterization of Compounds. **S-18**

6. X-Ray Crystallographic Data for Compound 11a. **S-46**

7. References **S-55**

8. HPLC Spectra for Enantioselectivity Determination. **S-56**

1. General Considerations

Caution: Diazo compounds are high energy compounds and should be handled with caution. Although we have had no difficulties with working with these compounds, it is advisable to carry out reactions on small scale behind a blast shield. Hydrazine hydrate is highly toxic compound and needs to be handled using the established safety protocols. In addition, Diaryl Diazo compounds are known to be unstable and must be handled with caution and stored in a -20 °C freezer to avoid decomposition over time.

Substrates and reagents were purchased from the following suppliers and used without further purification: Sigma-Aldrich, Alfa-Aesar, Oakwood Chemical America, and Fisher Scientific. All solvents were purified and dried by a Glass Contour Solvent System, and stored over 4 Å molecular sieves 24 hours before use. All reactions were carried out in flamed-dried glassware unless otherwise stated. ^{1}H NMR spectra were recorded at 600 MHz on Bruker-600 spectrometer, Varian INOVA 500 MHz or Varian 400 MHz. ^{13}C NMR spectra were recorded at 150 MHz on Bruker-600. NMR spectra samples were prepared using deuterated chloroform($CDCl_3$) with residual solvent serving as internal standard 7.26 ppm for ^{1}H and 77.16 ppm for ^{13}C; or with Deuterated chloroform 0.03% TMS with residual TMS serving at internal standard (0.00 ppm). Abbreviations for signal multiplicity are as follows: s= singlet, d= doublet, t= triplet, m= multiplet, dd= doublet of doublets dt= doublet of triplets. The coupling constants J, are reported in Hertz and integration is provided, along with assignments, as indicated. Structural assignments were made with additional information from gCOSY, gHSQC, and gHMBC experiments. Optical rotations were measured on Autopol IV automatic polarimeter by Rudolph Research Analytical. Crystallographic data was obtaining through the Emory X-ray Crystallography center using the ShelXT 2018 solution program. IR spectra were collected on a Nicolet Is10 FT-IR spectrometer. Mass spectra were taken on a Thermo Finnigan LTQ-FTMS spectrometer with APCI ESI or NSI. Thin layer chromatographic analysis (TLC) was performed on aluminum-sheet silica gel plates, and visualized with UV light. Racemic standards for enantiomeric determination were generated with reactions with $Rh_2(OAc)_4$ or from Rh_2((*R*) and (*S*)-DOSP$)_4$ which was generated by dissolving equimolar mixture of R and S catalyst in a minimal amount of benzene and lyophilizing. High performance liquid chromatography analysis (HPLC) was performed on Agilent 1100 Technologies HPLC instrument.

2. Catalysts and Diazo Compounds

Catalyst Structures:

The following catalysts were used in this study and have been previously prepared.

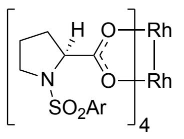

Ar = *p*-$(C_{12}H_{25})C_6H_4$

$Rh_2(S\text{-DOSP})_4$
MW = 1896.23
ref:1a

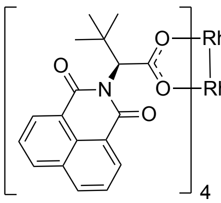

$Rh_2(S\text{-NTTL})_4$
MW = 1447.13
ref:1b

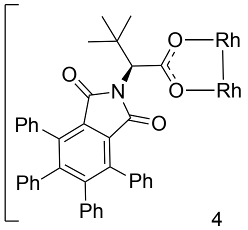

$Rh_2(S\text{-TPPTTL})_4$
MW = 2464.46
ref:1c

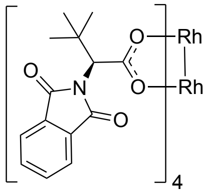

$Rh_2(S\text{-PTTL})_4$
MW = 1246.89
ref:1d

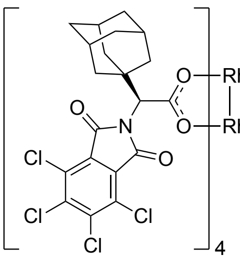

$Rh_2(S\text{-TCPTAD})_4$
MW = 2110.41
ref:1e

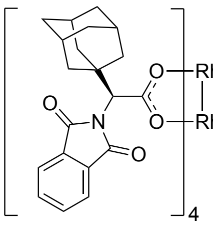

$Rh_2(S\text{-PTAD})_4$
MW = 1559.34
ref:1f

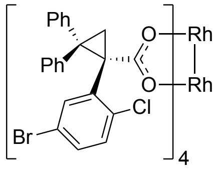

$Rh_2(S\text{-2Cl5BrTPCP})_4$
MW = 1912.67
ref:1g

Diazo Compounds

The following Diazo compounds were previously reported.

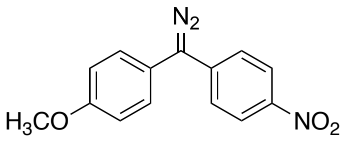

ref: 2a

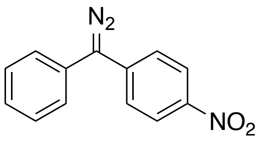

ref: 2a

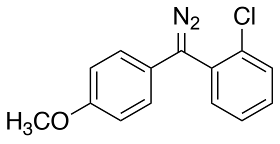

ref: 2a

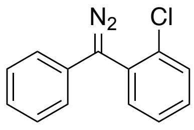

ref: 2a

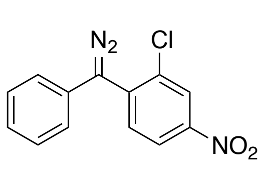

ref: 2a

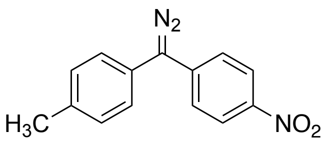

ref: 2a

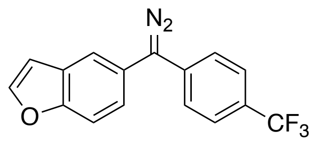

ref: 2b

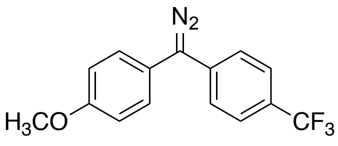

ref: 2b

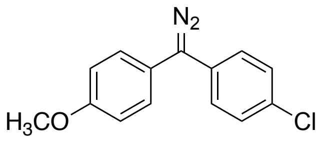

ref: 2b

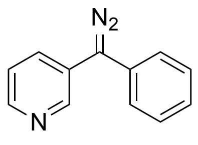

ref: 2c

3. SI Table 1. Catalyst Screen

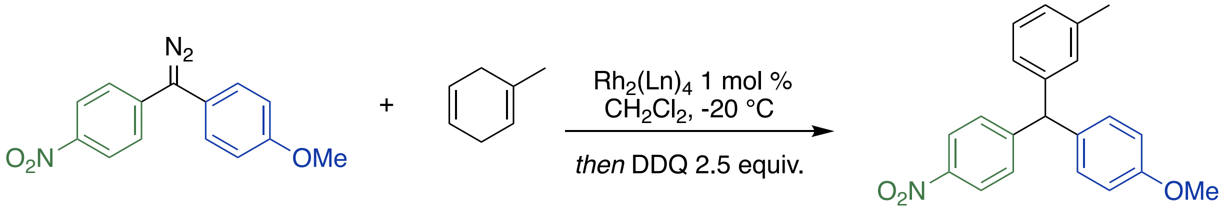

Entry	Catalyst	e.e.(%)
1	$Rh_2(S\text{-}PTAD)_4$	93
2	$Rh_2(S\text{-}NTTL)_4$	91
3	$Rh_2(S\text{-}TPPTTL)_4$	77
4	$Rh_2(S\text{-}PTTL)_4$	84

4. Experimental procedures

4a.) Preparation of diaryl/heteroaryl diazo compound

Procedure adopted from literature.[3] A 50 mL dried round bottom flask was charged with desired hydrazone (1.1 mmol) and THF (4 mL) followed by tsNIK(1.2 mmol) prepared by following the literature procedure.[4] Potassium hydroxide in 1 M solution (1 mL) was slowly added to the reaction flask. The reaction was monitored by TLC, with disappearance of all starting hydrazone derivatives by 1.5 h. The reaction was poured into 5 mL potassium hydroxide in 1 M solution and extracted with diethyl ether (2 x 30 mL). The organic layers were combined then washed with brine (2 x 30 mL) and dried over $MgSO_4$. After removal of the solvent, the desired diazo compound was obtained. If necessary, the diazo compound was purified using an alumina column with a solvent gradient of 0 to 5 % diethyl ether in hexanes. The product was stored under argon at –20 °C.

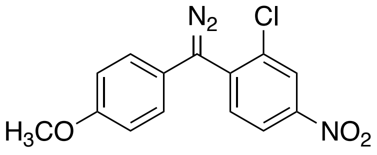

2-chloro-1-(diazo(4-methoxyphenyl)methyl)-4-nitrobenzene (SI 1)

The diazo compound was prepared from (*E*)-((2-chloro-4-nitrophenyl)(4-methoxyphenyl)-methylene)hydrazone and was obtained as a red solid on a 2.6 mmol scale in 71% yield (550 mg).
^{1}H NMR (600 MHz, $CDCl_3$) δ 8.33 (d, *J* = 2.4 Hz, 1H), 8.06 (dd, *J* = 8.8, 2.4 Hz, 1H), 7.39 (d, *J* = 8.8 Hz, 1H), 7.14 (d, *J* = 8.8 Hz, 2H), 6.99 (d, *J* = 8.8 Hz, 2H), 3.86 (s, 3H).
^{13}C NMR (151 MHz, $CDCl_3$) δ 158.7, 145.8, 136.7, 131.8, 128.6, 127.0, 126.5, 122.0, 120.3, 115.2, 55.5 (The resonance resulting from the diazo carbon was not observed).
IR: 3090, 3007, 2963, 2044, 1506, 1473, 1302, 1274, 1246, 1184, 1119, 875, 841, 739 cm^{-1}.
HRMS (+p APCl) *m/z*: calcd for $C_{14}H_{10}ClN_3O_3$ [M+H-N_2] 276.0427; found 276.0426.

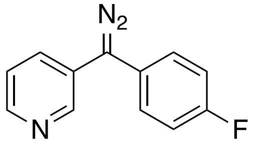

3-(diazo(4-fluorophenyl)methyl)pyridine (SI 2)

The diazo compound was prepared from (*Z*)-3-((4-fluorophenyl)(hydrazineylidene)methyl)pyridine and was obtained as a red/purple solid at a 3.70 mmol scale in 69% yield (548 mg).
^{1}H NMR (400 MHz, $CDCl_3$) δ 8.49 (dd, *J* = 4.9, 1.7 Hz, 2H), 7.45 – 7.36 (m, 2H), 7.25 – 7.16 (m, 2H), 7.02 (dd, *J* = 6.3, 1.7 Hz, 2H).
C NMR: Compound was not stable enough in $CDCl_3$ to obtain ^{13}C.
^{19}F NMR (376 MHz, $CDCl_3$) δ -113.3.
IR: 3036, 2042, 1585, 1507, 1493, 1446, 1220, 1159, 988, 942 cm^{-1}
HRMS ($^+$APCl) *m/z*: calcd for $C_{12}H_9ONF$ [M+OH-N_2] 202.0663; found 202.0666.

4b.) General procedure a.) C-H insertion reaction of Cyclohexadienes:

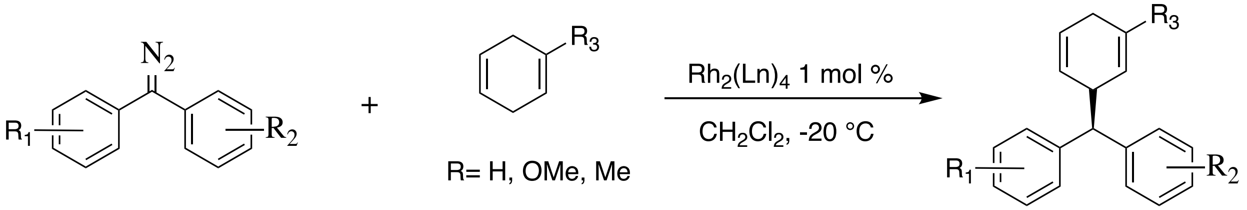

To a flame-dried 12 mL dram vial with a stir bar the desired dirhodium catalyst (1 mol %) was added, and the reaction vial was purged with nitrogen three times and then the catalyst was dissolved in CH_2Cl_2 (1.0 mL). The vial was placed in a salt/ice bath to reach –20 °C and was charged with substituted Cyclohexadiene (4 equiv). The corresponding diazo compound (0.30 mmol) was weighed in a 20 mL vial and dissolved in dry degassed DCM (5.0 mL) under N_2. The diazo compound solution was then added to the reaction vial dropwise over 1 h at (–20 °C) *via* a syringe pump. The reaction was stopped after 14 h. The product was purified *via* flash column chromatography with a gradient of 0 to 15 % diethyl ether in hexanes.

4c.) General procedure b.) one-pot C-H insertion followed by oxidation to TRAM:

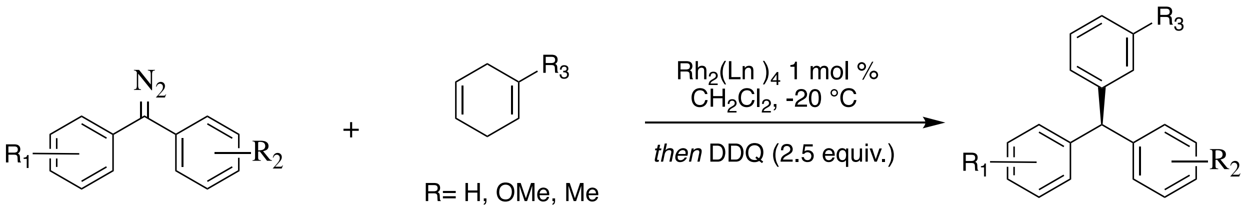

To a flame-dried 16 mL dram vial the desired dirhodium catalyst (1 mol %) was added, and the reaction vial was purged with nitrogen three times and dissolved in CH_2Cl_2 (1.0 mL). The vial was placed in a salt/ice bath to reach –20 °C and was charged with substituted cyclohexadiene (4 equiv) The corresponding diazo compound (0.30 mmol) was weighed in a 20 mL vial and dissolved in dry degassed DCM (5.0 mL) under N_2. The diazo compound in DCM solution was then added to the reaction vial dropwise over 1 h at (–20 °C) *via* a syringe pump. In some cases, a small aliquot was kept for HPLC analysis of C-H insertion intermediate. After 14 h, 2,3-dichloro-5,6-dicyano-1,4-benzoquinone (DDQ) (2.5 equiv) was added into the mixture and allowed to stir for 2 h. The reaction was stopped, and filtered through a celite plug to remove excess DDQ. The mixture was purified *via* flash column chromatography with a gradient of 0 to 10% diethyl ether in hexanes to afford the desired triarylmethane compounds, as white solids (racemic compounds) or an oil (enantioenriched material).

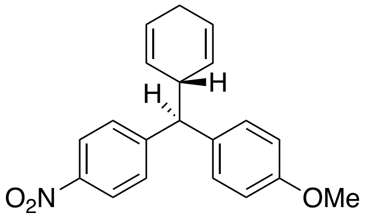

(*R*)-1-(cyclohexa-2,5-dien-1-yl(4-methoxyphenyl)methyl)-4-nitrobenzene (3)

Compound 3 was obtained according to general procedure C from the C–H functionalization reaction between 1,4-cyclohexadiene (0.1 mL, 0.2 mmol, 4 equiv) and 1-(diazo(4-methoxyphenyl)methyl)-4-nitrobenzene (54 mg, 0.2 mmol, 1.0 equiv), catalyzed by Rh2(*S*-PTAD)4 (3.12 mg, 0.002 mmol, 1.0 mol %). The product was purified by flash column chromatography on silica gel (gradient elution: 0

– 2% diethyl ether in pentane) to afford a white solid in 77% yield (49 mg).

^{1}H NMR (400 MHz, $CDCl_3$) δ 8.14 (d, *J* = 8.7 Hz, 3H), 7.45 (d, *J* = 8.8 Hz, 3H), 7.19 (d, *J* = 8.7 Hz, 3H), 6.85 (d, *J* = 8.7 Hz, 3H), 5.73 (dtd, *J* = 10.4, 3.3, 1.7 Hz, 4H), 5.53 (ddd, *J* = 10.8, 3.6, 2.0 Hz, 1H), 5.45 (ddd, *J* = 10.8, 3.7, 2.1 Hz, 1H), 3.91 (d, *J* = 9.7 Hz, 2H), 3.61 (ddt, *J* = 6.4, 3.3, 1.6 Hz, 1H), 2.63 (dtd, *J* = 8.3, 3.4, 1.7 Hz, 4H).
^{13}C NMR (101 MHz, $CDCl_3$) δ 158.4, 151.3, 146.4, 133.5, 129.2, 127.0, 126.7, 126.1, 125.8, 123.7, 114.2, 57.4, 55.3, 39.1, 26.5.
IR(in $CDCl_3$): 3030, 2836, 1605,1509,1345, 1253,1179, 1034, 806, 695 cm^{-1}.
HRMS ($^-$APCI) *m/z*: calcd for $C_{20}H_{18}NO_3^-$ $[M]^-$ 320.1292; found 320.1285.
$[\alpha]^{22}_D$ –27.5 (*c* 1.18, $CHCl_3$)
HPLC conditions: HPLC (ADH column, 1.0 mL/min 1% *i*-PrOH in *n*-hexane 30 min, UV 230 nm) retention times of
17.1 (minor) and 18.0min (major) 94 % ee with Rh_2(*S*-NTTL)$_4$.
17.1 (minor) and 18.0 min (major) 99 % ee with Rh_2(*S*-PTAD)$_4$.
17.8 (minor) and 18.8 min (major) 79 % ee with Rh_2(*S*-TPPTTL)$_4$.
17.6 (minor) and 18.5 min (major) 97 % ee with Rh_2(*S*-TCPTAD)$_4$.
17.4 (minor) and 18.3 min (major) -79 % ee with Rh_2(*S*-2-Cl-5-Br-TPCP)$_4$.

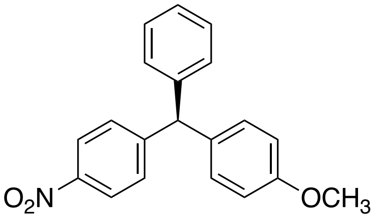

(*R*)-1-methoxy-4-((4-nitrophenyl)(phenyl)methyl)benzene (4)

Compound 4 was obtained through the oxidation of Compound **3.** Compound **3** was placed in a 16 mL dram vial charged with a stir bar. 1.2 equiv of 2,3-Dichloro-5,6-dicyano-1,4-benzoquinone (DDQ) was added and components were dissolved in 4 mL DCM. The reaction was complete after 2 h and the desired triarylmethane was observed by NMR. The crude mixture was filtered through a short pipette column to remove DDQ and desired product was obtained as an oil in 88 % yield (57 mg).

Compound **4** was also scaled up to a 1 mmol. To a flame-dried 50 mL round bottom flask charged with a stirbar Rh_2(*S*-PTAD)$_4$ (15.6 mg, 0.010 mmol, 1.0 mol %) and was added, and the reaction vial was purged with nitrogen three times and dissolved in CH_2Cl_2 (4.0 mL). The flask was placed in a salt/ice bath to reach –20 °C and was charged with 1,4-cyclohexadiene (0.38 mL,4 mmol, 4 equiv). In a 20mL vial 1-(diazo(4-methoxyphenyl)methyl)-4-nitrobenzene (269 mg, 1.00 mmol, 1 equiv) was dissolved in CH_2Cl_2 (16mL). The diazo solution was split into two 12mL syringes and was then added to the reaction vial dropwise over 1 h at (–20 °C) *via* a syringe pump. After 14 h, 2,3-dichloro-5,6-dicyano-1,4-benzoquinone (DDQ) (568mg, 2.5 equiv) was added into the mixture and allowed to stir for 2 h. The reaction was stopped, and filtered through a celite plug to remove excess DDQ. The mixture was purified *via* flash column chromatography with a gradient of 0 to 10% diethyl ether in hexanes to afford the desired triarylmethane compound in 90% yield (319 mg).
^{1}H NMR (400 MHz, $CDCl_3$) δ 8.13 (d, *J* = 8.7 Hz, 2H), 7.38 – 7.17 (m, 5H), 7.08 (d, *J* = 7.3 Hz, 2H), 7.00 (d, *J* = 8.4 Hz, 2H), 6.85 (d, *J* = 8.7 Hz, 2H), 5.58 (s, 1H), 3.79 (s, 3H).
^{13}C NMR (101 MHz, $CDCl_3$) δ 158.5, 152.0, 146.5, 142.7, 134.4, 130.3, 130.2, 129.3, 128.7, 126.9, 123.6, 114.1, 55.9, 55.3.
IR: 3027, 2932, 2835, 1605, 1510, 1345, 1302, 1248, 1178, 1032, 826 cm^{-1}.
HRMS ($^+$APCI) *m/z*: calcd for $C_{20}H_{17}NO_3$ $[M+H]^+$ 320.1286; found 320.1278.

$[\alpha]^{22}{}_{D}$ –2.7 (*c* 1.00, $CHCl_3$)
HPLC conditions: (OD column,1.0 mL/min 1% *i*-PrOH in *n*-hexane 30 min, UV 230 nm) retention times of 17.3 (major) and 22.5 min (minor) 99% ee with Rh_2(*S*-PTAD)$_4$.

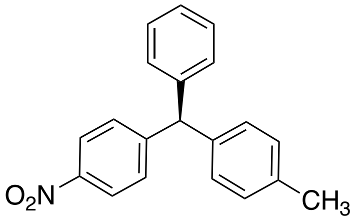

(*S*)-1-methyl-4-((4-nitrophenyl)(phenyl)methyl)benzene (5)

Compound 5 was obtained according to general procedure C from the C–H functionalization reaction between 1,4-cyclohexadiene (0.12 mL, 1.2 mmol, 4 equiv) and 1-(diazo(4-nitrophenyl)methyl)-4-methylbenzene (76 mg, 0.3 mmol, 1.0 equiv), catalyzed by Rh_2(*S*-PTAD)$_4$ (4.68 mg, 0.003 mmol, 1.0 mol %). The product was purified by flash column chromatography on silica gel (gradient elution: 0 – 5% diethyl ether in hexanes) to afford colorless oil in 81 % yield (73 mg).
^{1}H NMR (600 MHz, $CDCl_3$) δ 8.16 (d, *J* = 8.7 Hz, 2H), 7.37 – 7.25 (m, 5H), 7.16 (d, *J* = 7.9 Hz, 2H), 7.12 (d, *J* = 7.3 Hz, 2H), 7.01 (d, *J* = 8.1 Hz, 2H), 5.63 (s, 1H), 2.37 (s, 3H).
^{13}C NMR (151 MHz, $CDCl_3$) δ 151.9, 146.5, 142.5, 139.4, 136.6, 130.2, 129.4, 129.3, 129.2, 128.7, 126.9, 123.6, 56.3, 21.0.
IR: 3025, 2921, 1593, 1514, 1450, 1343, 1110, 820, 778 cm^{-1}.
HRMS ($^+$APCI) *m/z*: calcd for $C_{20}H_{17}NO_2[M+H]^+$ 304.1338; found 304.1334.
$[\alpha]^{23}{}_{D}$ 5.5 (*c* 1.28, $CHCl_3$)
HPLC conditions: HPLC (ODH column, 1.0 mL/min 0.5% *i*-PrOH in *n*-hexane 45 min, UV 230 nm) retention times of 12.3 (major) and 16.0 min (minor) 94% ee with Rh_2(S-PTAD)$_4$.

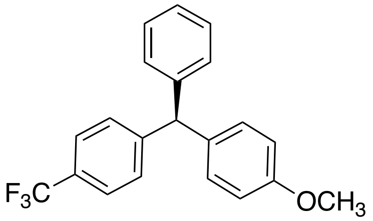

(*R*)-1-methoxy-4-(phenyl(4-(trifluoromethyl)phenyl)methyl)benzene (6)

Compound 6 was obtained according to general procedure C from the C–H functionalization reaction between 1,4-cyclohexadiene (0.12 mL, 1.2 mmol, 4 equiv) and 1-(diazo(4-(trifluoromethyl)phenyl)methyl)-4-methoxybenzene (88 mg, 0.3 mmol, 1.0 equiv), catalyzed by Rh_2(*S*-PTAD)$_4$ (4.68 mg, 0.003 mmol, 1.0 mol %). The product was purified by flash column chromatography on silica gel (gradient elution: 0 – 5% diethyl ether in hexanes) to afford **6** as a colorless oil in 80 % yield (83 mg).
^{1}H NMR: (600 MHz, $CDCl_3$) δ 7.55 (d, *J* = 8.2 Hz, 1H), 7.32 (t, *J* = 7.3 Hz, 1H), 7.30 – 7.23 (m, 1H), 7.11 (d, *J* = 7.3 Hz, 1H), 7.03 (d, *J* = 8.7 Hz, 1H), 6.86 (d, *J* = 8.7 Hz, 1H). This matches literature reported values.[4]
$[\alpha]^{22}{}_{D}$ –3.4 (*c* 1.22, $CHCl_3$)
HPLC (ODH column, hexane, 1.0 mL/min 0.5% *i*-PrOH in *n*-hexane 25 min, UV 230 nm) retention times of 6.2 (minor) and 7.2 min (major) 85 % ee with Rh_2(S-PTAD)$_4$.

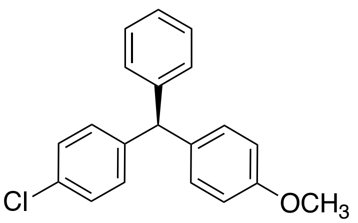

(*S*)-1-chloro-4-((4-methoxyphenyl)(phenyl)methyl)benzene (7)

Compound 7 was obtained according to general procedure C from the C–H functionalization reaction between 1,4-cyclohexadiene (0.12 mL, 1.2 mmol, 4 equiv) and 1-chloro-4-(diazo(4-methoxyphenyl)methyl)benzene (78 mg, 0.3 mmol, 1.0 equiv), catalyzed by Rh_2(*S*-PTAD)$_4$ (4.68 mg, 0.003 mmol, 1.0 mol %). The product was purified by flash column chromatography on silica gel (gradient elution: 0 – 5% diethyl ether in hexanes) to afford colorless oil in 61 % yield (57 mg).
^{1}H NMR (600 MHz, $CDCl_3$) δ 7.31 – 7.22 (m, 5H), 7.11 (d, *J* = 7.6 Hz, 2H), 7.06 (d, *J* = 8.3 Hz, 2H), 7.02 (d, *J* = 8.6 Hz, 2H), 6.86 (d, *J* = 8.6 Hz, 2H), 5.49 (s, 1H), 3.81 (s, 3H).
^{13}C NMR (151 MHz, $CDCl_3$) δ 158.2, 143.7, 142.8, 135.6, 132.1, 130.7, 130.5, 130.3, 129.3, 128.4, 126.5, 113.8, 55.4, 55.3.
IR: 3026, 2930, 2834, 1509, 1488, 1248, 1177, 1034, 819, 700 cm^{-1}.
HRMS ($^+$APCI) *m/z*: calcd for $C_{20}H_{17}ClO$ [M^+] 308.0968; found 308.0966.
HPLC conditions: HPLC (ODH column, hexane, 0.8 mL/min 0.2 *i*-PrOH in *n*-hexane 20 min, UV 230 nm) retention times of 14.4 (minor) and 15.6 min (major) 79 % ee with Rh_2(S-PTAD)$_4$.

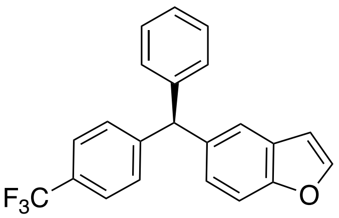

(*R*)-5-(phenyl(4-(trifluoromethyl)phenyl)methyl)benzofuran (8)

Compound 8 was obtained according to general procedure C from the C–H functionalization reaction between 1,4-cyclohexadiene (0.12 mL, 1.2 mmol, 4 equiv) and 5-(diazo(4-(trifluoromethyl)phenyl)methyl)benzofuran (91 mg, 0.3 mmol, 1.0 equiv), catalyzed by Rh_2(*S*-PTAD)$_4$ (4.68 mg, 0.003 mmol, 1.0 mol %). The product was purified by flash column chromatography on silica gel (gradient elution: 0 – 5% diethyl ether in hexanes) to afford colorless oil in 86% yield (91 mg).
^{1}H NMR (600 MHz, $CDCl_3$) δ 7.63 (d, *J* = 2.2 Hz, 1H), 7.56 (d, *J* = 7.9 Hz, 2H), 7.45 (d, *J* = 8.5 Hz, 1H), 7.33 (t, *J* = 7.6 Hz, 2H), 7.27-7.24 (m, 5 H), 7.14 (d, *J* = 7.6 Hz, 2H), 7.09 (dd, *J* = 8.5, 1.89 Hz, 1H), 6.71 (d, *J* = 2.2 Hz, 1H), 5.73 (s, 1H).
^{13}C NMR (151 MHz, $CDCl_3$) δ 153.8, 145.5, 143.3, 137.6, 129.8, 129.4, 128.7, 128.5, 127.6, 126.7, 125.9, 125.28, 125.25 (q, *J* = 3.8 Hz), 125.23, 121.7, 111.3, 106.6, 56.5.
^{19}F NMR (565 MHz, $CDCl_3$) δ -62.4.
IR: 2927, 2855, 1708, 1617, 1494, 1466, 1324, 1163, 1121, 1108, 1066, 1030, 1018, 887, 851 cm^{-1}.
$[\alpha]^{22}_D$ –6.8 (*c* 1.00, $CHCl_3$)
HRMS ($^+$APCI) *m/z*: calcd for $C_{22}H_{15}F_3O$ $[M+H]^+$ 353.1153; found 353.1167.
HPLC conditions: The triarylmethane **8** could not be resolved by chiral HPLC. Chiral HPLC analysis was conducted on a small aliquot of the C–H functionalization products, prior to DDQ oxidation to from **8**. HPLC (ADH column, hexane, 0.5 Ml/min, 1.0% *i*-PrOH in *n*-hexane 30 min, UV 230) retention times of 10.5 min (minor) and 11.3 min (major) 40 % ee with Rh_2(S-PTAD)$_4$.

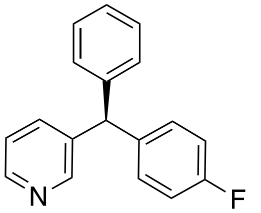

(*S*)-3-((4-fluorophenyl)(phenyl)methyl)pyridine (9)

Compound 9 was obtained according to general procedure C from the C–H functionalization reaction between 1,4-cyclohexadiene (0.12 mL, 1.2 mmol, 4 equiv) and 3-(diazo(4-fluorophenyl)methyl)pyridine (64 mg, 0.3 mmol, 1.0 equiv), catalyzed by Rh_2(*S*-PTAD)4 (4.68 mg, 0.003 mmol, 1.0 mol %). The product was purified by flash column chromatography on silica gel (gradient elution: 0 – 5% diethyl ether in hexanes) to afford colorless oil in 81 % yield (64 mg).
^{1}H NMR (600 MHz, $CDCl_3$) δ 8.55 (d, *J* = 5.2 Hz, 2H), 7.37 – 7.31 (m, 2H), 7.30 – 7.27 (m, 1H), 7.14 – 6.99 (m, 8H), 5.51 (s, 1H).
^{13}C NMR (151 MHz, $CDCl_3$) δ 162.5, 160.9, 152.6, 149.9, 141.9, 137.9 (d, *J* = 3.1 Hz), 130.8 (d, *J* = 7.9 Hz), 129.2, 128.7, 128.6, 127.0, 124.5, 115.5, 115.4, 55.5.
^{19}F NMR (565 MHz, $CDCl_3$) δ -115.8.
IR: 3026, 2922, 2850, 1594, 1506, 1412, 1224, 1159, 1014, 816, 788 cm^{-1}.
HRMS ($^{+}$APCI) *m/z*: calcd for $C_{18}H_{14}FN[M+H]^+$ 264.1188; found 264.1187.
HPLC conditions: The triarylmethane **9** could not be resolved by chiral HPLC. Chiral HPLC analysis was conducted on a small aliquot of the C–H functionalization products, prior to DDQ oxidation to from **9**. HPLC (ODH column, 1.0 mL/min 2.25 % *i*-PrOH in *n*-hexane 30 min, UV 230 nm) retention times of 16.2 (major) and 25.0 min (minor) 77 % ee with Rh_2(*S*-PTAD*)*$_4$.

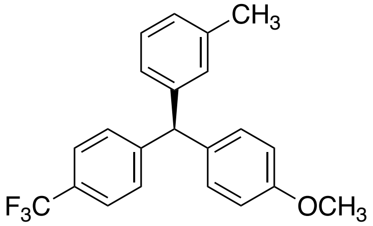

(*R*)-1-((4-methoxyphenyl)(4-(trifluoromethyl)phenyl)methyl)-3-methylbenzene (12)

Compound 12 was obtained according to general procedure C from the C–H functionalization reaction between 1-methyl,1,4-cyclohexadiene (0.1mL, 1.2 mmol, 4 equiv) and 1-(diazo(4-(trifluoromethyl)phenyl)methyl)-4-methoxybenzene (88 mg, mg, 0.3 mmol, 1.0 equiv), catalyzed by Rh_2(*S*-PTAD)$_4$ (4.68 mg, 0.003 mmol, 1.0 mol %). The product was purified by flash column chromatography on silica gel (gradient elution: 0 – 5% diethyl ether in hexanes) to afford colorless oil in 85 % yield (91 mg).
^{1}H NMR (600 MHz, $CDCl_3$) δ 7.52 (d, *J* = 8.0 Hz, 2H), 7.22 (d, *J* = 8.0 Hz, 2H), 7.18 (t, *J* = 7.5 Hz, 1H), 7.04 (d, *J* = 7.5 Hz, 1H), 7.00 (d, *J* = 8.7 Hz, 2H), 6.91 (s, 1H), 6.86 (d, *J* = 7.5 Hz, 1H), 6.84 (d, *J* = 8.7 Hz, 2H), 5.50 (s, 1H), 3.79 (s, 3H), 2.29 (s, 3H).
^{13}C NMR (151 MHz, $CDCl_3$) δ 158.3, 148.5, 143.2, 138.1, 135.2, 130.3, 130.0, 129.7, 128.4, 127.4, 126.4, 125.2, 125.24(q, *J* = 3.71 Hz), 125.22, 125.19, 113.9, 55.8, 55.3, 21.5.
^{19}F NMR (565 MHz, $CDCl_3$) δ -62.4.
IR: 2930, 1606, 1510, 1323, 1250, 1067, 1035, 834, 792 cm^{-1}.
HRMS ($^{-}$APCI) *m/z*: calcd for $C_{22}H_{18}F_3O$ [M^-] 355.1315; found 355.1308.
$[\alpha]^{23}_{D}$ –10.7 (*c* 1.00, $CHCl_3$)
HPLC conditions: HPLC (ODH column, 1.0 mL/min 1% *i*-PrOH in *n*-hexane 25 min, UV 230 nm) retention times of 4.8 (minor) and 5.3 min (major) 79 % ee with Rh_2(S-PTAD)$_4$.

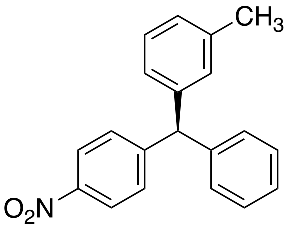

(*S*)-1-Methyl-3-((4-nitrophenyl)(phenyl)methyl)benzene (13)

Compound 13 was obtained according to general procedure C from the C–H functionalization reaction between 1-methyl,1,4-cyclohexadiene (0.1 mL, 1.2 mmol, 4 equiv) 1-(diazo(phenyl)methyl)-4-nitrobenzene (72 mg, 0.3 mmol, 1.0 equiv), catalyzed by Rh_2(*S*-PTAD)$_4$ (4.68 mg, 0.003 mmol, 1.0 mol %). The product was purified by flash column chromatography on silica gel (gradient elution: 0 – 5% diethyl ether in hexanes) to afford **13** as a colorless oil in 89 % yield (81 mg).
^{1}H NMR (600 MHz, $CDCl_3$) δ 8.14 (d, *J* = 8.7 Hz, 2H), 7.35 – 7.24 (m, 5H), 7.21 (t, *J* = 7.6 Hz, 1H), 7.12 – 7.06 (m, 3H), 6.92 (s, 1H), 6.88 (d, *J* = 7.9 Hz, 1H), 5.60 (s, 1H), 2.31 (s, 3H).
^{13}C NMR (151 MHz, $CDCl_3$) δ 151.8, 146.5, 142.4, 142.3, 138.4, 130.3, 130.1, 129.3, 128.7, 128.6, 127.7, 126.9, 126.4, 123.6, 56.7, 21.5.
IR: 3026, 2902, 1602, 1514,1343, 1108, 1076, 1030, 1015, 857, 748 cm^{-1}.
HRMS ($^+$APCI) *m/z*: calcd for $C_{20}H_{17}NO_2$ $[M+H]^+$ 304.1337; found 304.1329.
$[\alpha]^{23}_D$ –5.1 (*c* 1.09 $CHCl_3$)
HPLC conditions: HPLC (OD column, 1.0 mL/min 0.5 % *i*-PrOH in *n*-hexane 30 min, UV 210 nm) retention times of 13.5 (minor) and 16.0 min (major) 76 % ee with Rh_2(S-PTAD)$_4$.

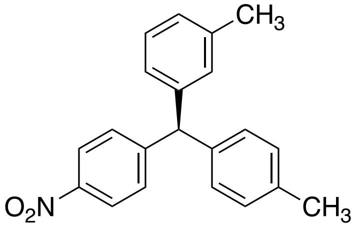

(*S*)-1-Methyl-3-((4-nitrophenyl)(*p*-tolyl)methyl)benzene (14)

Compound 14 was obtained according to general procedure C from the C–H functionalization reaction between 1-methyl,1,4-cyclohexadiene (0.1 mL, 1.2 mmol, 4 equiv) 1-(diazo(4-nitrophenyl)methyl)-4-methylbenzene (76mg, 0.3 mmol, 1.0 equiv), catalyzed by Rh_2(*S*-PTAD)$_4$ (4.68 mg, 0.003 mmol, 1.0 mol %). The product was purified by flash column chromatography on silica gel (gradient elution: 0 – 5% diethyl ether in hexanes) to afford colorless oil in 51% yield (48 mg).
^{1}H NMR (600 MHz, $CDCl_3$) δ 8.16 (d, *J* = 8.7 Hz, 2H), 7.30 (d, *J* = 8.7 Hz, 2H), 7.22 (t, *J* = 7.6 Hz, 1H), 7.15 (d, *J* = 7.8 Hz, 2H), 7.09 (d, *J* = 7.5 Hz, 1H), 7.00 (d, *J* = 8.0 Hz, 2H), 6.94 (s, 1H), 6.89 (d, *J* = 7.6 Hz, 1H), 5.58 (s, 1H), 2.37 (s, 3H), 2.33 (s, 3H).
^{13}C NMR (151 MHz, $CDCl_3$) δ 152.0, 146.5, 142.5, 139.4, 138.3, 136.6, 130.2, 130.0, 129.4, 129.2, 128.5, 127.7, 126.4, 123.5, 56.3, 21.5, 21.0.
IR: 3021, 2920, 1603, 1514, 1489, 1455, 1343, 1109, 841, 763 cm^{-1}.
$[\alpha]^{23}_D$ –3.9 (*c* 0.75, $CHCl_3$)
HRMS ($^+$APCI) *m/z*: calcd for $C_{21}H_{19}NO_2$ $[M+H]^+$ 318.1494; found 318.1491.
HPLC conditions: The e.e. value is an estimated value due to the imperfect resolution of peaks in the HPLC. (ADH column, 0.5 mL/min 0.5% *i*-PrOH in *n*-hexane 25 min, UV 230 nm) retention times of 16.3 (major) and 17.1 min (minor) 76 % ee with Rh_2(S-PTAD)$_4$.

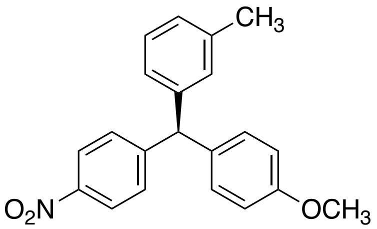

(*R*)-1-((4-methoxyphenyl)(4-nitrophenyl)methyl)-3-methylbenzene(15)

Compound 15 was obtained according to general procedure C from the C–H functionalization reaction between 1-methyl,1,4-cyclohexadiene (0.1 mL, 1.2 mmol, 4 equiv) and 1-(diazo(4-methoxyphenyl)methyl)-4-nitrobenzene (81 mg, 0.3 mmol, 1.0 equiv), catalyzed by Rh_2(*S*-PTAD)$_4$ (4.68 mg, 0.003 mmol, 1.0 mol %). The product was purified by flash column chromatography on silica gel (gradient elution: 0 – 5% diethyl ether in hexanes) to afford colorless oil in 83 % yield (83 mg).
^{1}**H NMR** (600 MHz, $CDCl_3$) δ 8.16 (d, *J* = 8.8 Hz, 2H), 7.30 (s, 1H), 7.28 (d, *J* = 2.9 Hz, 2H), 7.22 (t, *J* = 7.6 Hz, 1H), 7.09 (d, *J* = 7.6 Hz, 1H), 7.02 (d, *J* = 8.6 Hz, 2H), 6.92 (s, 1H), 6.87 (d, *J* = 8.6 Hz, 2H), 5.56 (s, 1H), 3.82 (s, 3H), 2.32 (s, 3H).
^{13}**C NMR** (151 MHz, $CDCl_3$) δ 158.4, 152.2, 146.5, 142.6, 138.3, 134.5, 130.3, 130.2, 130.0, 128.5, 127.6, 126.3, 123.5, 114.0, 55.8, 55.3, 21.5.
IR: 2930, 1605, 1489, 1346,1302,1249, 1179, 1110, 849 cm^{-1}.
HRMS ($^{+}$APCI) *m/z*: calcd for $C_{21}H_{19}NO_3[M+H]^+$ 334.1443; found 334.1439.
$[\alpha]^{22}_{D}$ –6.6 (*c* 1.00, $CHCl_3$)
HPLC conditions: HPLC (ODH column, 0.8 mL/min 2.25 *i*-PrOH in *n*-hexane 30 min, UV 230 nm) retention times of:
12.4 (major) and 14.1 min (minor) 93 % ee with Rh_2(S-PTAD)$_4$.
12.3 (major) and 14.0 min (minor) 91 % ee with Rh_2(S-NTTL)$_4$.
12.3 (major) and 14.0 min (minor) 77 % ee with Rh_2(S-TPPTTL)$_4$.
12.3 (major) and 14.0 min (minor) 84 % ee with Rh_2(S-PTTL)$_4$.

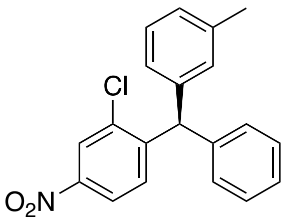

(*R*)-2-chloro-4-nitro-1-(phenyl(*m*-tolyl)methyl)benzene(17)

Compound 17 was obtained according to general procedure C from the C–H functionalization reaction between 1-methyl, 1,4-cyclohexadiene (0.1mL, 1.2 mmol, 4 equiv) and 2-chloro-1-(diazo(phenyl)methyl)-4-nitrobenzene (82 mg, 0.3 mmol, 1.0 equiv), catalyzed by Rh_2(*S*-TPPTTL)$_4$ (4.93 mg, 0.003 mmol, 1.0 mol %). The product was purified by flash column chromatography on silica gel (gradient elution: 0 – 2% diethyl ether in pentane) to afford colorless oil in 94 % yield (96 mg).
^{1}**H NMR** (600 MHz, $CDCl_3$) δ 8.30 (d, *J* = 2.4 Hz, 1H), 8.05 (dd, *J* = 8.6, 2.36 Hz, 1H), 7.35 (t, *J* = 7.5 Hz, 2H), 7.32 – 7.27 (m, 1H), 7.23 (t, *J* = 7.6 Hz, 1H), 7.17 (d, *J* = 8.6 Hz, 1H), 7.11 (d, *J* = 7.5 Hz, 1H), 7.07 (d, *J* = 7.4 Hz, 2H), 6.90 (s, 1H), 6.85 (d, *J* = 7.6 Hz, 1H), 5.98 (s, 1H), 2.33 (s, 3H).
^{13}**C NMR** (151 MHz, $CDCl_3$) δ 149.3, 146.8, 141.1, 140.9, 138.4, 135.5, 131.7, 130.1, 129.4, 128.7, 128.6, 127.9, 127.1, 126.5, 124.9, 121.5, 53.6, 21.5.
IR: 3027, 2922, 1600, 1518,1395, 1137, 1045, 892 cm^{-1}.

[α]$^{22}_{D}$ 16.9 (*c* 0.83, $CHCl_3$)
HRMS ($^+$APCI) *m/z*: calcd for $C_{20}H_{16}ClNO_2$ $[M+H]^+$ 338.0948; found 338.0946.
HPLC conditions: (ODH column, 0.8 mL/min 0.3 % *i*-PrOH in *n*-hexane 30 min, UV 230 nm) retention times of:
17.1 min (minor) and 23.3 min (major) 74 % ee with Rh_2(*S*-PTAD)$_4$.
17.2 (minor) and 22.9 min (major) 6 % ee with Rh_2(*S*-TCPTAD)$_4$.
17.3 (major) and 22.5 min (minor) -6 % ee with Rh_2(*S*-NTTL)$_4$.
17.8 (minor) and 22.6 min (major) 51 % ee with Rh_2(*S*-PTTL)$_4$.
18.2 (minor) and 22.7 min (major) 88 % ee with Rh_2(*S*-TPPTTL)$_4$.

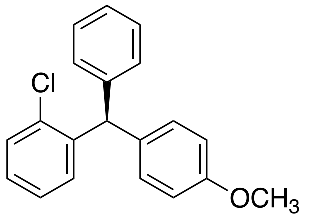

(*S*)-1-chloro-2-((4-methoxyphenyl)(phenyl)methyl)benzene(18)

Compound 18 was obtained according to general procedure C from the C–H functionalization reaction between 1,4-cyclohexadiene (0.12 mL, 1.2 mmol, 4 equiv) and 1-chloro-2-(diazo(4-methoxyphenyl)methyl)benzene (78 mg, 0.3 mmol, 1.0 equiv), catalyzed by Rh_2(*S*-TPPTTL)$_4$ (4.93 mg, 0.003 mmol, 1.0 mol %). The product was purified by flash column chromatography on silica gel (gradient elution: 0 – 2% diethyl ether in pentane) to afford colorless oil in 82 % yield (76 mg).
^{1}H NMR (600 MHz, $CDCl_3$) δ 7.43 – 7.38 (m, 1H), 7.31 (t, *J* = 7.5 Hz, 2H), 7.25 (t, *J* = 7.3 Hz, 1H), 7.21 – 7.18 (m, 2H), 7.10 (d, *J* = 7.4 Hz, 2H), 7.02 (d, *J* = 8.7 Hz, 2H), 6.99 – 6.95 (m, 1H), 6.86 (d, *J* = 8.7 Hz, 2H), 5.94 (s, 1H), 3.82 (s, 3H).
^{13}C NMR (151 MHz, $CDCl_3$) δ 158.2, 143.0, 141.9, 134.7, 134.5, 131.1, 130.5, 129.7, 129.5, 128.3, 127.7, 126.6, 126.4, 113.7, 55.2, 52.6.
IR: 3060, 3025, 2930, 2834, 1608, 1508, 1450, 1246, 1177, 1050, 1035 cm^{-1}.
HRMS ($^+$APCI) *m/z*: calcd for $C_{20}H_{17}ClO$ $[M]^+$ 308.0968 found 308.0965.
[α]$^{22}_{D}$ –8.8 (*c* 1.06, $CHCl_3$)
HPLC conditions: The triarylmethane **18** could not be resolved by chiral HPLC. Chiral HPLC analysis was conducted on a small aliquot of the C–H functionalization products, prior to DDQ oxidation to from **18.** (ADH column, 0.5 mL/min 0.5 % *i*-PrOH in *n*-hexane 30 min, UV 230 nm) retention times of 13.9 (major) and 14.9 min (minor) 87 % ee with Rh_2(*S*-TPPTTL)$_4$, and retention times of 13.9 (major) and 15.8 min (minor) 98 % ee with Rh_2(*S*-PTAD)$_4$.

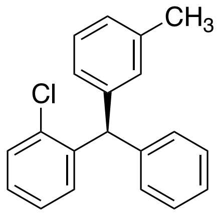

(*R*)-1-chloro-2-(phenyl(*m*-tolyl)methyl)benzene(19)

Compound 19 was obtained according to general procedure C from the C–H functionalization reaction between 1-methyl, 1,4-cyclohexadiene (0.1mL, 1.2 mmol, 4 equiv) and 1-chloro-2-(diazo(phenyl)methyl)benzene (69 mg, 0.3 mmol, 1.0 equiv), catalyzed by Rh_2(*S*-TPPTTL)$_4$ (4.93 mg, 0.003 mmol, 1.0 mol %). The product was purified by flash column chromatography on silica gel (gradient elution: 0 – 2% diethyl ether in pentane) to afford colorless oil in67 % yield (59 mg).

^{1}H NMR (400 MHz, $CDCl_3$) δ 7.46 – 7.39 (m, 1H), 7.34 (t, *J* = 7.5 Hz, 2H), 7.30 – 7.18 (m, 4H), 7.15 – 7.06 (m, 3H), 7.03 – 6.98 (m, 1H), 6.96 (s, 1H), 6.90 (d, *J* = 7.9 Hz, 1H), 5.98 (s, 1H), 2.34 (s, 3H).
^{13}C NMR (151 MHz, $CDCl_3$) δ 142.7, 142.5, 141.7, 138.0, 134.6, 131.2, 130.4, 129.7, 129.6, 128.4, 128.2, 127.7, 127.3, 126.64, 126.59, 126.5, 53.4, 21.5.
HRMS ($^{+}$APCI) *m/z*: calcd for $C_{20}H_{17}Cl[M]^+$ 292.1019 found 292.1013.
IR: 3059, 3024, 2919, 1600, 1589, 1493, 1467, 1125, 1050, 1039 cm^{-1}.
$[\alpha]^{23}_{D}$ 2.1 (*c* 1.55, $CHCl_3$)
HPLC conditions: The triarylmethane **19** could not be resolved by chiral HPLC. Chiral HPLC analysis was conducted on a small aliquot of the C–H functionalization products, prior to DDQ oxidation to from **19**.
HPLC (ODH column, 0.8 mL/min .1 % i-PrOH in n-hexane 45 min, UV 230 nm) retention times of 9.5 (major) and 13.1 min (minor) 85 % ee with Rh_2(S-TPPTTL)$_4$ for the major diastereomer; 11 .6 (major) and 15.2 min (minor) 70 % ee with Rh_2(S-TPPTTL)$_4$ for the minor diastereomers.
The major enantiomer was assigned to the major peak based on the pattern shown in Scheme 5 and on the crystal structure obtained from compound **11a.** The crude C-H insertion (both diastereomers) were oxidized to the final triarylmethane compound **19**. To account for the minor diastereomer, we obtain a calculated ee of 83% based on the 90:10 ratio and an estimated ee value of 83% was assigned.

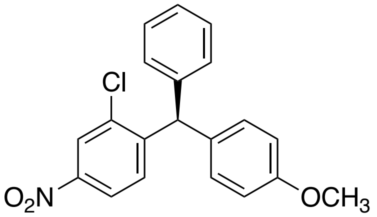

(*S*)-2-chloro-1-((4-methoxyphenyl)(phenyl)methyl)-4-nitrobenzene(20)

Compound 20 was obtained according to general procedure C from the C–H functionalization reaction between 1,4-cyclohexadiene (0.12mL, 1.2 mmol, 4 equiv) 2-chloro-1-(diazo(4-methoxyphenyl)methyl)-4-nitrobenzene (92 mg, 0.3 mmol, 1.0 equiv), catalyzed by Rh_2(*S*-TPPTTL)$_4$ (4.93 mg, 0.003 mmol, 1.0 mol %). The product was purified by flash column chromatography on silica gel (gradient elution: 0 – 2% diethyl ether in pentane) to afford colorless oil in 81 % yield (90 mg).
^{1}H NMR (600 MHz, $CDCl_3$) δ 8.29 (d, *J* = 2.4 Hz, 1H), 8.05 (dd, *J* = 8.6, 2.4 Hz, 1H), 7.35 (t, *J* = 7.4 Hz, 2H), 7.29 (t, *J* = 7.5 Hz, 1H), 7.17 (d, *J* = 8.6 Hz, 1H), 7.08 (d, *J* = 7.3 Hz, 2H), 6.99 (d, *J* = 8.7 Hz, 2H), 6.89 (d, *J* = 8.7 Hz, 2H), 5.96 (s, 1H), 3.83 (s, 3H).
^{13}C NMR (151 MHz, $CDCl_3$) δ 158.6, 149.6, 146.8, 141.4, 135.4, 133.0, 131.6, 130.4, 129.3, 128.7, 127.1, 124.9, 121.5, 114.1, 55.3, 52.9.
IR: 3027, 2931, 2835, 1608, 1509, 1346, 1247, 1177, 1360, 1031, 824,736 cm^{-1}.
HRMS ($^{+}$APCI) *m/z*: calcd for $C_{20}H_{16}ClNO_3[M+H]^+$ 354.0897 found 354.0889.
$[\alpha]^{23}_{D}$ –9.4 (*c* 1.00, $CHCl_3$)
HPLC conditions: HPLC (ODH column, 0.25 mL/min 2.0 % *i*-PrOH in *n*-hexane 60 min, UV 230 nm) retention times of 35.0 (minor) and 41.9 min (major) 98 % ee with Rh_2(*S*-TPPTTL)$_4$.

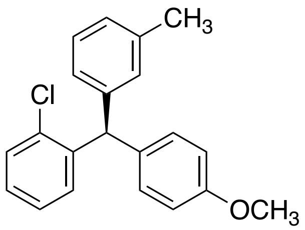

(*R*)-1-chloro-2-((4-methoxyphenyl)(*m*-tolyl)methyl)benzene(21)

Compound 21 was obtained according to general procedure C from the C–H functionalization reaction between 1-methyl, 1,4-cyclohexadiene (0.1mL, 1.2 mmol, 4 equiv) and 1-chloro-2-(diazo(4-methoxyphenyl)methyl)benzene (78 mg, 0.3 mmol, 1.0 equiv), catalyzed by Rh_2(*S*-TPPTTL)$_4$ (4.93 mg, 0.003 mmol, 1.0 mol %). The product was purified by flash column chromatography on silica gel (gradient elution: 0 – 2% diethyl ether in pentane) to afford colorless oil in 79 % yield (77 mg).

^{1}H NMR (600 MHz, $CDCl_3$) δ 7.42 – 7.38 (m, 1H), 7.22 – 7.18 (m, 3H), 7.07 (d, *J* = 7.5 Hz, 1H), 7.02 (d, *J* = 8.6 Hz, 2H), 7.00 – 6.97 (m, 1H), 6.94 (s, 1H), 6.90 – 6.85 (m, 3H), 5.91 (s, 1H), 3.82 (s, 3H), 2.33 (s, 3H).

^{13}C NMR (151 MHz, $CDCl_3$) δ 158.1, 142.9, 142.0, 137.9, 134.8, 134.5, 131.1, 130.5, 130.2, 129.7, 128.2, 127.6, 127.2, 126.55, 126.52, 113.7, 55.2, 52.5, 21.5.

IR: 3003, 2927, 2834, 1608, 1509, 1465, 1440,1281, 1247, 1177, 1037 cm^{-1}.

HRMS ($^{+}$APCI) *m/z*: calcd for $C_{21}H_{19}ClO[M+H]^+$ 323.1203 found 323.1198.

HPLC conditions: The triarylmethane **21** could not be resolved by chiral HPLC. Chiral HPLC analysis was conducted on a small aliquot of the C–H functionalization products, prior to DDQ oxidation to from **21**. HPLC (ADH column, 0.25 mL/min 0.5 % i-PrOH in n-hexane 45 min, UV 230 nm) retention times of 23.0 min (major) and 24.1 min, 26.7 min and 29.5 min as minor peaks with Rh_2(S-TPPTTL)$_4$. The ratio of peaks is 91.5:1.7:2.3: 4.5. The major diastereomer is tentatively assigned as (*S,R*), assuming the same stereochemical outcome as was observed in Scheme 5. Assuming all four stereoisomers are converted to the triarylmethane **21**, the absolute configuration of **21** is assigned as *R* and is formed in >83% ee (switch from *S* to *R* caused by switch in priority sequence).

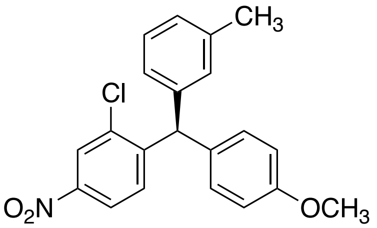

(*R*)-2-chloro-1-((4-methoxyphenyl)(*m*-tolyl)methyl)-4-nitrobenzene (22)

Compound 22 was obtained according to general procedure C from the C–H functionalization reaction between 1-methyl, 1,4-cyclohexadiene (0.1mL, 1.2 mmol, 4 equiv) 2-chloro-1-(diazo(4-methoxyphenyl)methyl)-4-nitrobenzene (92 mg, 0.3 mmol, 1.0 equiv), catalyzed by Rh_2(*S*-TPPTTL)$_4$ (4.93 mg, 0.003 mmol, 1.0 mol %). The product was purified by flash column chromatography on silica gel (gradient elution: 0 – 2% diethyl ether in pentane) to afford colorless oil in 86% yield (95 mg).

^{1}H NMR (600 MHz, $CDCl_3$) δ 8.29 (d, *J* = 2.3 Hz, 1H), 8.05 (dd, *J* = 8.7, 2.35 Hz, 1H), 7.22 (t, *J* = 7.61 Hz, 1H), 7.17 (d, *J* = 8.6 Hz, 1H), 7.10 (d, *J* = 7.5 Hz, 1H), 6.98 (d, *J* = 8.5 Hz, 2H), 6.88 (d, *J* = 8.8 Hz, 2H), 6.84 (d, *J* = 7.7 Hz, 1H), 5.92 (s, 1H), 3.82 (s, 3H), 2.33 (s, 3H).

^{13}C NMR (151 MHz, $CDCl_3$) δ 158.6, 149.7, 146.8, 141.3, 138.4, 135.4, 133.1, 131.6, 130.4, 130.1, 128.5, 127.8, 126.4, 124.9, 121.5, 114.1, 55.3, 52.8, 21.5.

IR: 2929,2836, 1607, 1510, 1462, 1348, 1248, 1178, 1034, 892 cm^{-1}.

HRMS ($^+$APCI) *m/z*: calcd for $C_{21}H_{18}ClNO_3[M+H]^+$ 368.1053 found 368.1048.
$[\alpha]^{23}_D$ –4.1 (*c* 1.00, $CHCl_3$)
HPLC conditions: HPLC (ODH column, 1.0mL/min 1.0% *i*-PrOH in *n*-hexane 15 min, UV 230 nm) retention times of 9.5 (minor) and 11.0 min (major) 91% ee with Rh_2(*S*-TPPTTL)$_4$.

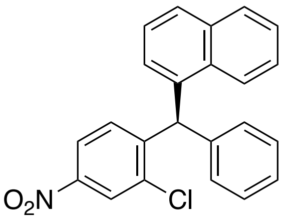

(*R*)-1-((2-chloro-4-nitrophenyl)(phenyl)methyl)naphthalene (23)

Compound 23 was obtained according to general procedure C from the C–H functionalization reaction between 1,8a-dihydronaphthalene (0.16mL, 1.2 mmol, 4 equiv) and 2-chloro-1-(diazo(phenyl)methyl)-4-nitrobenzene (82mg, 0.3 mmol, 1.0 equiv), catalyzed by Rh_2(*S*-TPPTTL)4 (4.93 mg, 0.003 mmol, 1.0 mol %). The product was purified by flash column chromatography on silica gel (gradient elution: 0 – 10% diethyl ether in pentane) to afford an oil in 43% yield (49 mg).
^{1}H NMR (600 MHz, $CDCl_3$) δ 8.34 (d, *J* = 2.4 Hz, 1H), 7.99 (dd, *J* = 8.6, 2.4 Hz, 1H), 7.91 (d, *J* =7.9 Hz, 1H), 7.83 (t, *J* = 7.6 Hz, 2H), 7.50 (ddd, *J* = 8.2, 6.8, 1.3 Hz, 1H), 7.46 (ddd, *J* = 7.6, 6.4, 1.3 Hz, 1H), 7.42 – 7.30 (m, 4H), 7.11 (d, *J* = 6.9 Hz, 2H), 7.07 (d, *J* = 8.6 Hz, 1H), 6.89 (d, *J* = 7.2 Hz, 1H), 6.65 (s, 1H).
^{13}C NMR (151 MHz, $CDCl_3$) δ 149.1, 147.0, 140.5, 137.5, 135.3, 134.1, 131.7, 131.5, 129.7, 129.0, 128.9, 128.2, 127.3, 127.2, 126.7, 125.9, 125.2, 125.0, 123.6, 121.6, 50.4.
IR: 3063, 2962, 2358,1738, 1558,1395, 1136, 894 cm^{-1}.
HRMS ($^+$APCI) *m/z*: calcd for $C_{23}H_{16}ClNO_2$ $[M+H]^+$ 374.0948 found 374.0942.
$[\alpha]^{22}_D$ 3.5 (*c* 1.32, $CHCl_3$)
HPLC conditions: HPLC (ADH column, 1.0 mL/min 1% *i*-PrOH in *n*-hexane 30 min, UV 230 nm) retention times of 10.5 (minor) min and 12.9 (major) min with 65 % ee Rh_2(*S*- TPPTTL)$_4$.

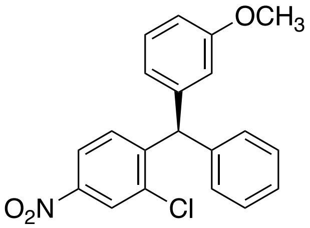

(*R*)-2-chloro-1-((3-methoxyphenyl)(phenyl)methyl)-4-nitrobenzene (24)

Compound 24 was obtained according to general procedure C from the C–H functionalization reaction between 1-methoxycyclohexa-1,4-diene (0.16mL, 1.2 mmol, 4 equiv) and 2-chloro-1-(diazo(phenyl)methyl)-4-nitrobenzene (82mg, 0.3 mmol, 1.0 equiv), catalyzed by Rh_2(*S*-TPPTTL)4 (4.93 mg, 0.003 mmol, 1.0 mol %). The product was purified by flash column chromatography on silica gel (gradient elution: 0 – 5% diethyl ether in pentane) to afford an oil in 33% yield (36 mg).
^{1}H NMR (400 MHz, $CDCl_3$) δ 8.29 (d, *J* = 2.4 Hz, 1H), 8.05 (dd, *J* = 8.6, 2.37 Hz, 1H), 7.37 – 7.26 (m, 4H), 7.17 (d, *J* = 8.6 Hz, 1H), 7.08 (d, *J* = 6.7 Hz, 2H), 6.84 (dd, *J* = 8.3, 2.6 Hz, 1H), 6.65 (d, *J* = 7.6 Hz, 1H), 6.61 (t, *J* = 2.2 Hz, 1H), 5.97 (s, 1H), 3.78 (s, 3H).
^{13}C NMR (101 MHz, $CDCl_3$) δ 159.9, 149.0, 146.9, 142.6, 140.8, 135.5, 131.7, 129.7, 129.4, 128.7, 127.2, 124.9, 121.9, 121.5, 115.8, 111.9, 55.2, 53.6.
IR: 2920, 2851, 1735, 1598, 1519, 1493, 1454, 1376, 1348, 1136, 1075, 1046, 893 cm^{-1}.
HRMS ($^+$APCI) *m/z*: calcd for $C_{20}H_{17}ClNO_3$ $[M+H]^+$ 354.0897 found 354.0892.

$[\alpha]^{23}_{D}$ 4.2 (*c* 0.99, $CHCl_3$)
HPLC: (ODH column, 1.0 mL/min 1% *i*-PrOH in *n*-hexane 30 min, UV 230 nm) retention times of: 15.3 (major) and 18.9 min (minor) 75 % ee with Rh_2(*S*-TPPTTL)$_4$.

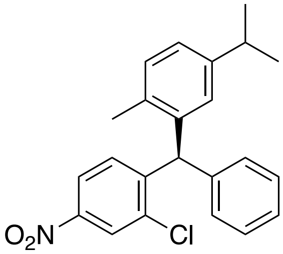

(*R*)-2-chloro-1-((5-isopropyl-2-methylphenyl)(phenyl)methyl)-4-nitrobenzene (25)

Compound 25 was obtained according to general procedure C from the C–H functionalization reaction between 1-isopropyl-4-methylcyclohexa-1,4-diene (0.19 mL, 1.2 mmol, 4 equiv) and 2-chloro-1-(diazo(phenyl)methyl)-4-nitrobenzene (82mg, 0.3 mmol, 1.0 equiv), catalyzed by Rh_2(*S*-TPPTTL)4 (4.93 mg, 0.003 mmol, 1.0 mol %). The product was purified by flash column chromatography on silica gel (gradient elution: 0 – 5% diethyl ether in pentane) to afford an oil in 28% yield (32 mg).
^{1}H NMR (400 MHz, $CDCl_3$) δ 8.30 (d, *J* = 2.3 Hz, 1H), 8.05 (dd, *J* = 8.6, 2.37 Hz, 1H), 7.37 – 7.24 (m, 3H), 7.16 (d, *J* = 7.8 Hz, 1H), 7.13 – 7.05 (m, 2H), 7.06 – 6.99 (m, 2H), 6.54 (d, *J* = 1.9 Hz, 1H), 6.02 (s, 1H), 2.76 (hept, *J* = 6.9 Hz, 1H), 2.16 (s, 3H), 1.14 (dd, *J* = 6.9, 1.66 Hz, 6H)
^{13}C NMR (101 MHz, $CDCl_3$) δ 149.2, 146.7, 140.4, 139.3, 135.5, 134.0, 131.5, 130.7, 129.59, 129.56, 128.7, 127.2, 127.1, 124.9, 124.8, 121.5, 50.9, 33.6, 24.1, 24.0, 19.2.
IR: 2961, 1526, 1450,1347, 1258, 1020, 861 cm^{-1}.
HRMS ($^+$APCI) *m/z*: calcd for $C_{23}H_{22}ClNO_2$ $[M+H]^+$ 380.1417 found 380.1407.
$[\alpha]^{23}_{D}$ –4.7 (*c* 1.00, $CHCl_3$)
HPLC (ODH column, 1.5 mL/min 0% *i*-PrOH in *n*-hexane 60 min, UV 230 nm) retention times of 17.34 (minor) and 21.74 min (major) 73 % ee with Rh_2(*S*-TPPTTL)$_4$.

5. ^{1}H ^{13}C and ^{19}F NMR Spectra for Characterization of Compounds.

Determination of Diastereomers of compound **11a** and **11b.**

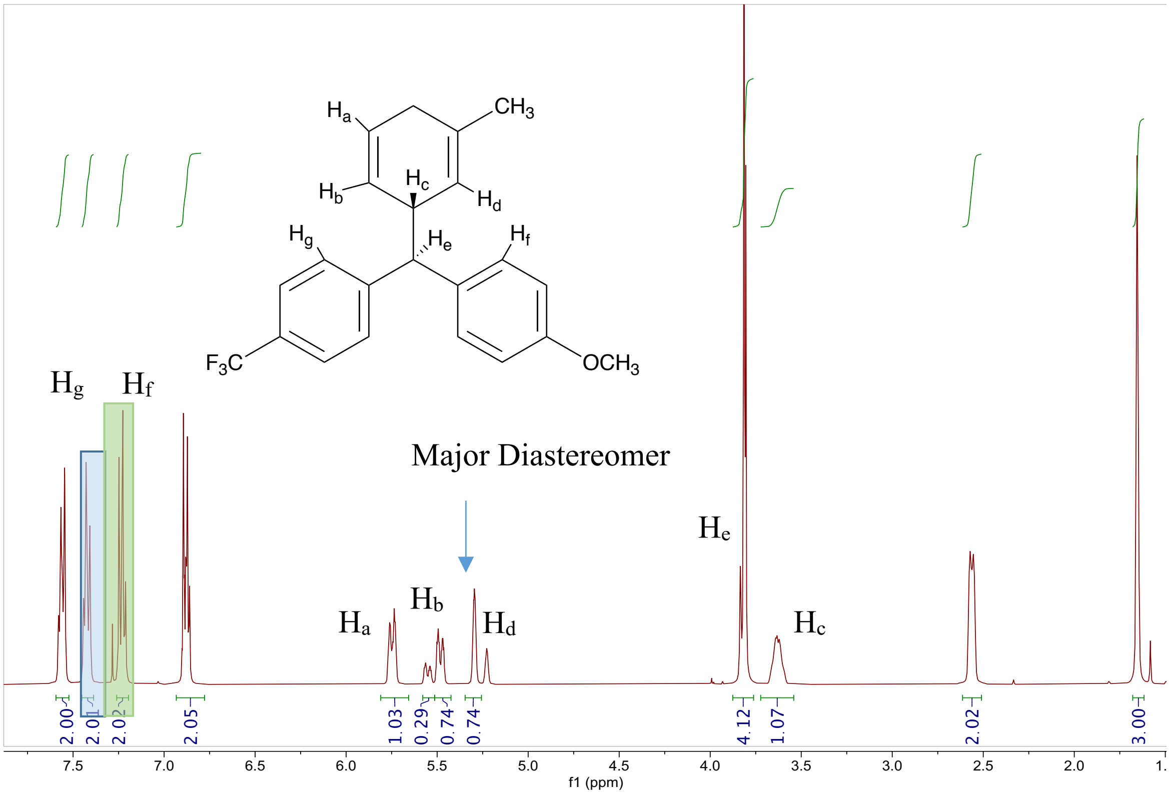

2D NOESY:

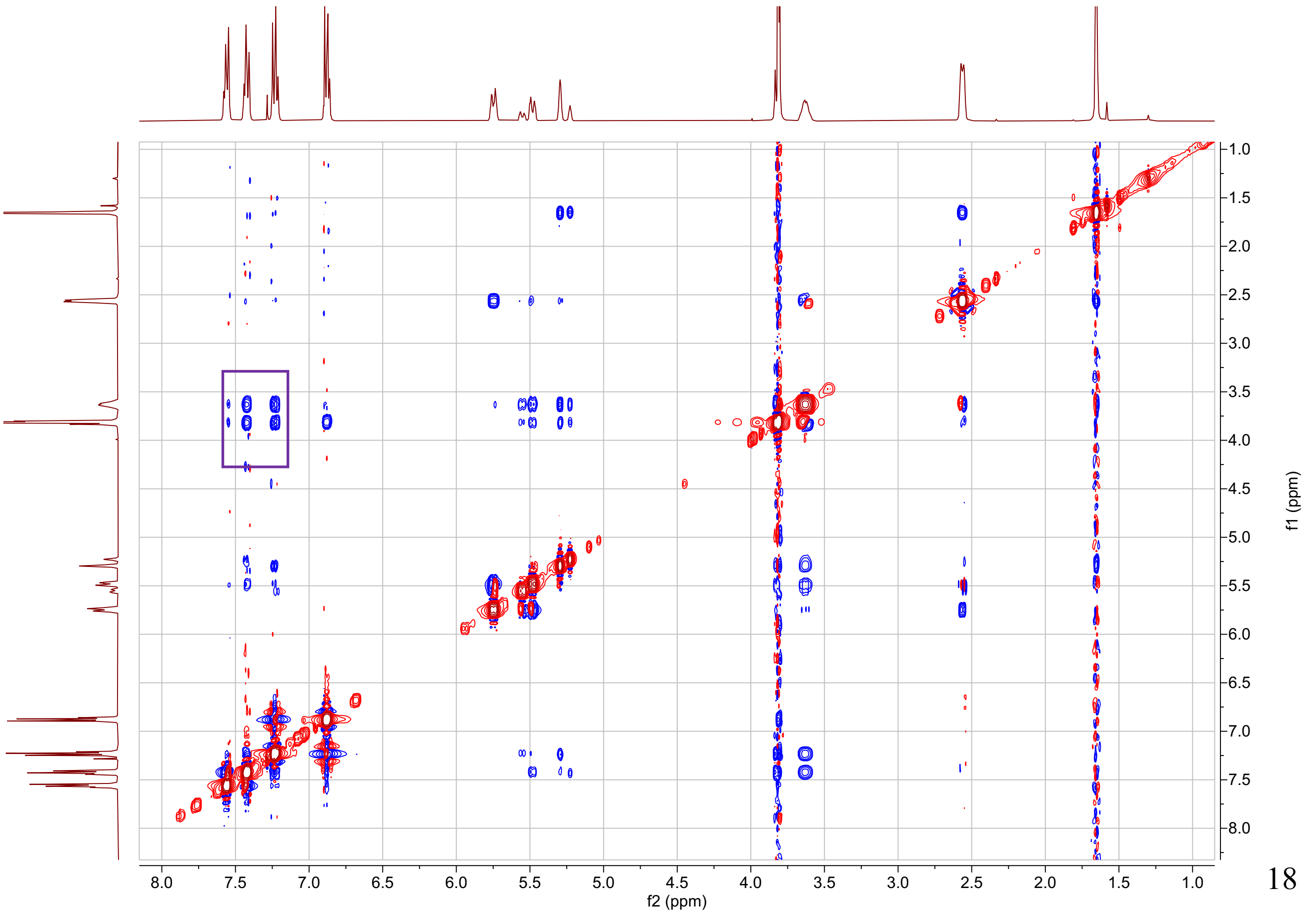

Boxed in purple is major diastereomer, showing correlation on zoomed in region of 2D NOESY.

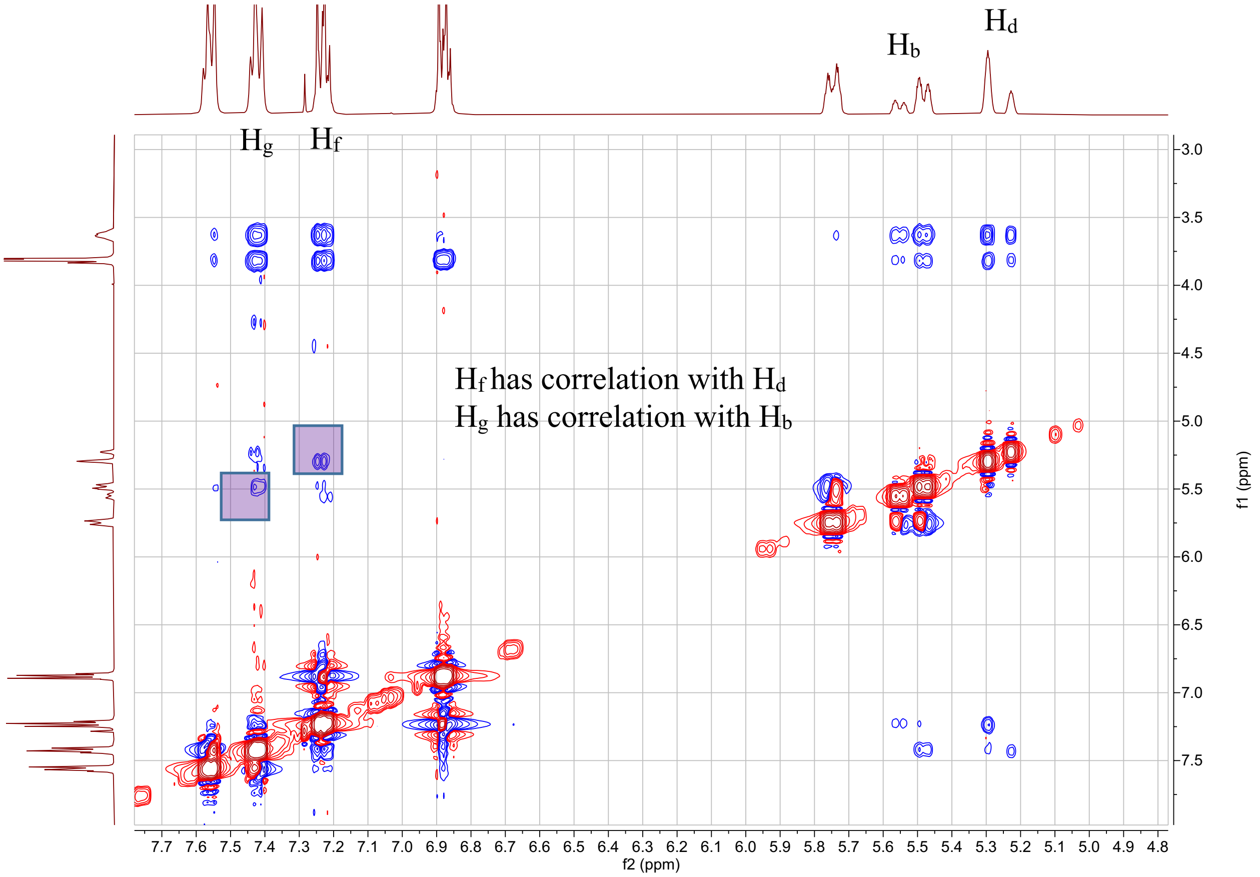

2D COSY:

Correlation between methyl group and H_d boxed in purple.

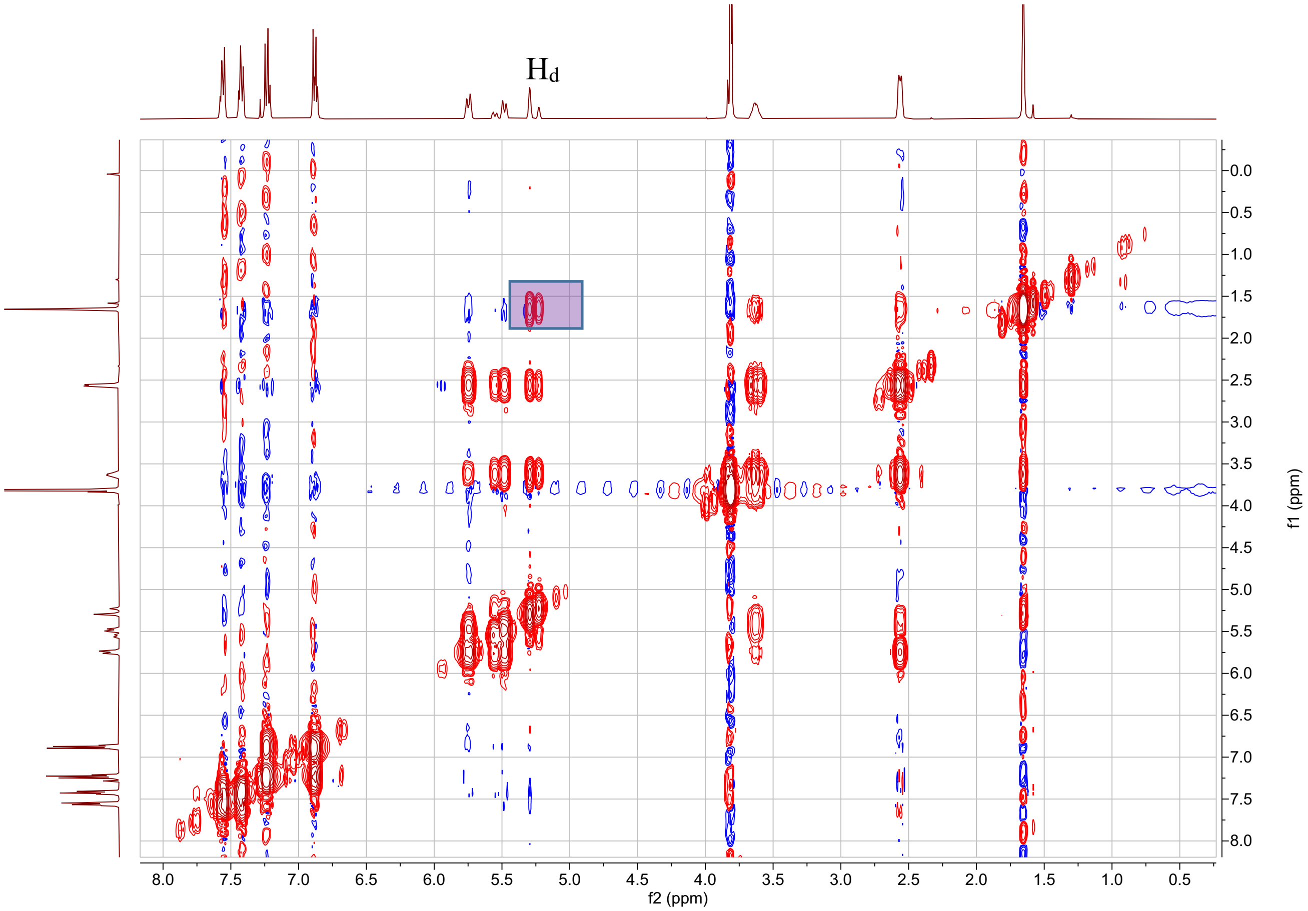

2-chloro-1-(diazo(4-methoxyphenyl)methyl)-4-nitrobenzene (SI 1)

^{1}H NMR (600 MHz, $CDCl_3$)

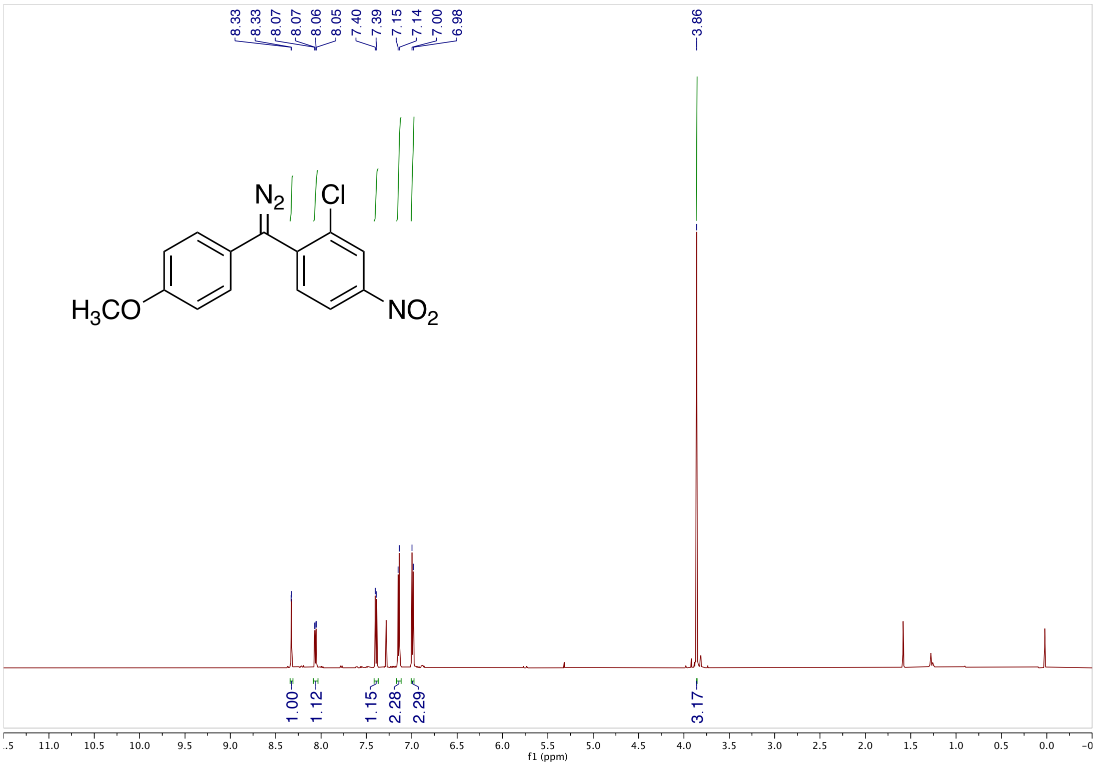

^{13}C NMR (600 MHz, $CDCl_3$)

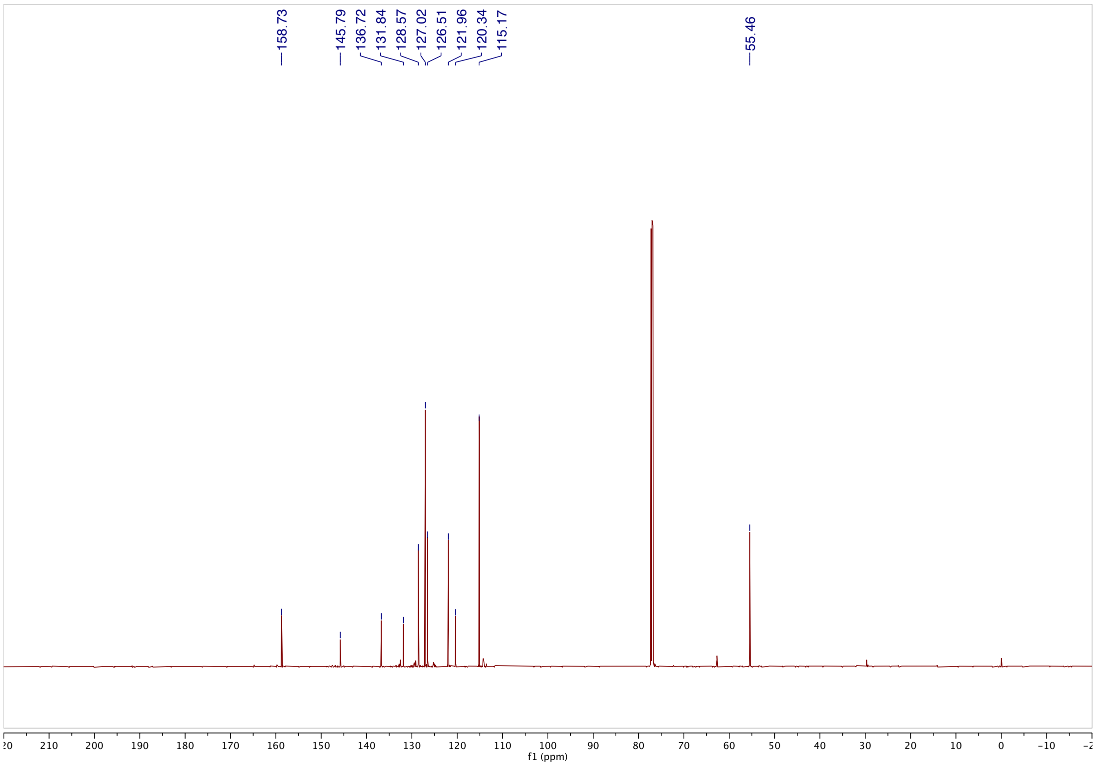

3-(diazo(4-fluorophenyl)methyl)pyridine (SI-2)

^{1}H NMR (400 MHz, $CDCl_3$)

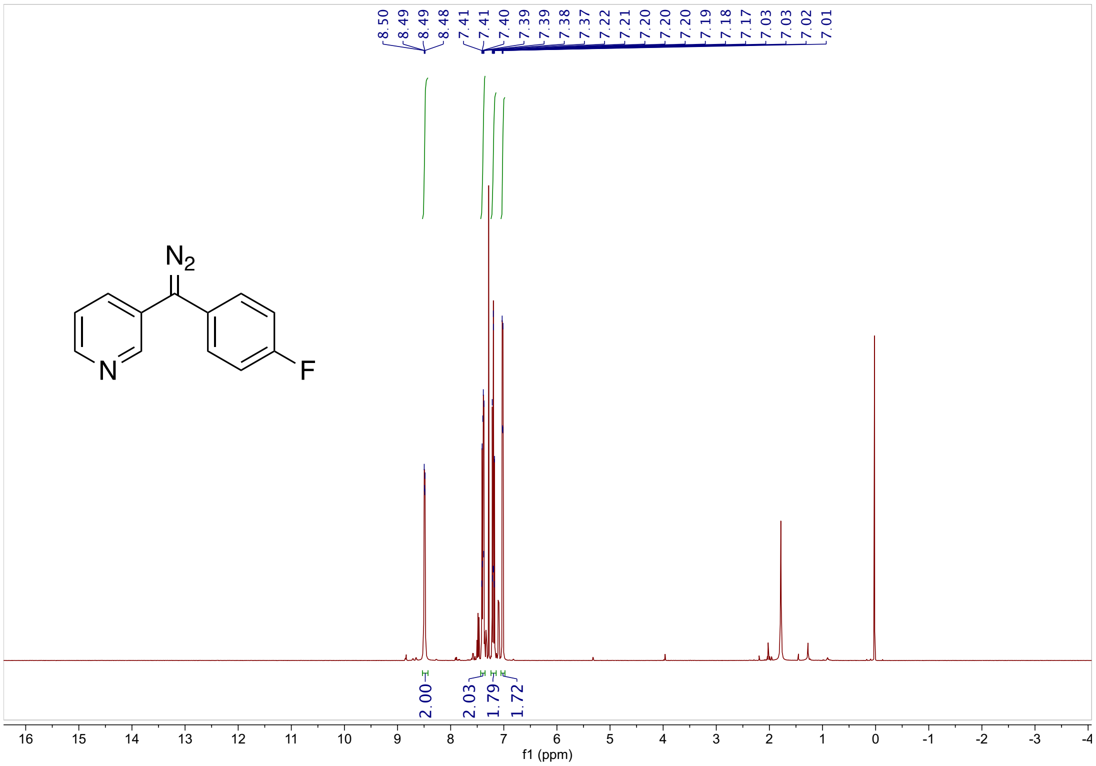

^{19}F NMR (400 MHz, $CDCl_3$)

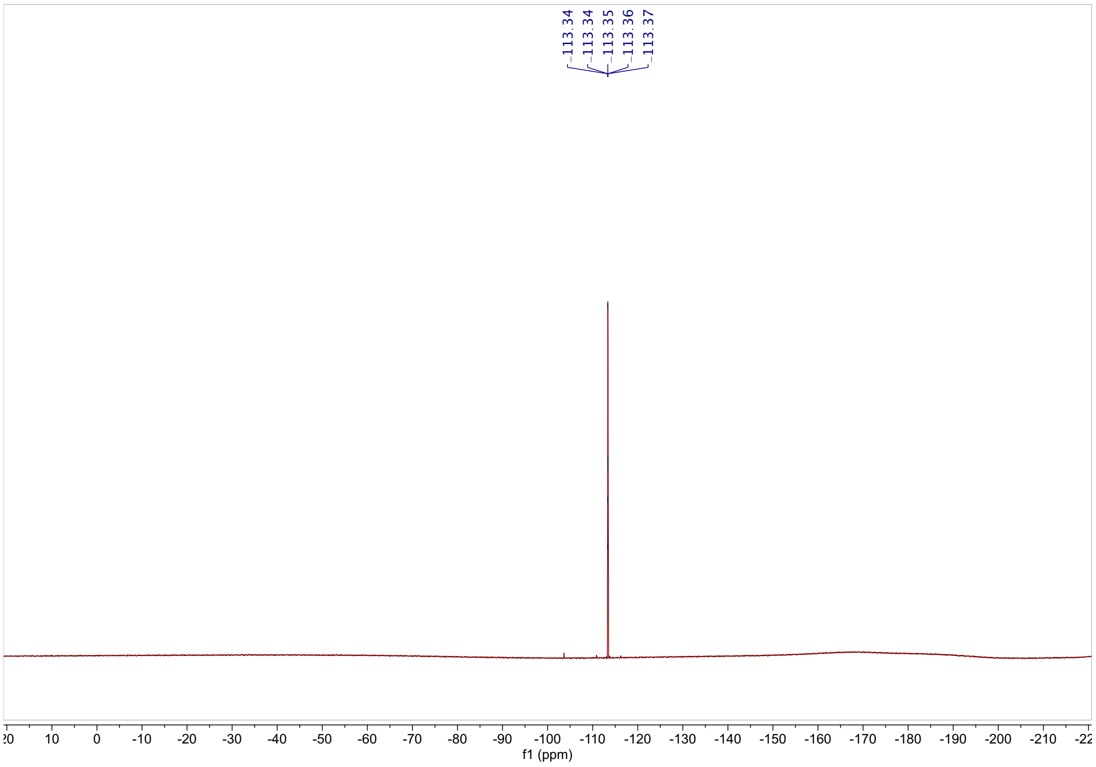

Compound 3

^{1}H NMR (400 MHz, $CDCl_3$)

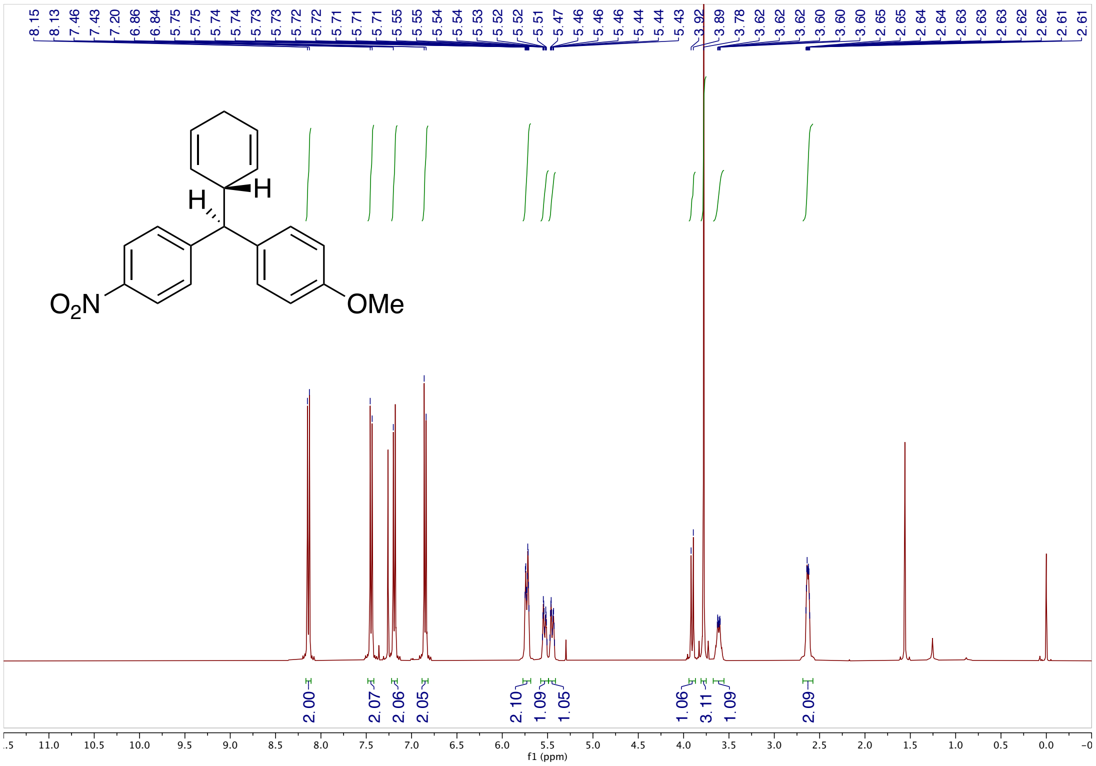

^{13}C NMR (400 MHz, $CDCl_3$)

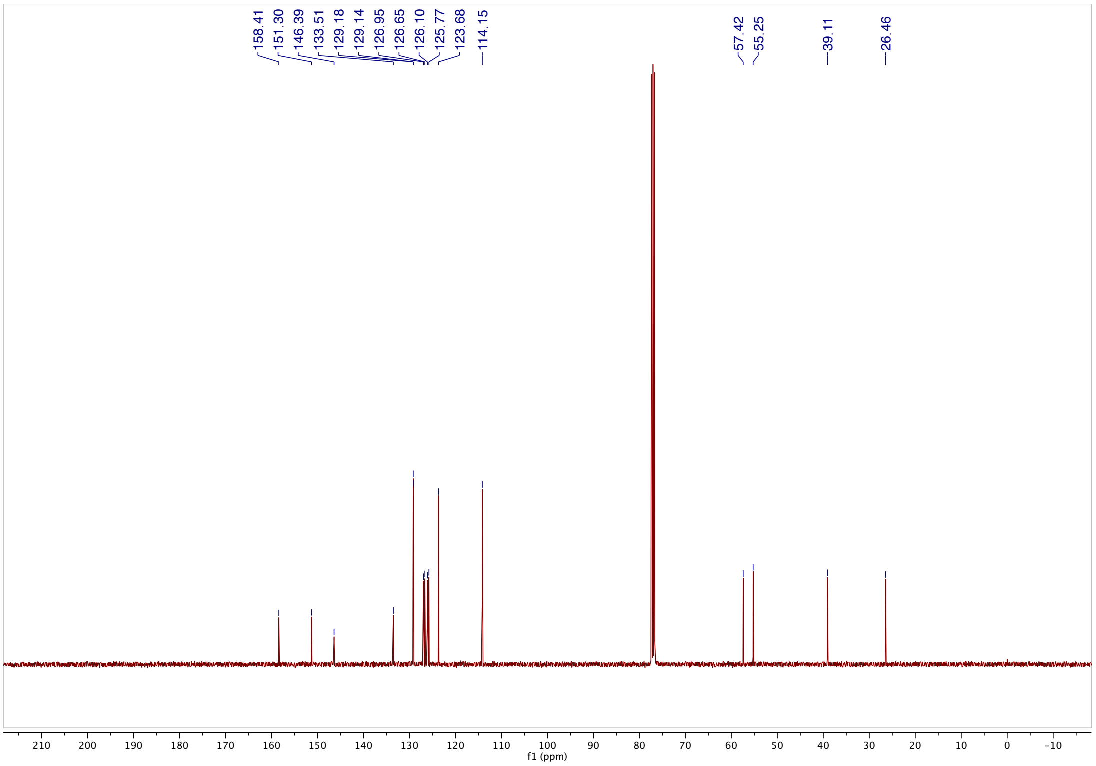

Compound 4
^{1}H NMR (400 MHz, $CDCl_3$)

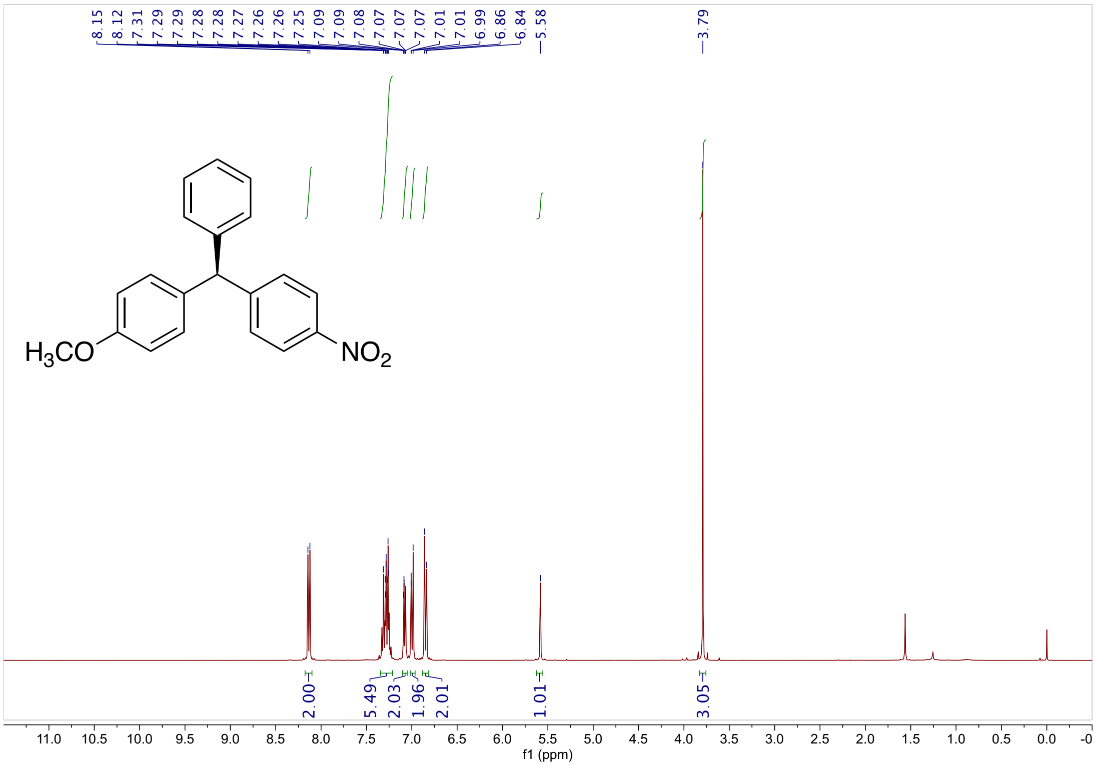

^{13}C NMR (400 MHz, $CDCl_3$)

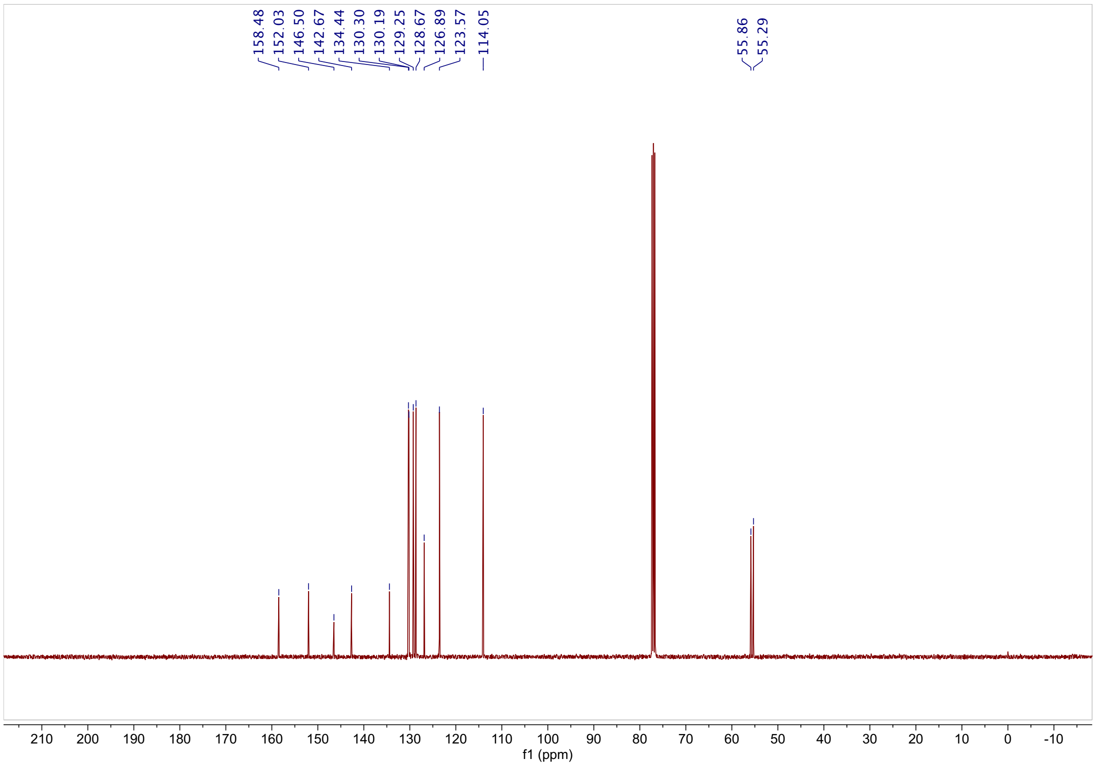

Compound 5

¹H NMR (600 MHz, $CDCl_3$)

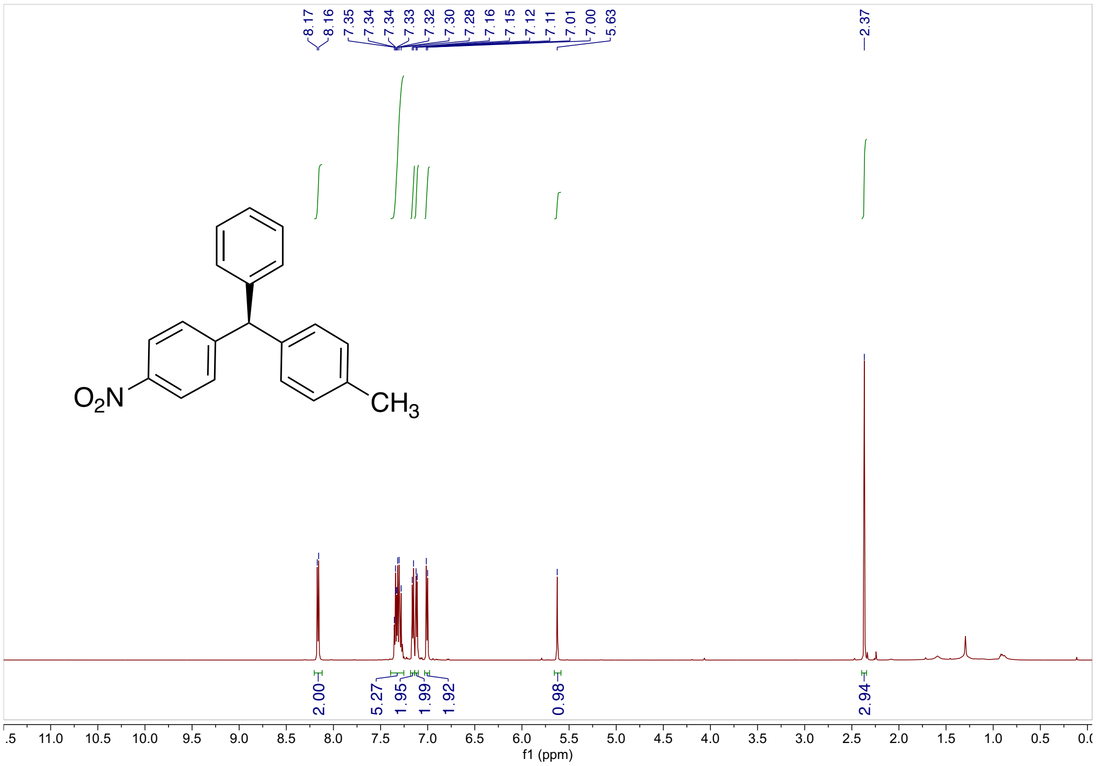

¹³C NMR (600 MHz, $CDCl_3$)

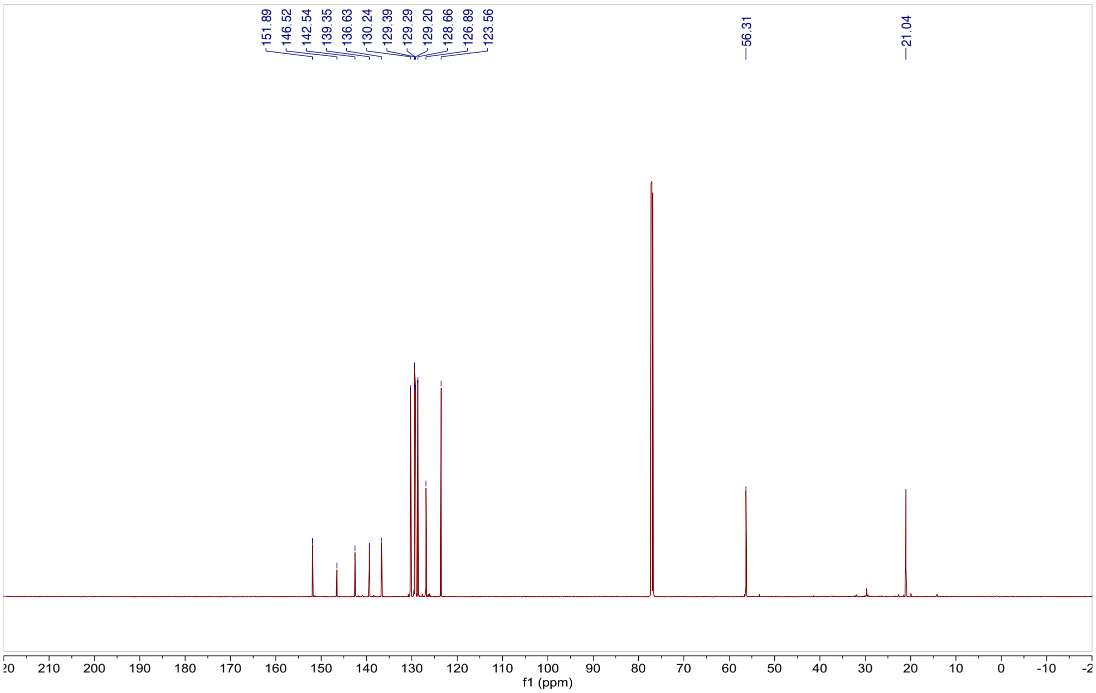

Compound 7

^{1}H NMR (600 MHz, $CDCl_3$)

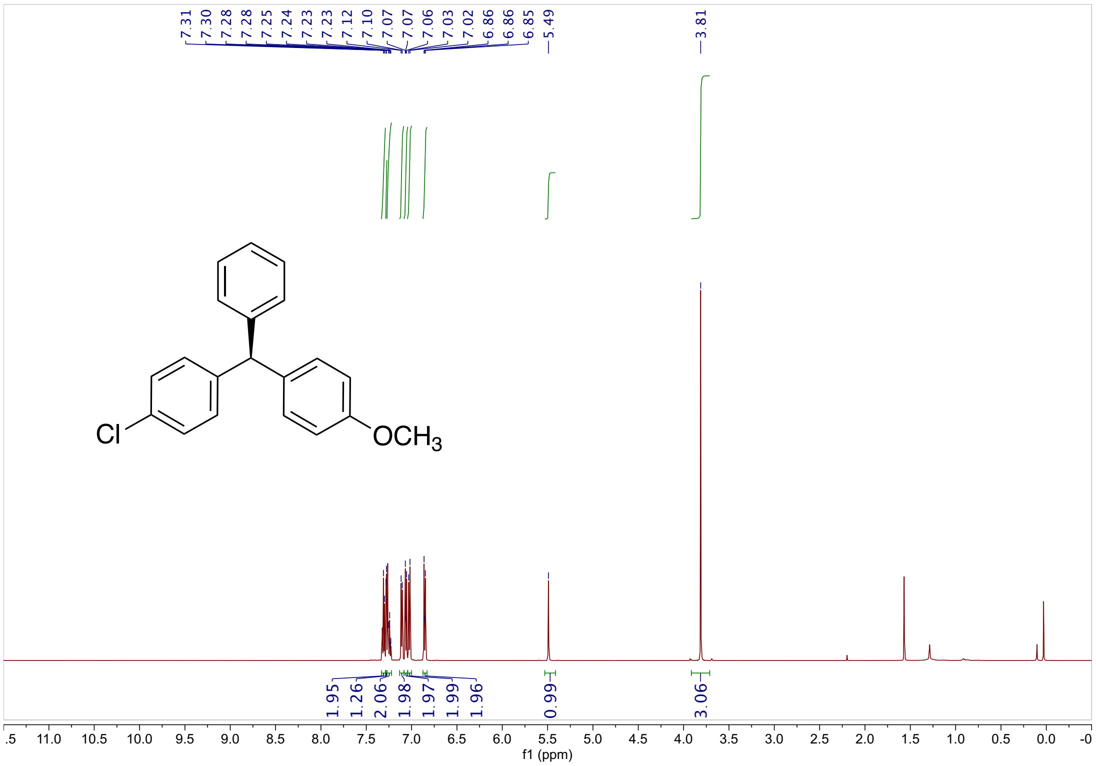

^{13}C NMR (600 MHz, $CDCl_3$)

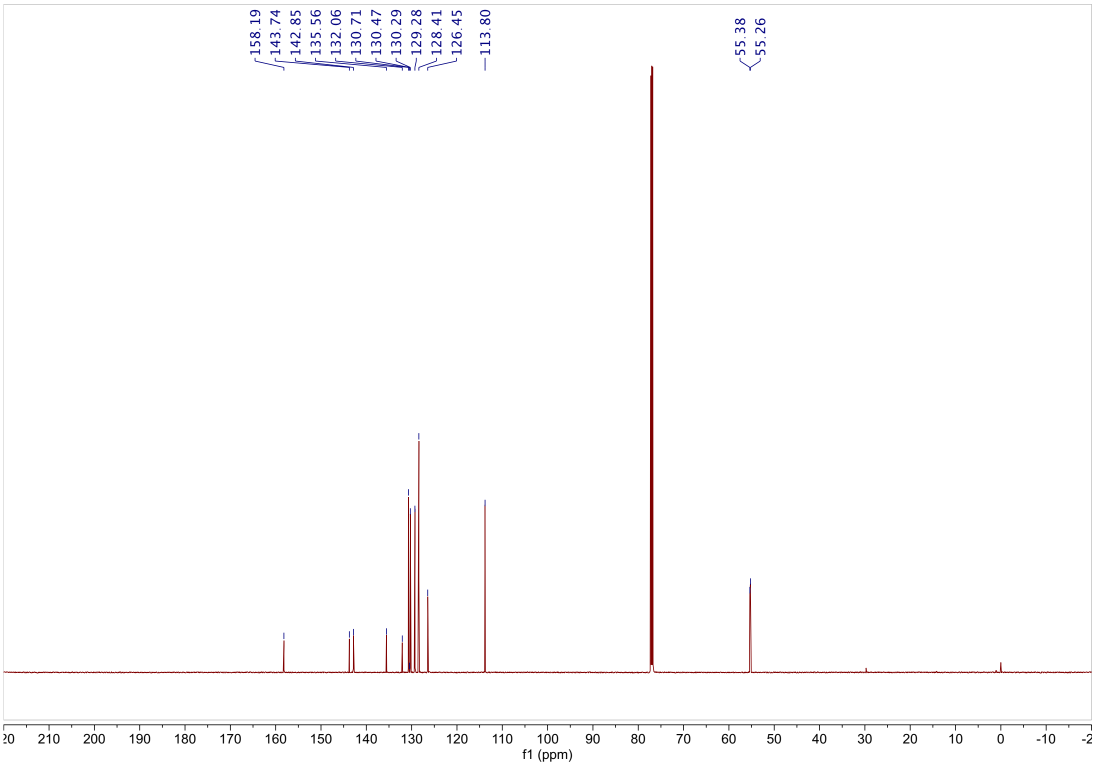

Compound 8

^{1}H NMR (600 MHz, $CDCl_3$)

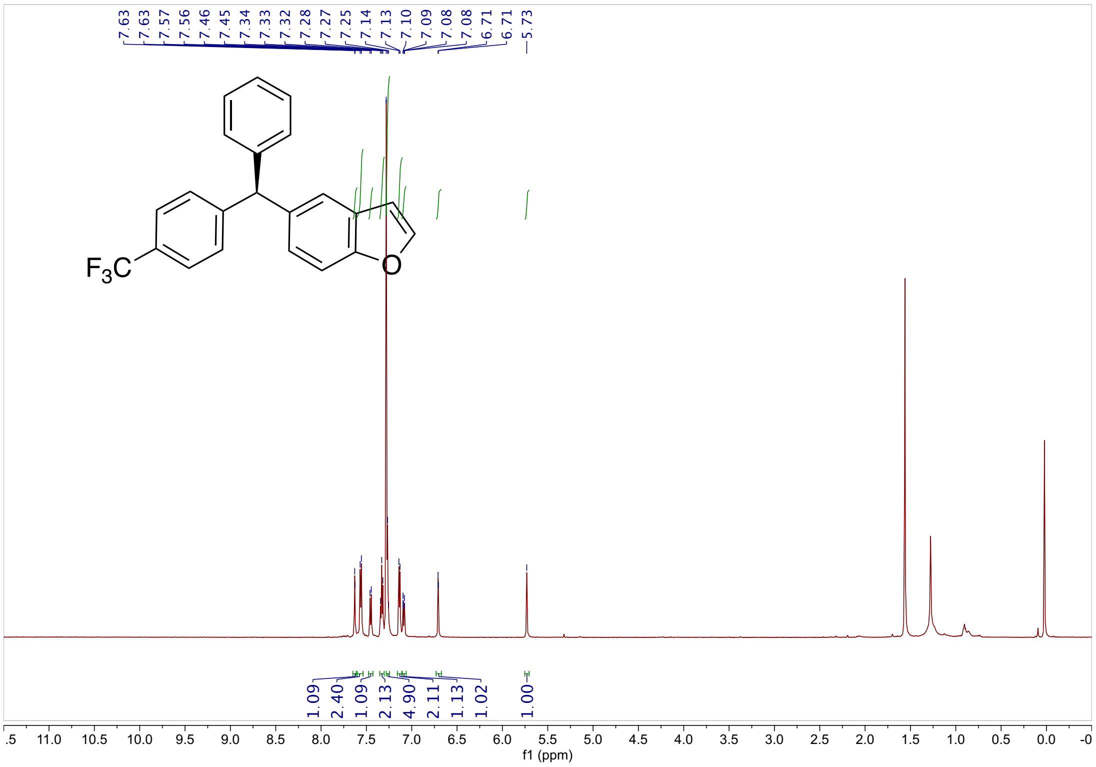

^{13}C NMR (600 MHz, $CDCl_3$)

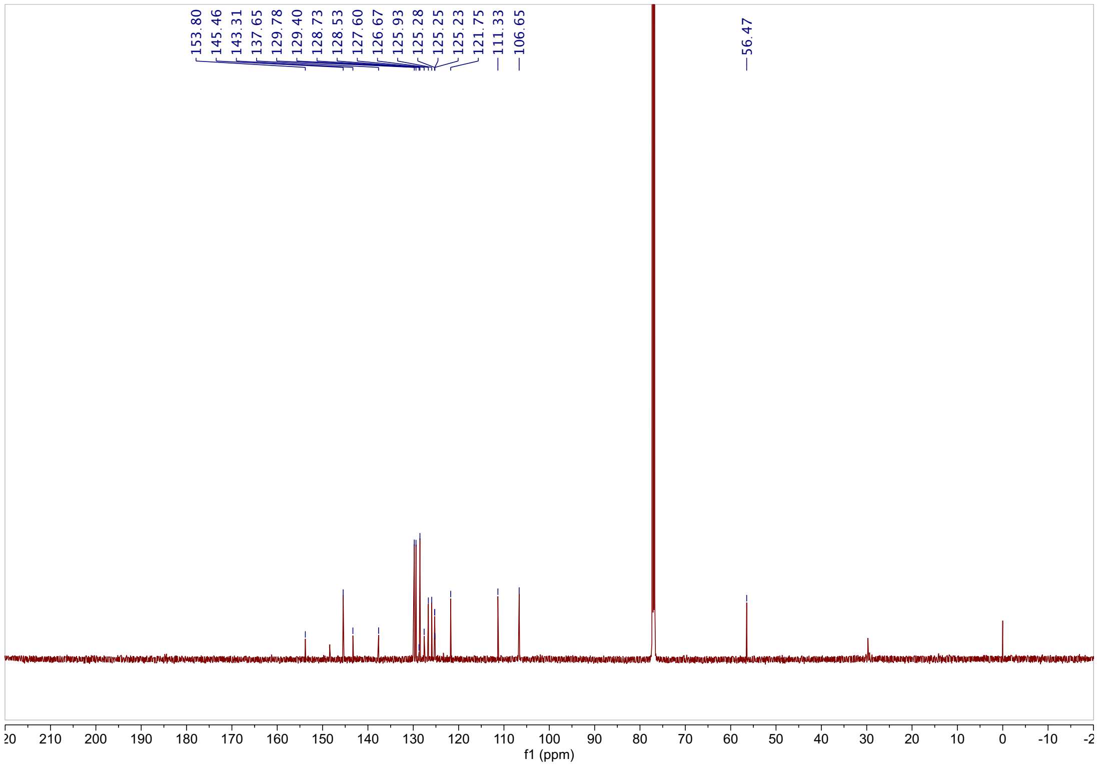

^{19}F NMR (600 MHz, $CDCl_3$) of compound **8**.

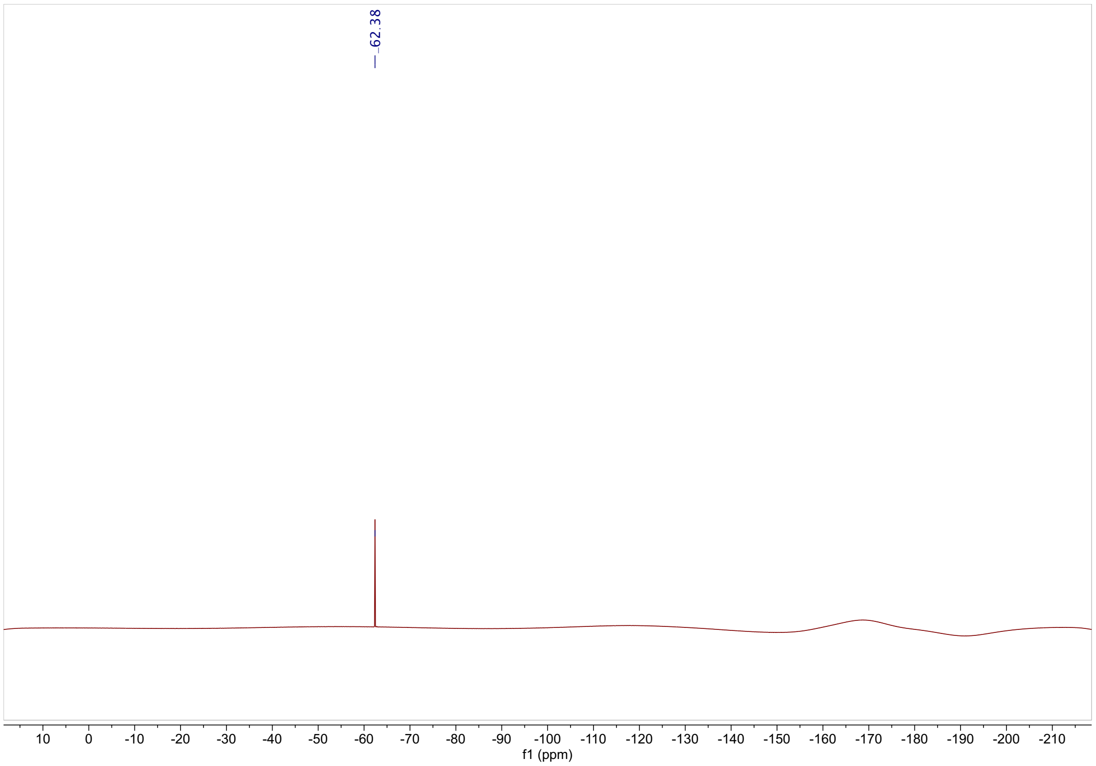

Compound 9
^{1}H NMR (600 MHz, $CDCl_3$)

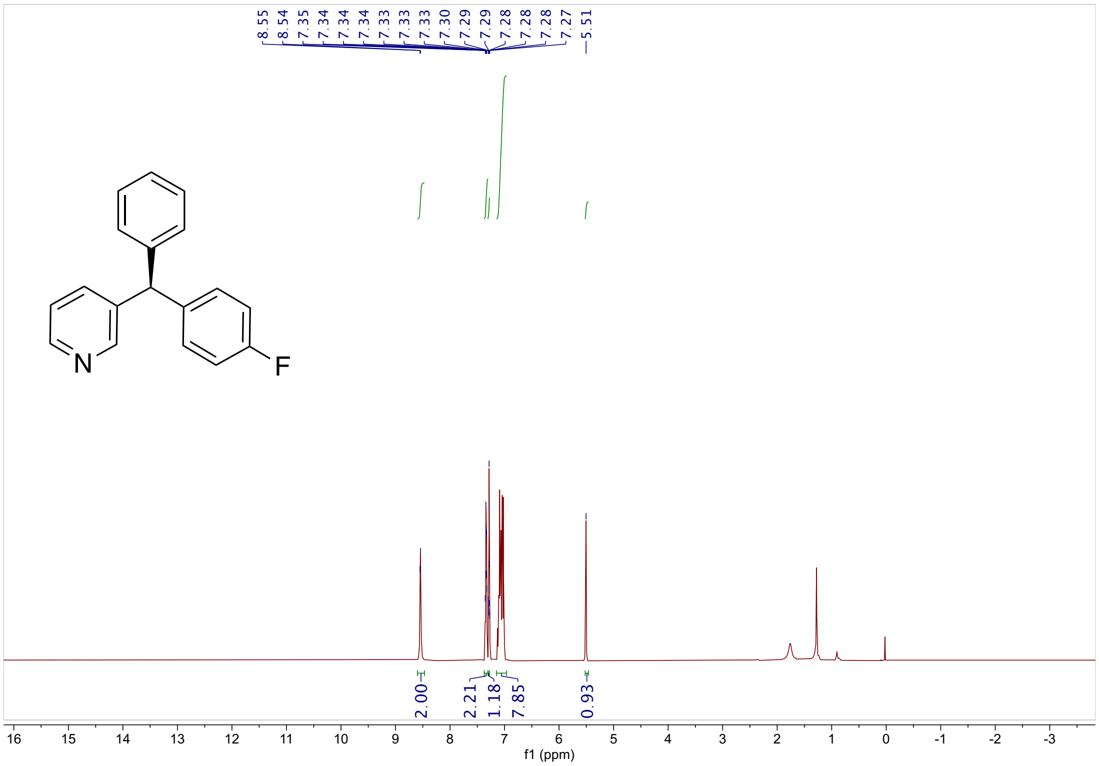

^{13}C NMR (600 MHz, $CDCl_3$)

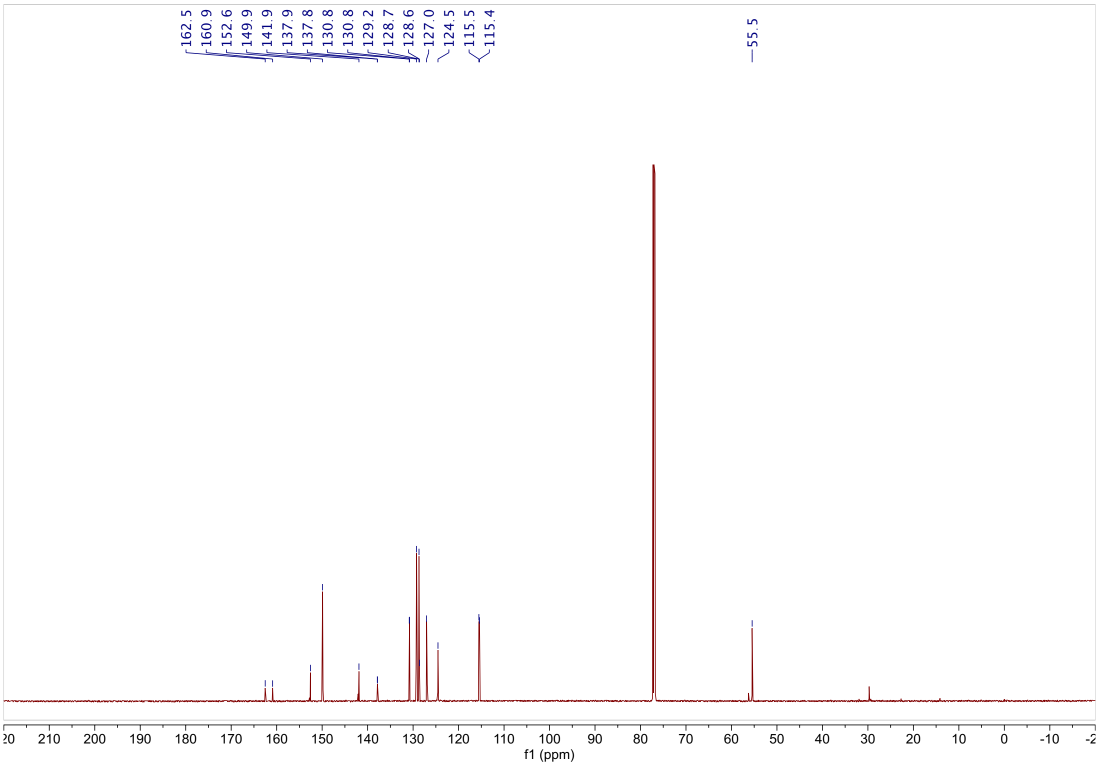

^{19}**F NMR** (600 MHz, $CDCl_3$) of compound **9**.

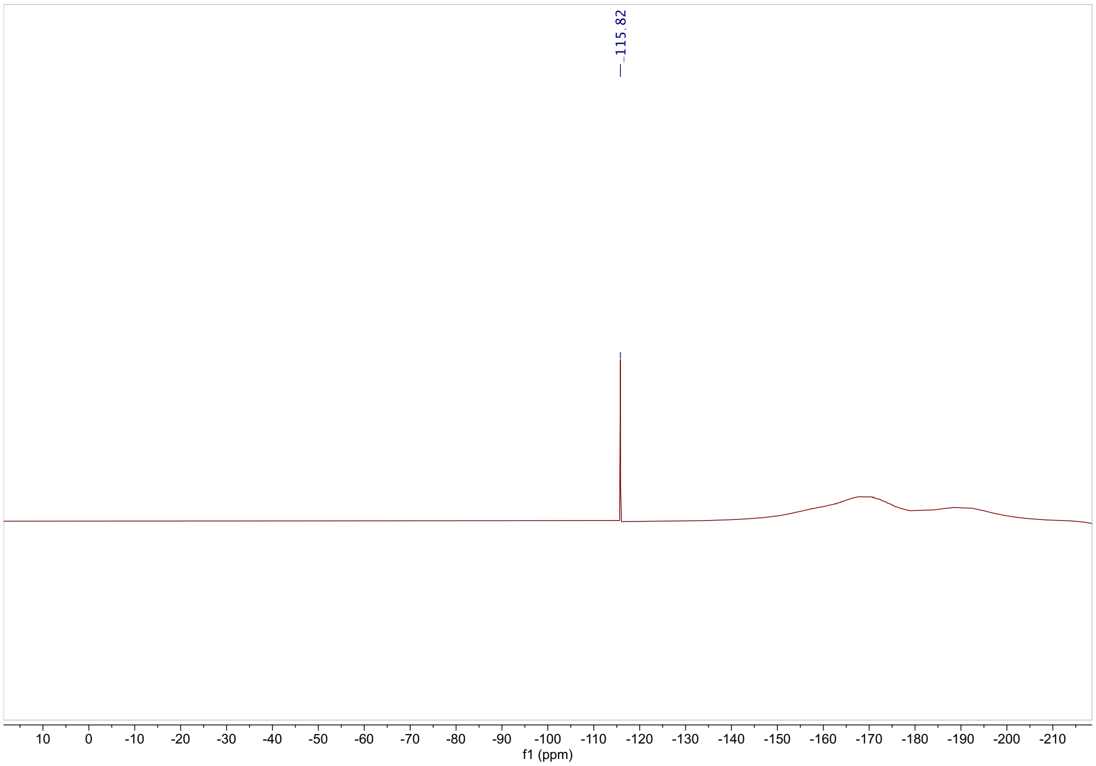

Compound 12

^{1}H NMR (600 MHz, $CDCl_3$)

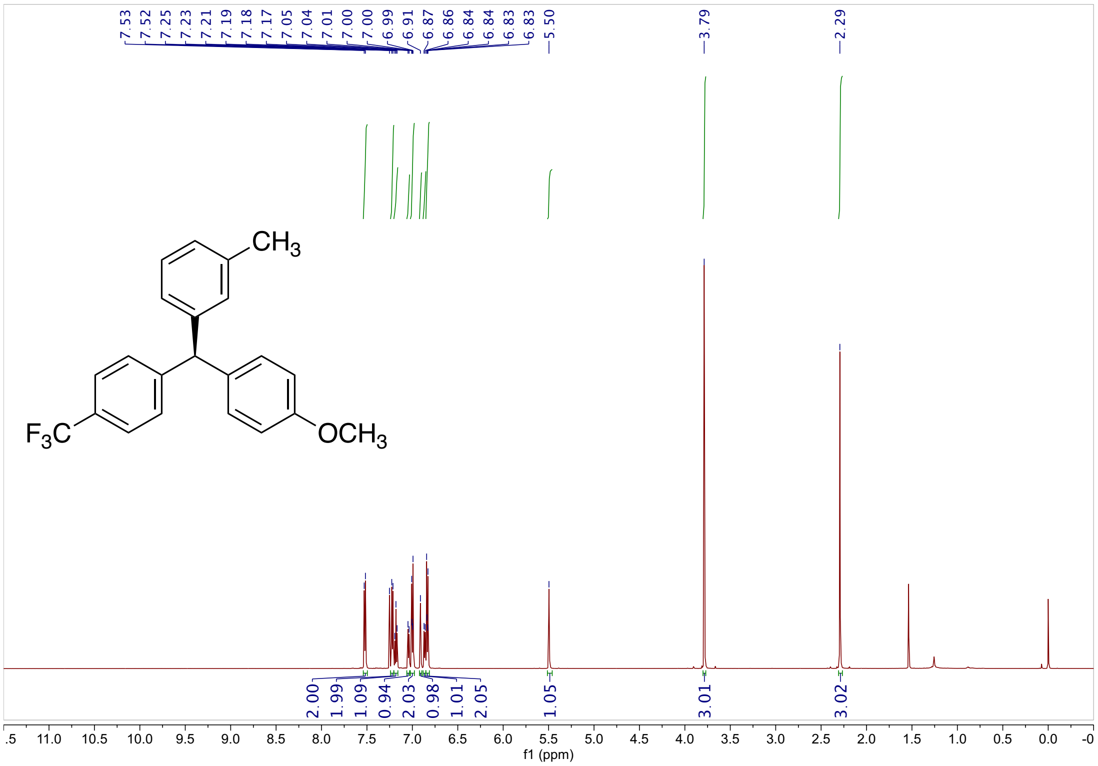

^{13}C NMR (600 MHz, $CDCl_3$)

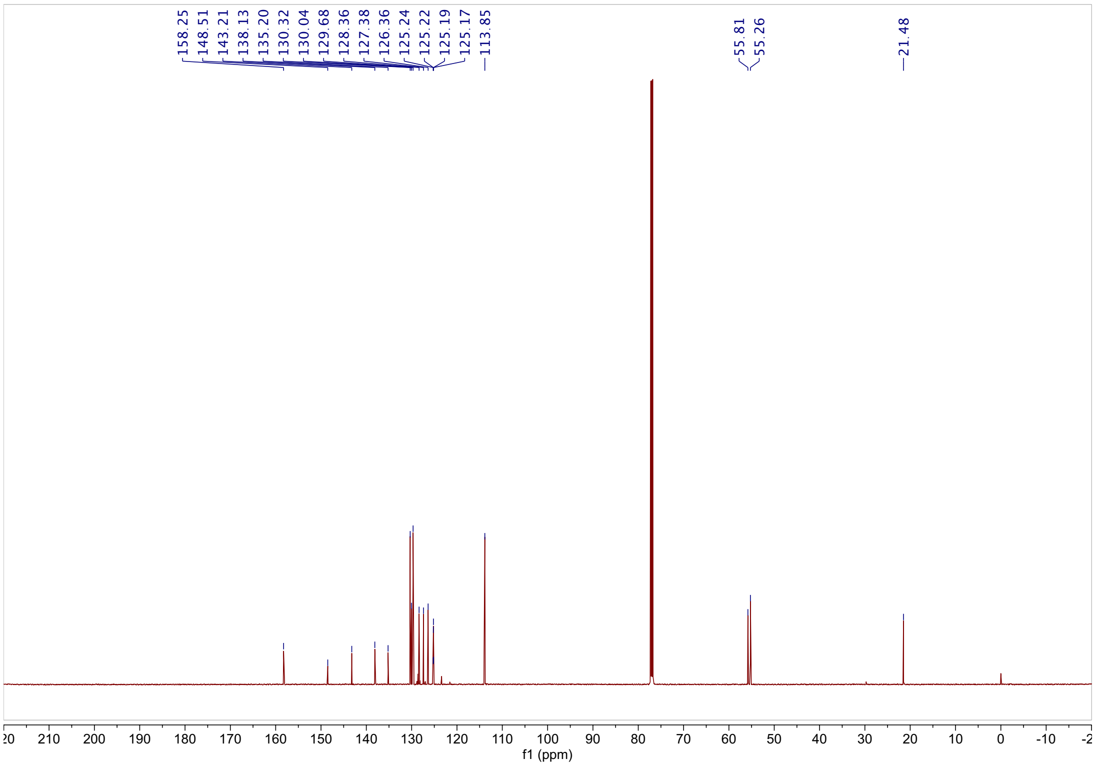

^{19}F NMR (600 MHz, $CDCl_3$) of compound **12**.

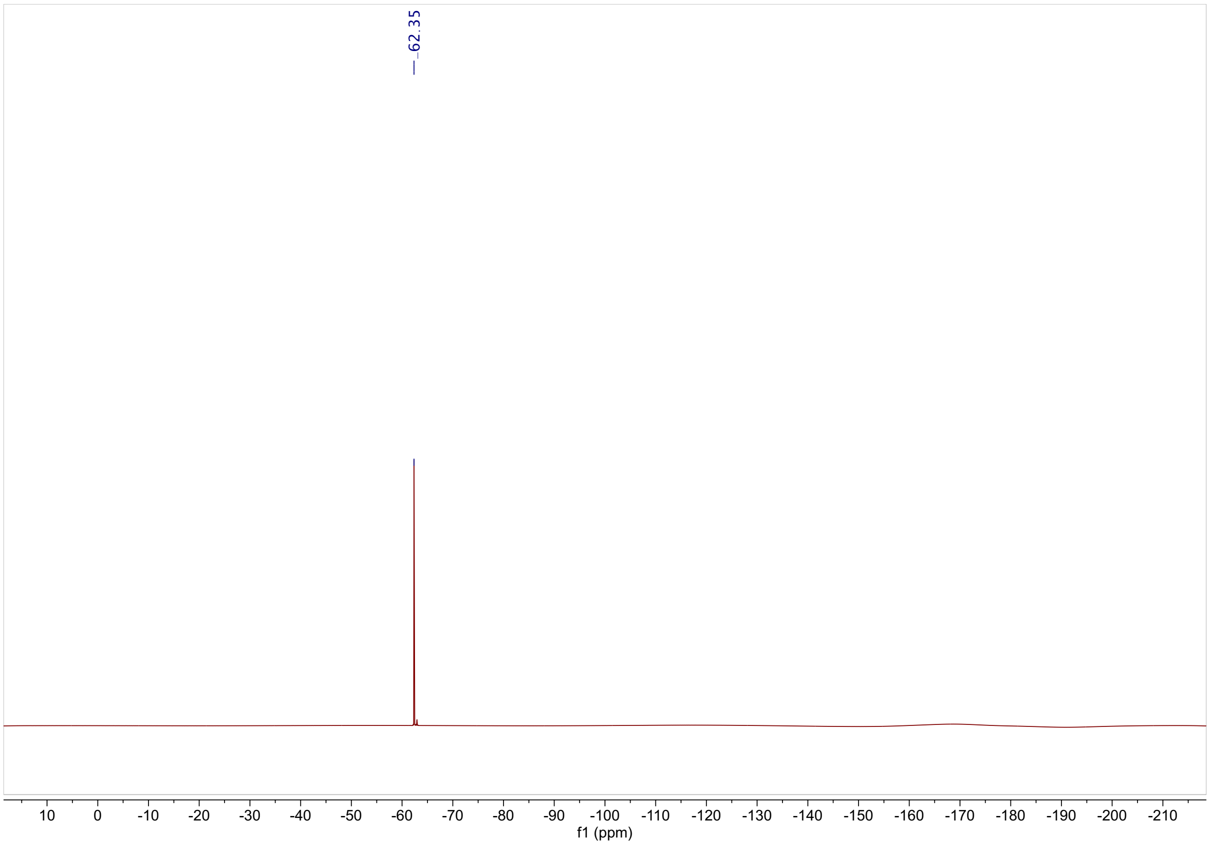

Compound 13

^{1}**H NMR** (600 MHz, $CDCl_3$)

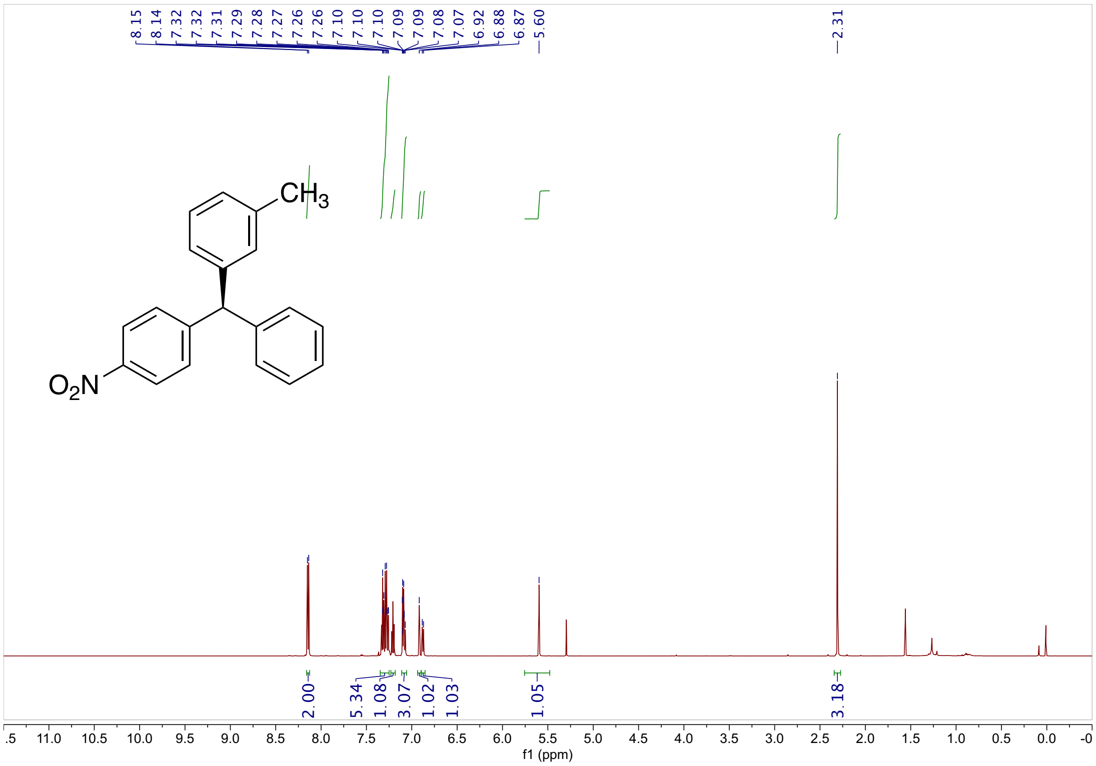

^{13}**C NMR** (600 MHz, $CDCl_3$)

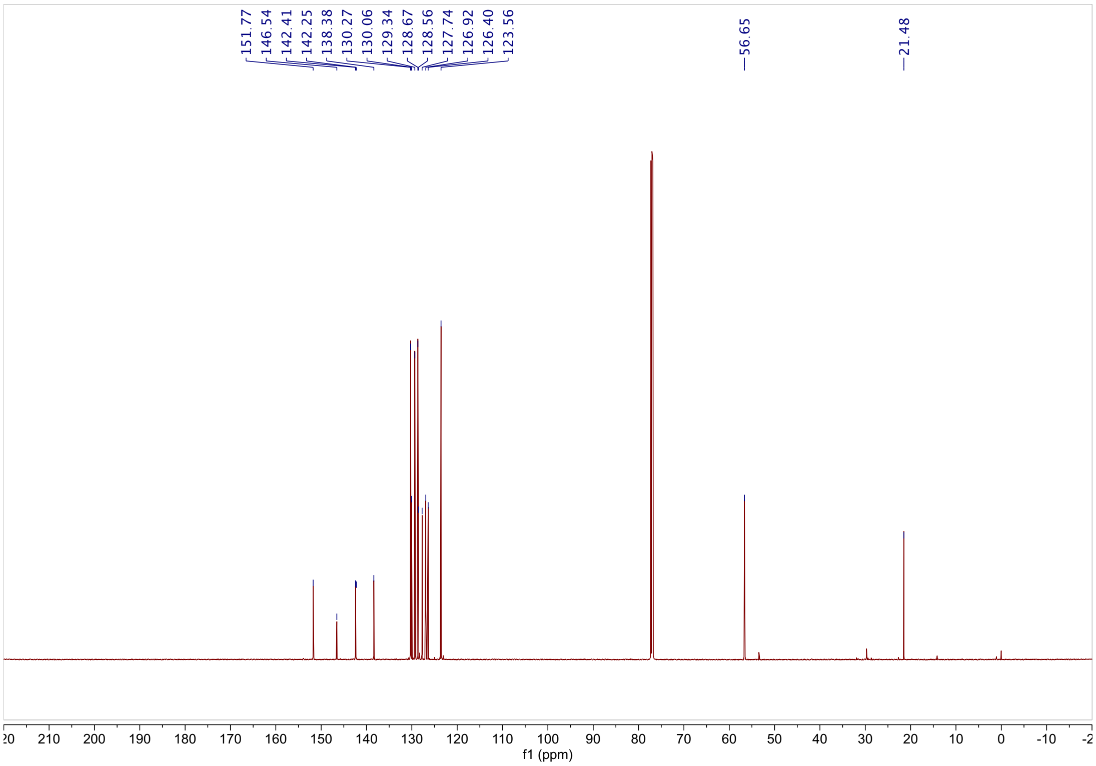

Compound 14

^{1}**H NMR** (600 MHz, $CDCl_3$)

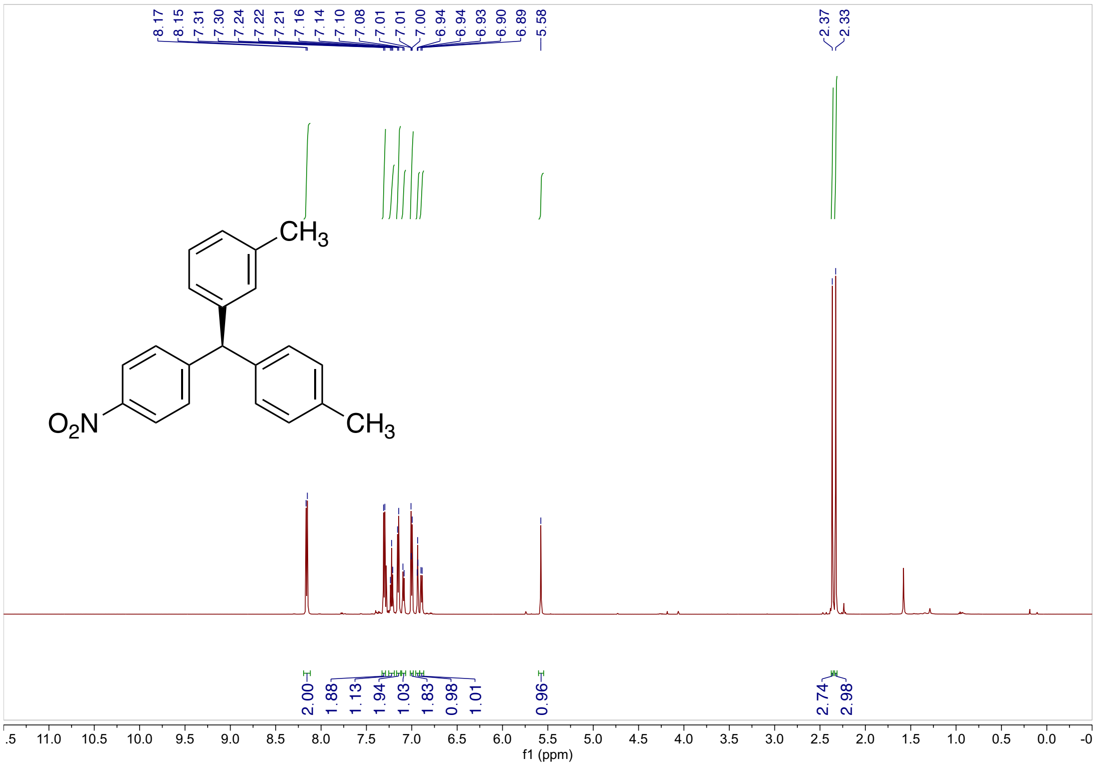

^{13}**C NMR** (600 MHz, $CDCl_3$)

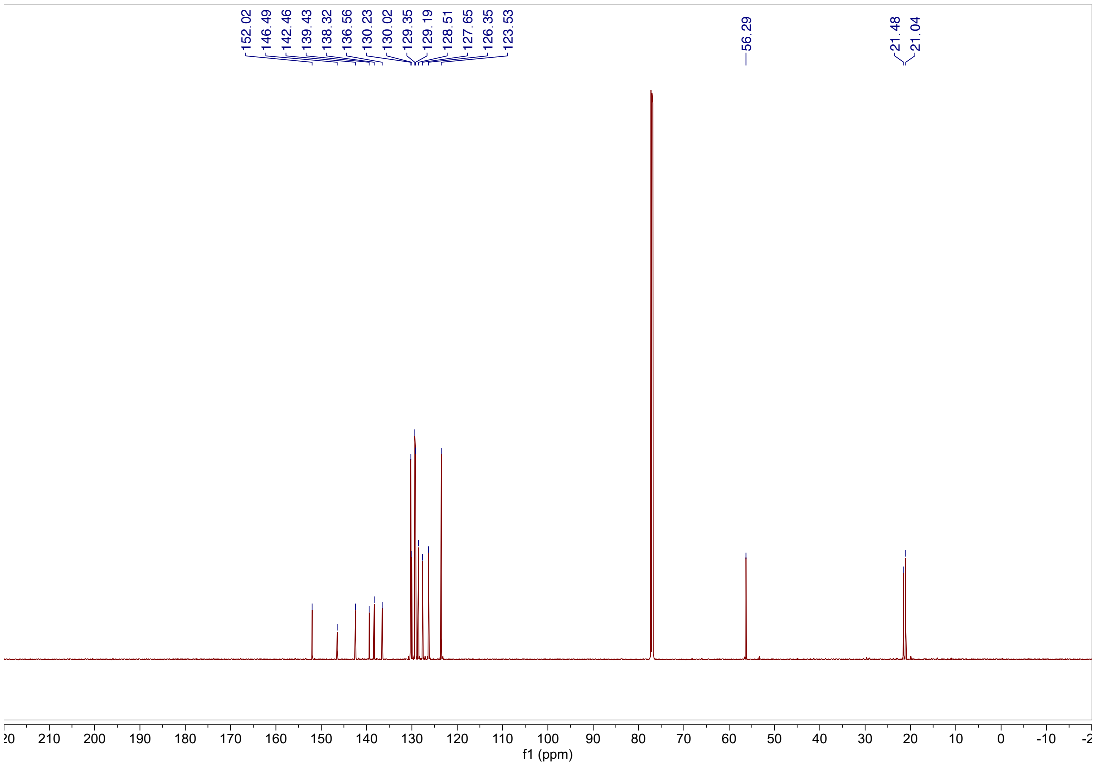

Compound 15

^{1}H NMR (600 MHz, $CDCl_3$)

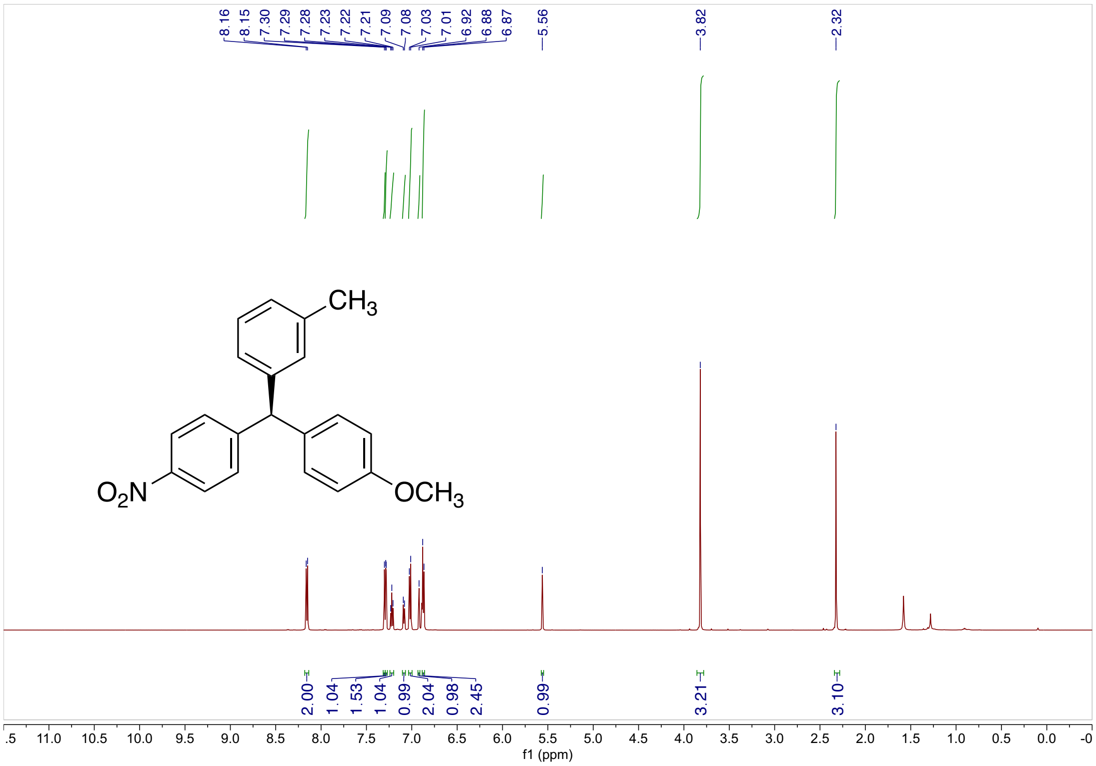

^{13}C NMR (600 MHz, $CDCl_3$)

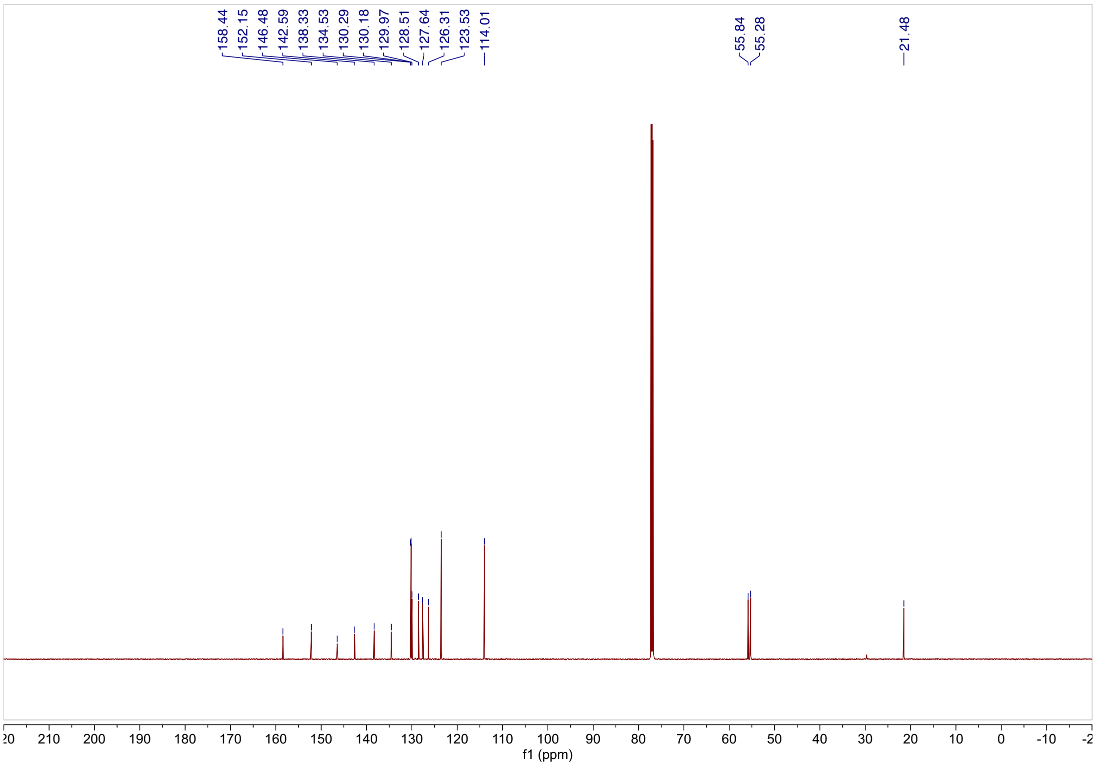

Compound 17

^{1}H NMR (600 MHz, $CDCl_3$)

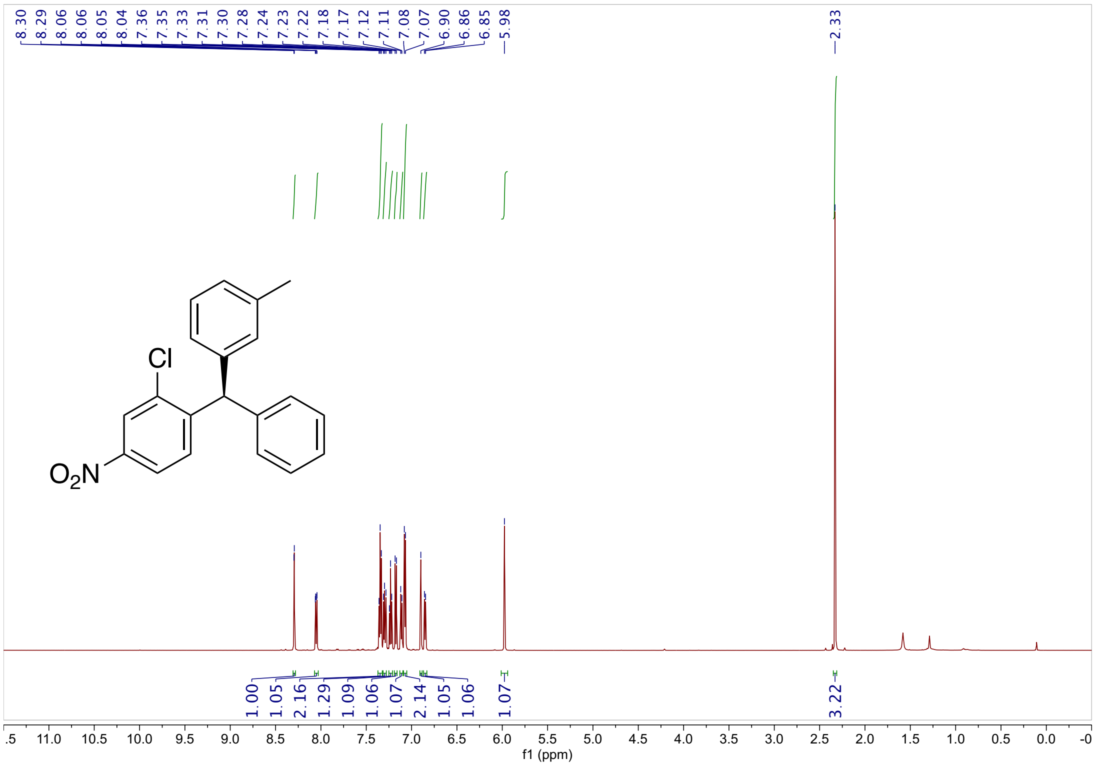

^{13}C NMR (600 MHz, $CDCl_3$)

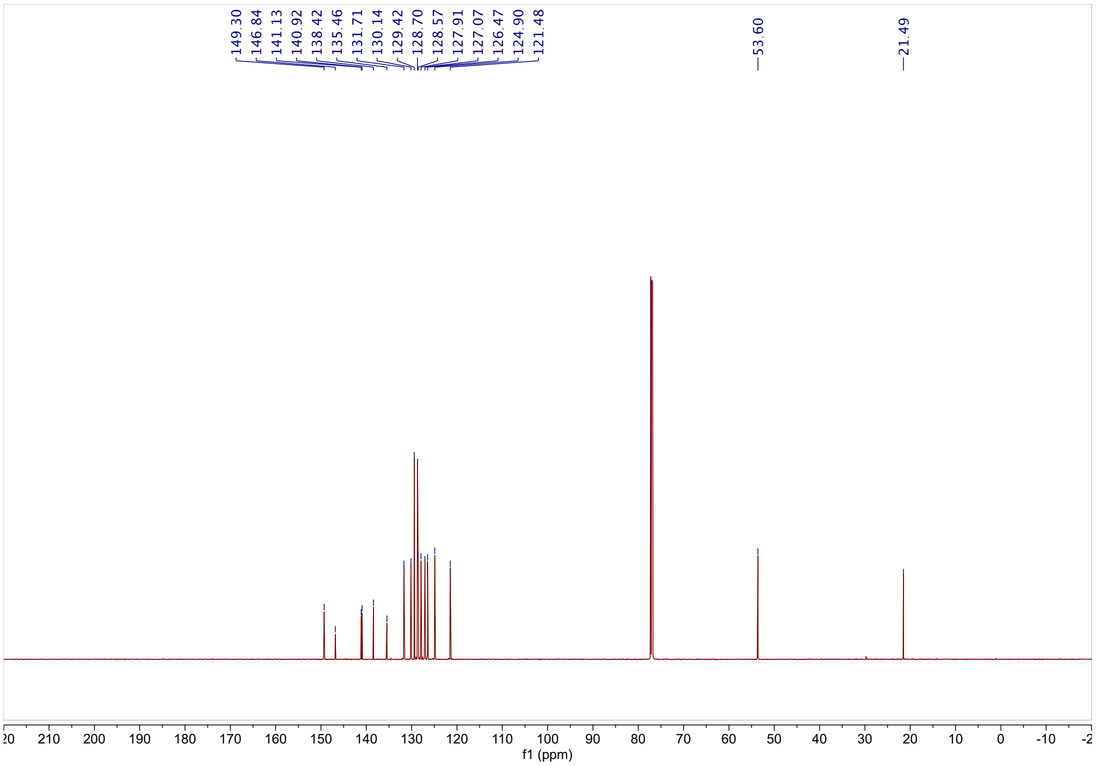

Compound 18

^{1}H NMR (600 MHz, $CDCl_3$)

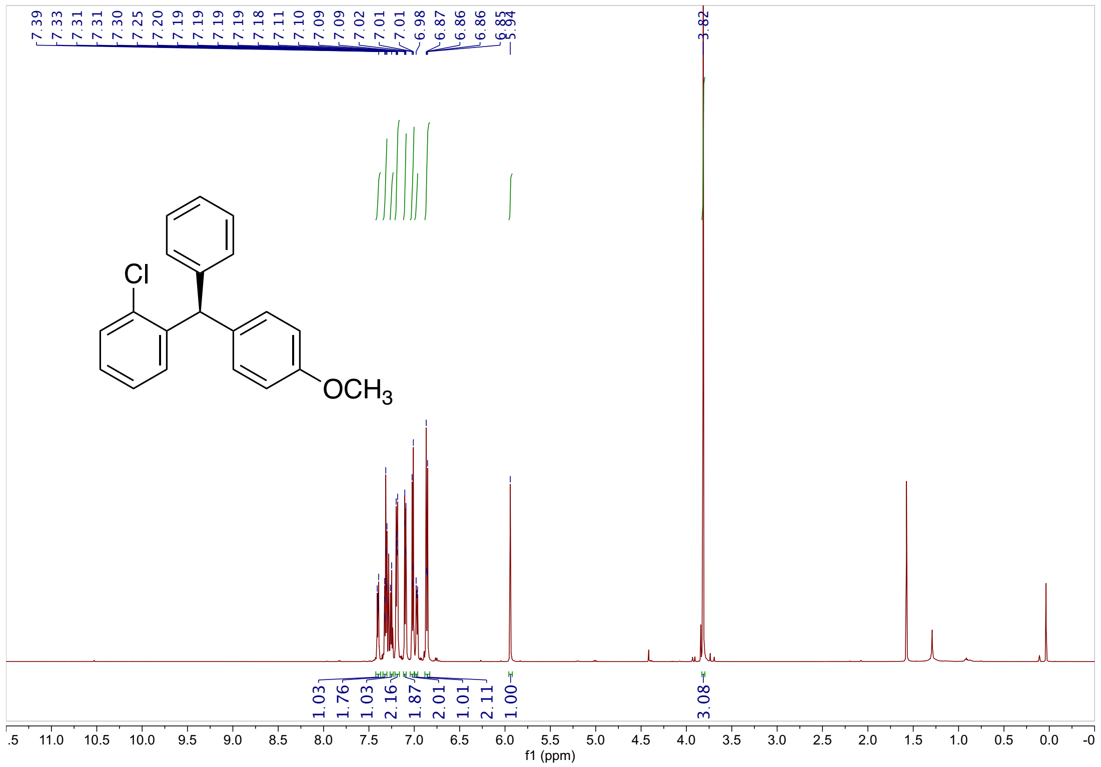

^{13}C NMR (600 MHz, $CDCl_3$)

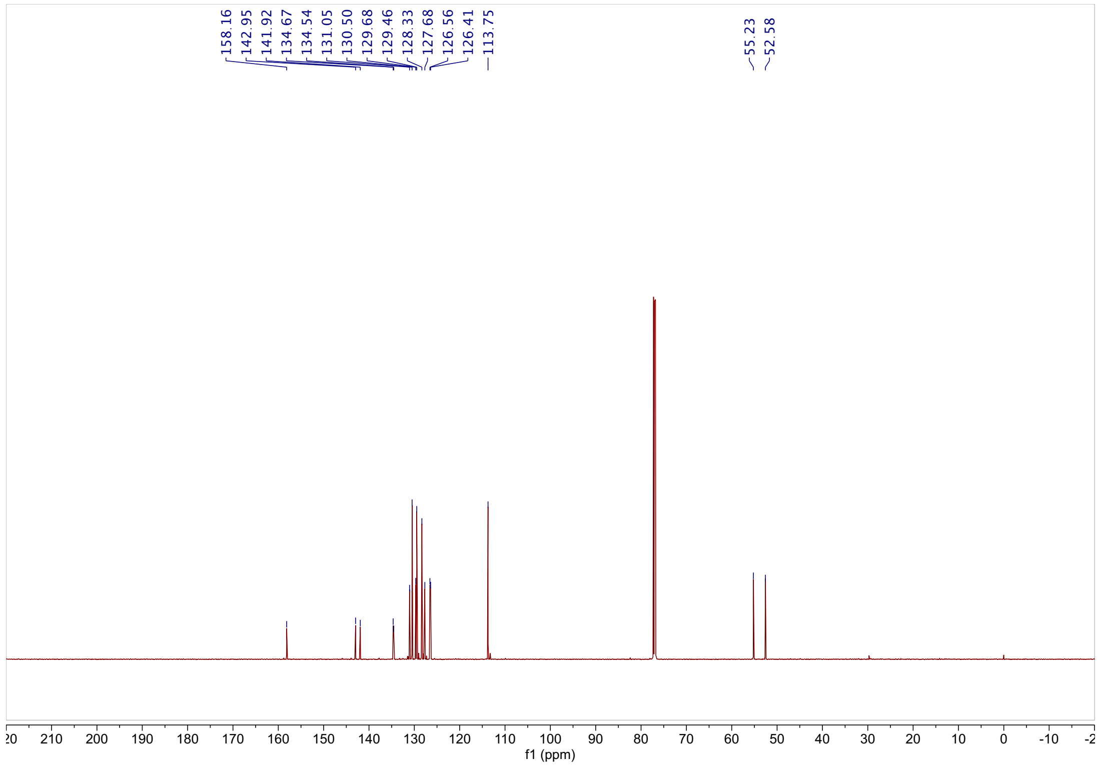

Compound 19

^{1}H NMR (400 MHz, $CDCl_3$)

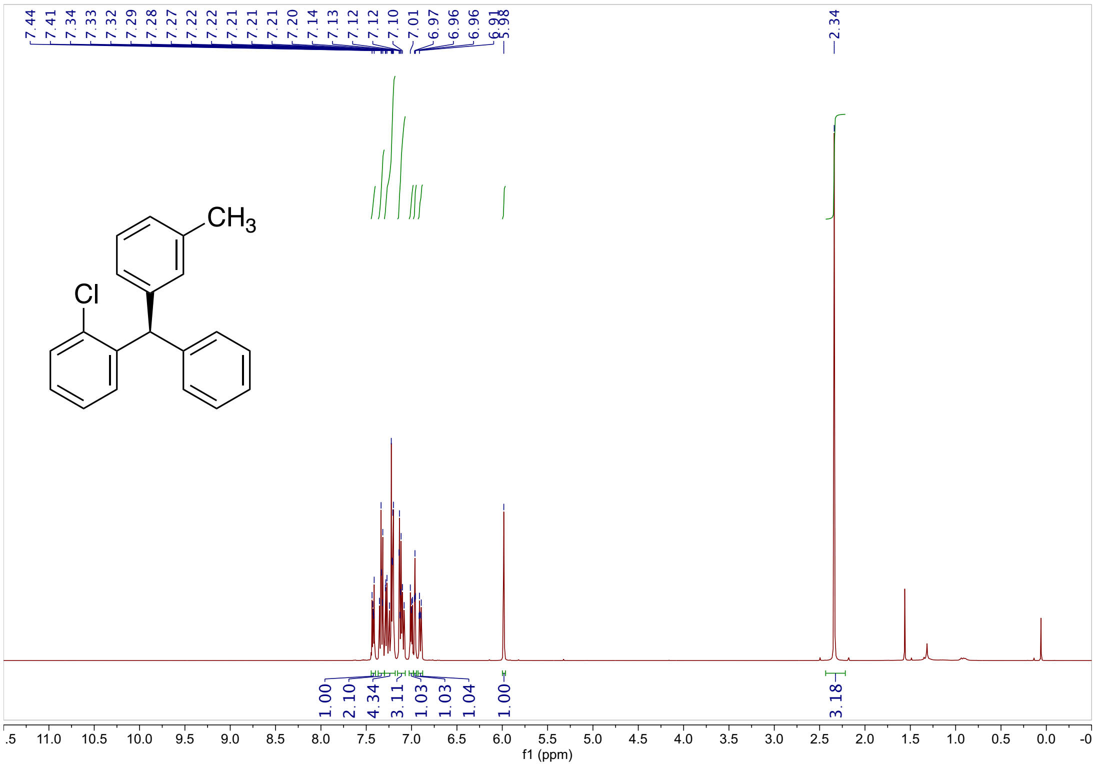

^{13}C NMR (600 MHz, $CDCl_3$)

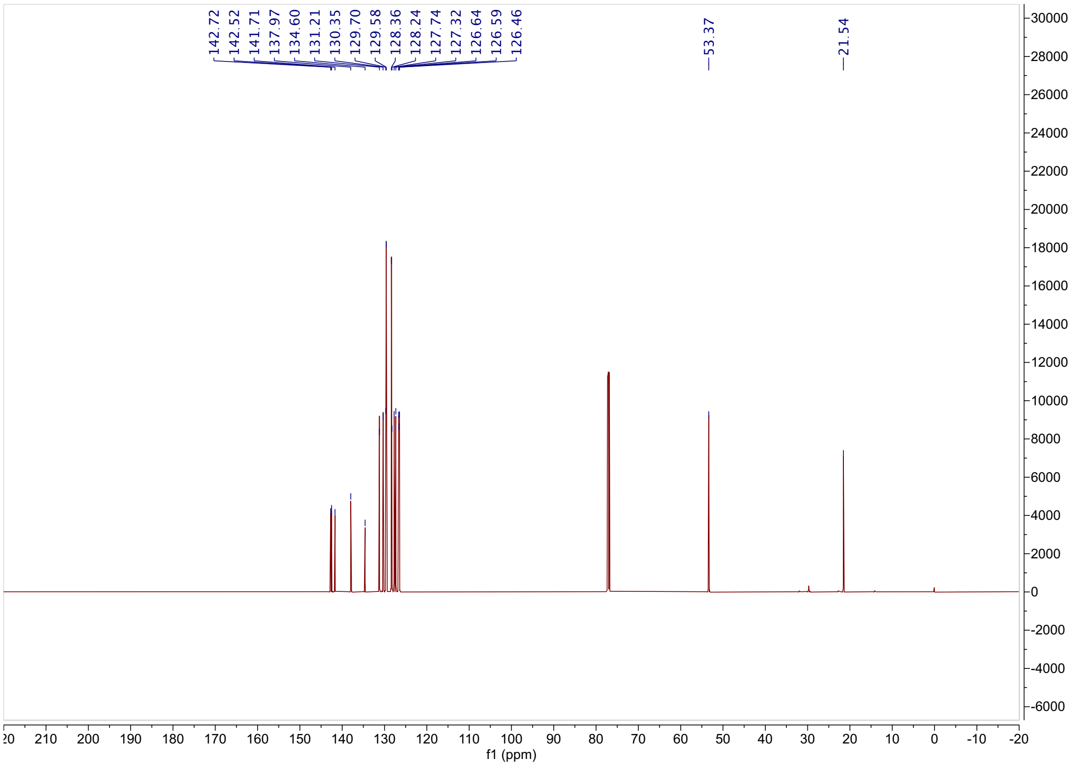

Compound 20

[1]**H NMR** (600 MHz, $CDCl_3$)

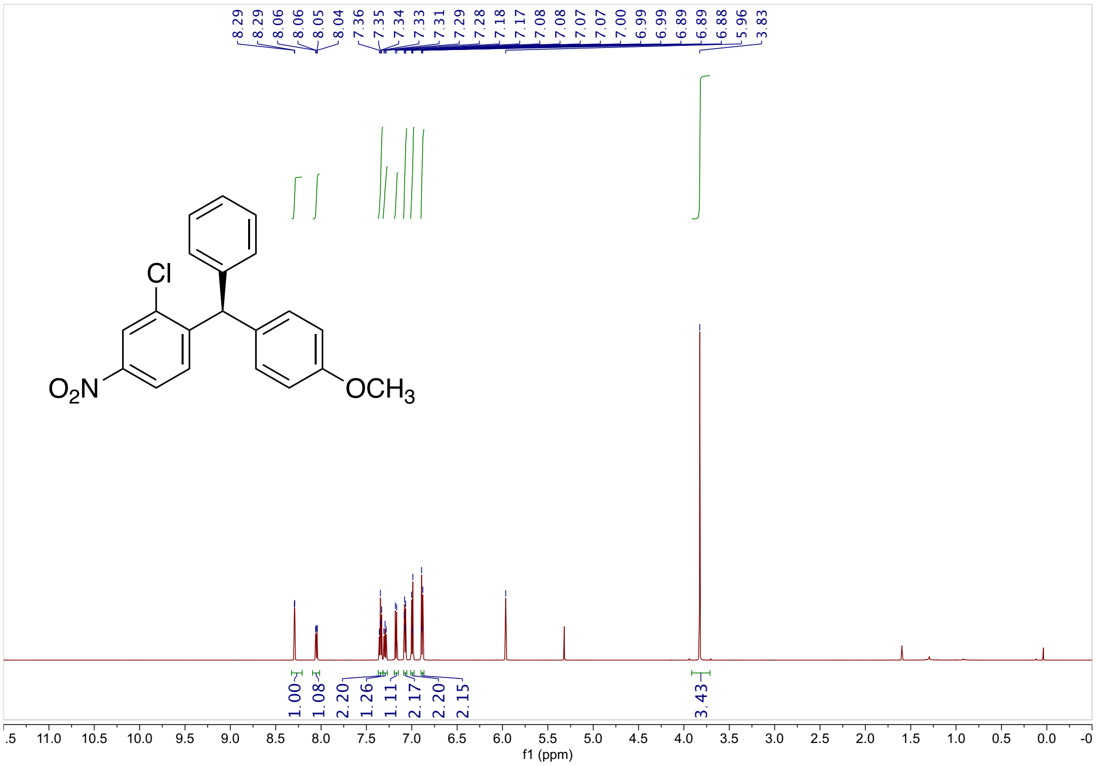

[13]**C NMR** (600 MHz, $CDCl_3$)

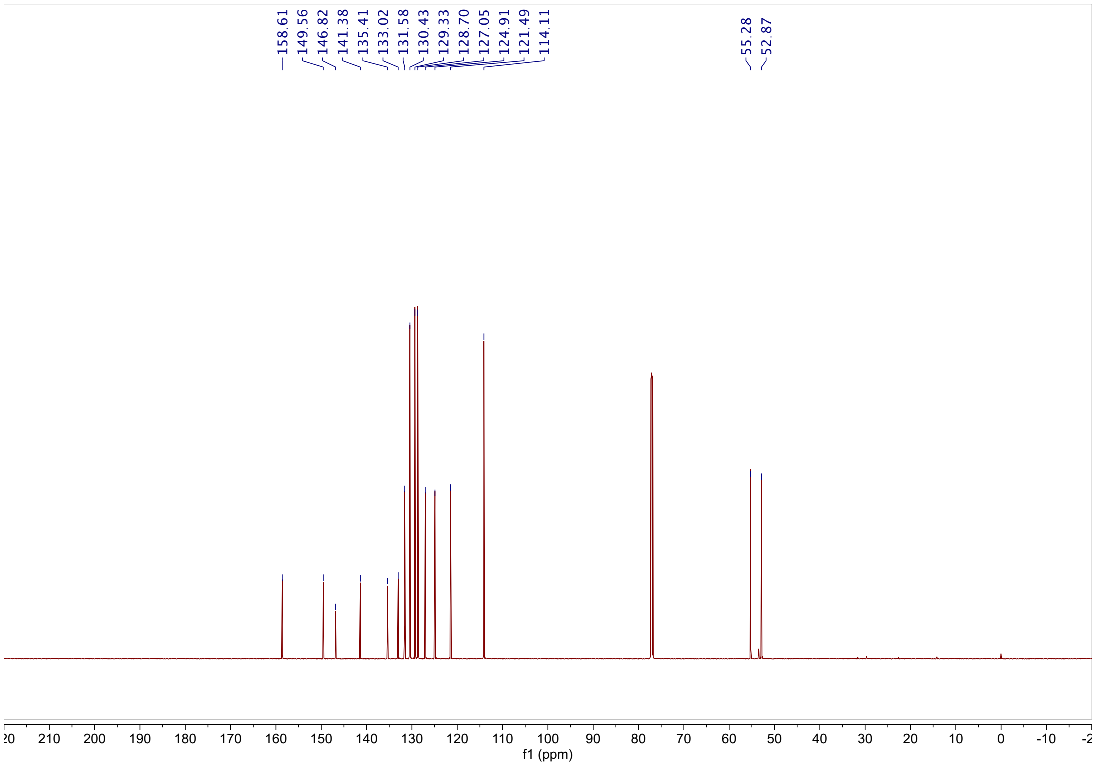

Compound 21

^{1}H NMR (600 MHz, $CDCl_3$)

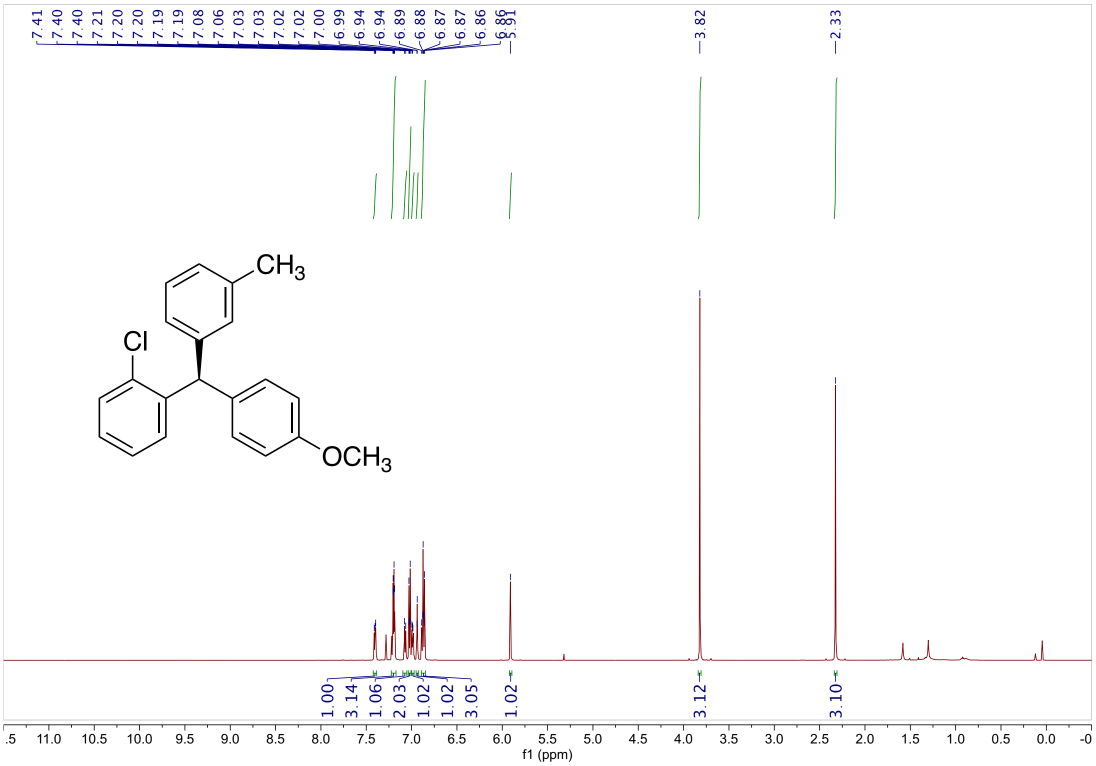

^{13}C NMR (600 MHz, $CDCl_3$)

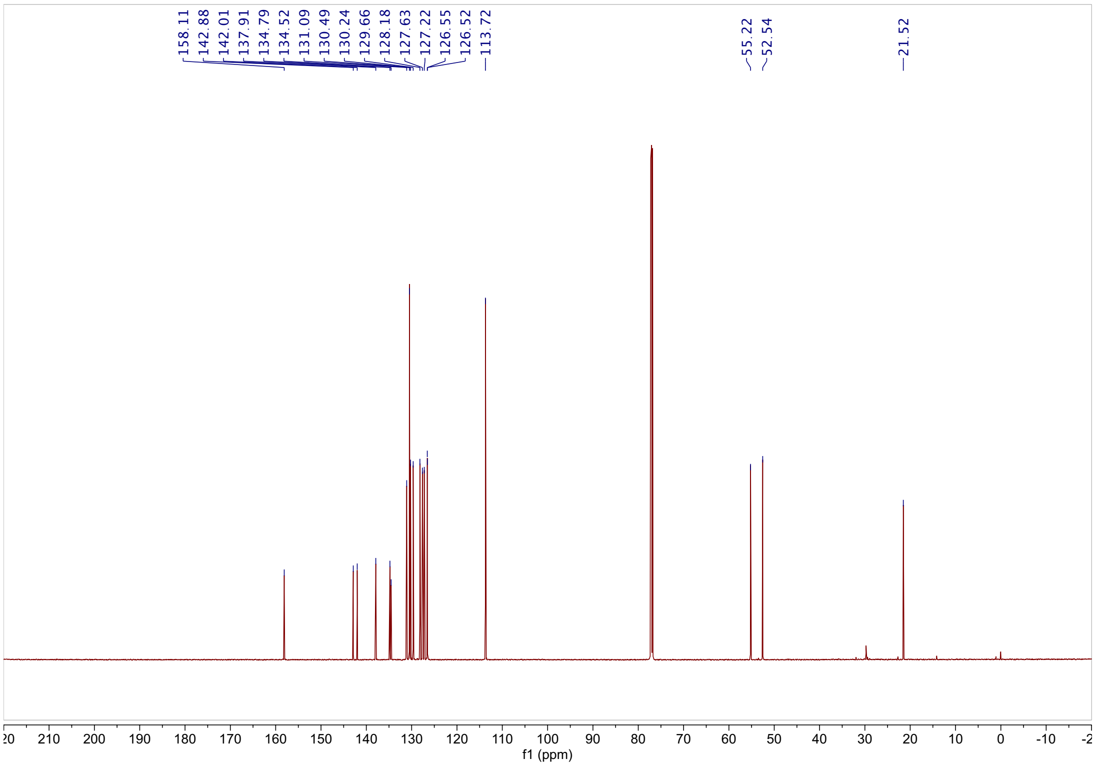

Compound 22

^{1}H NMR (600 MHz, $CDCl_3$)

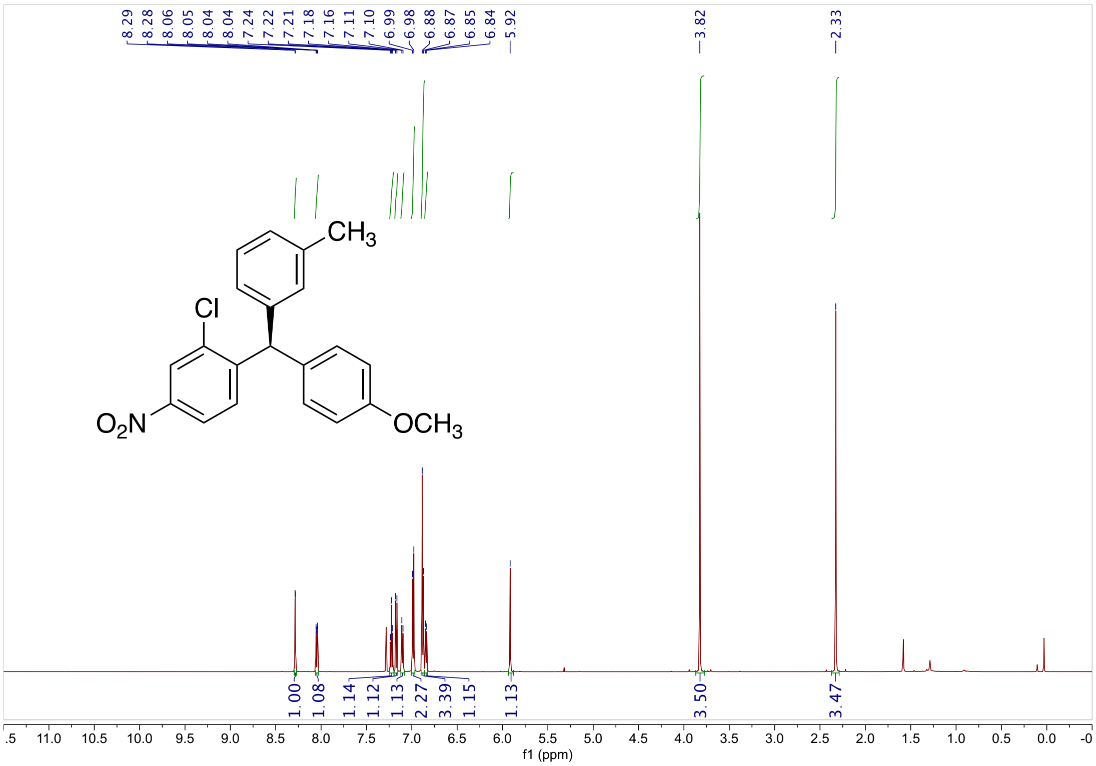

^{13}C NMR (600 MHz, $CDCl_3$)

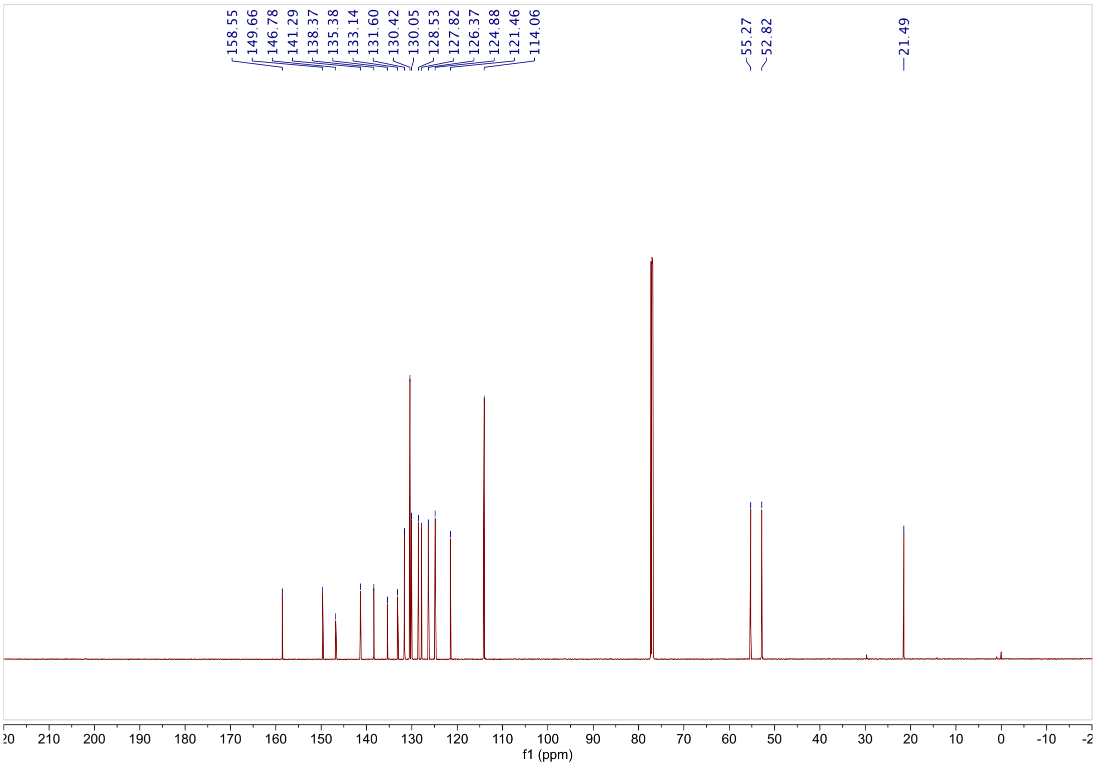

Compound 23

^{1}H NMR (600 MHz, CDCl$_3$)

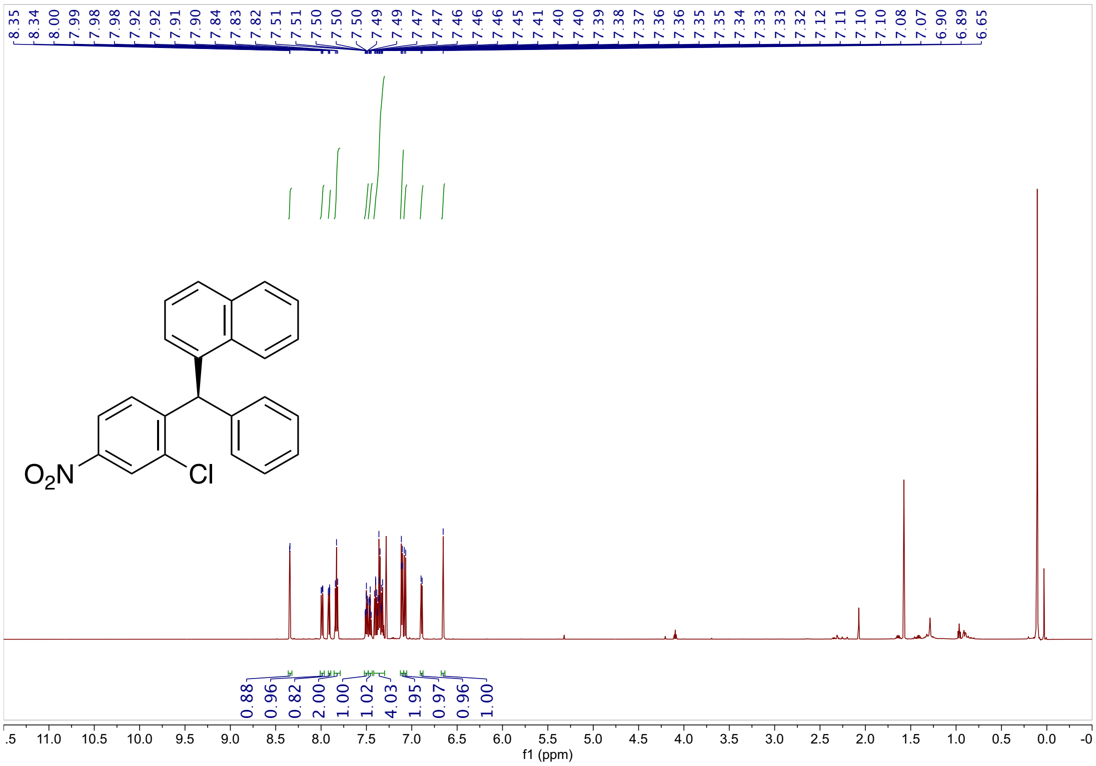

^{13}C NMR (600 MHz, CDCl$_3$)

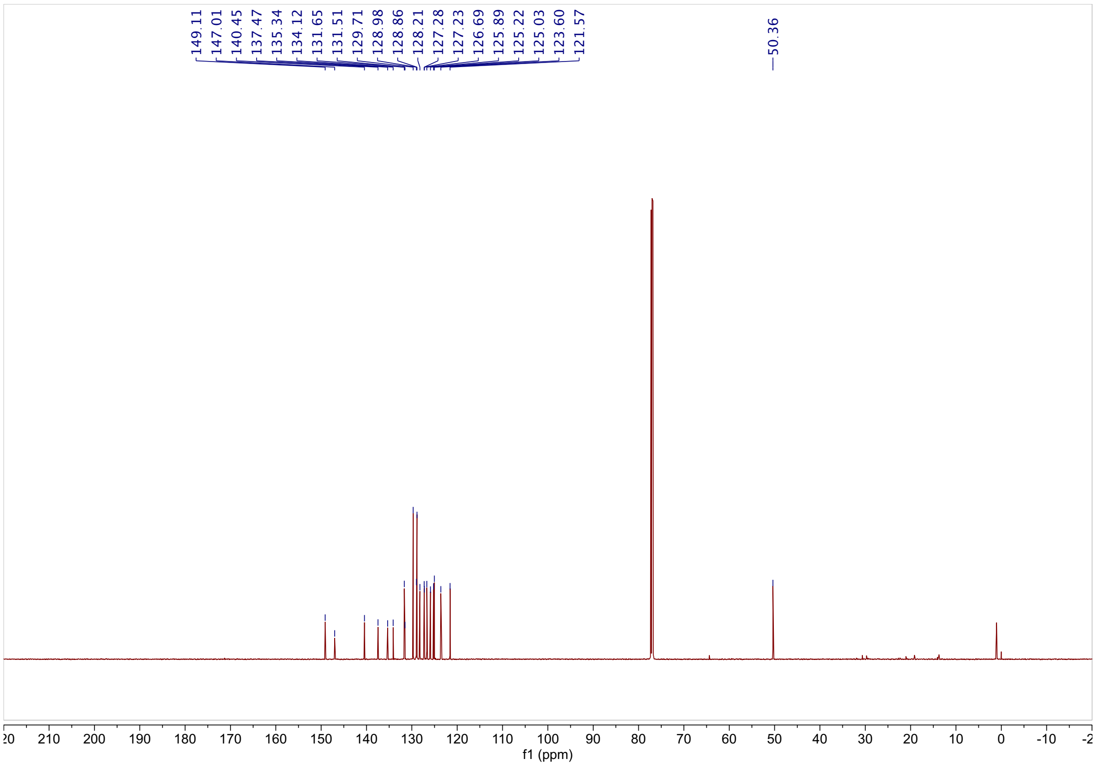

Compound 24

^{1}H NMR (400 MHz, $CDCl_3$)

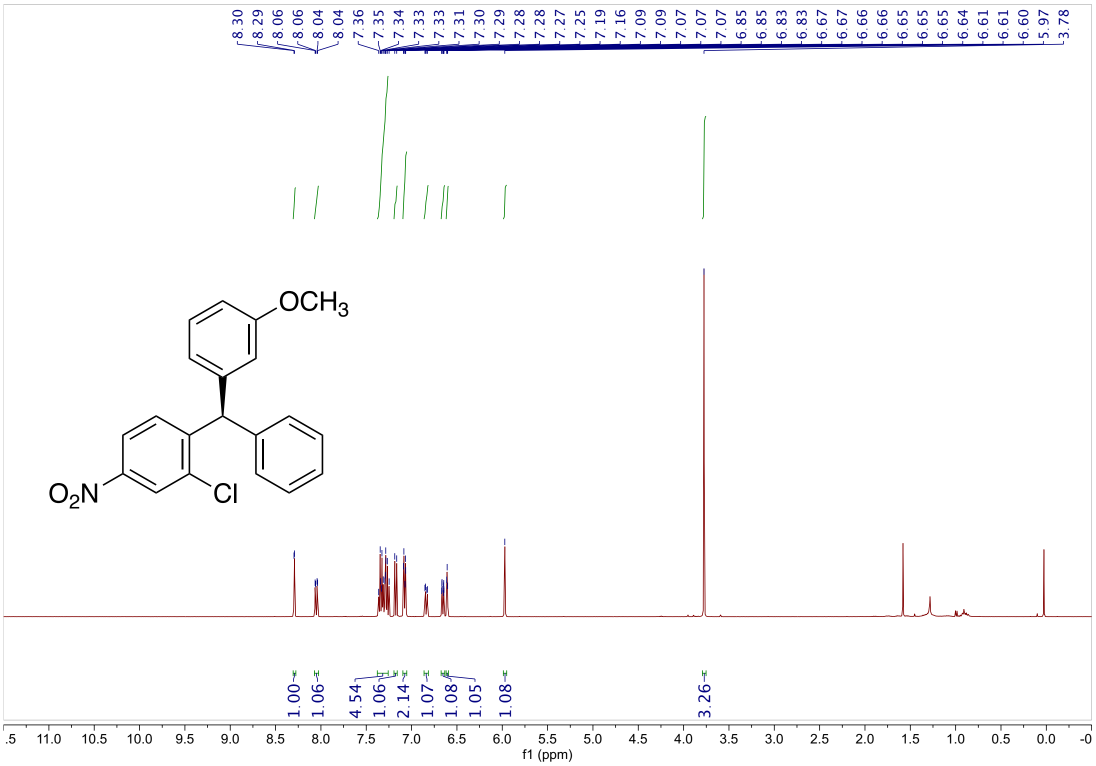

^{13}C NMR (400 MHz, $CDCl_3$)

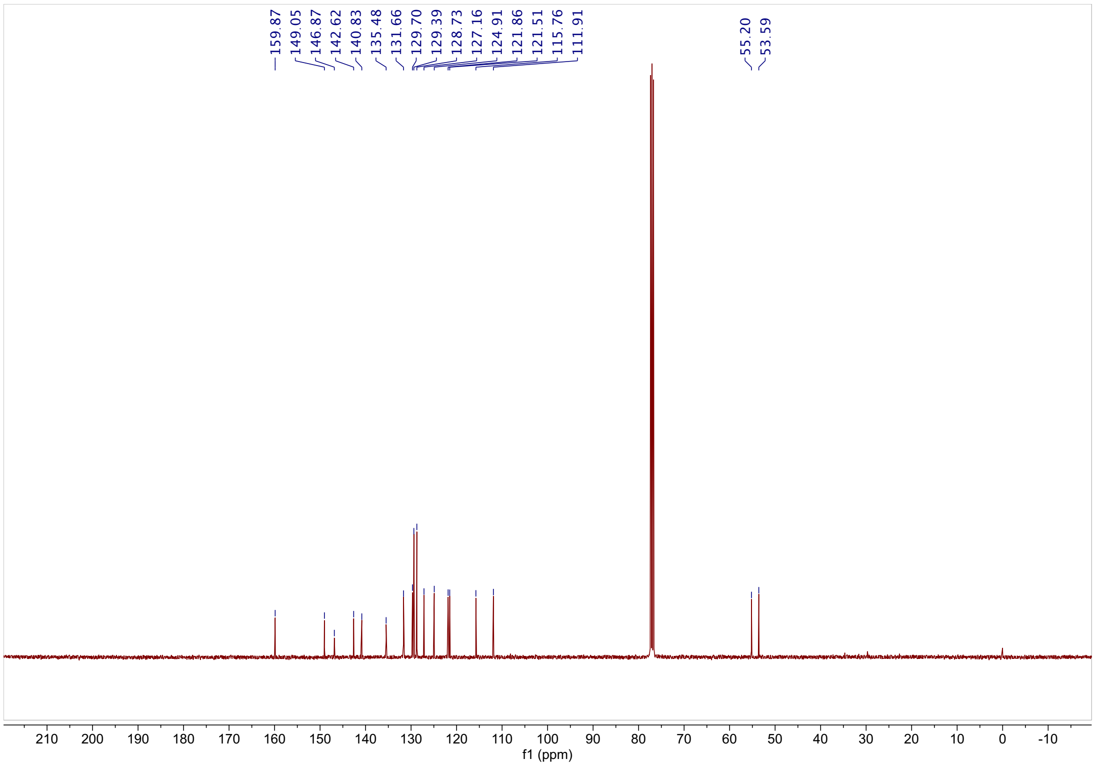

Compound 25

^{1}H NMR (400 MHz, $CDCl_3$)

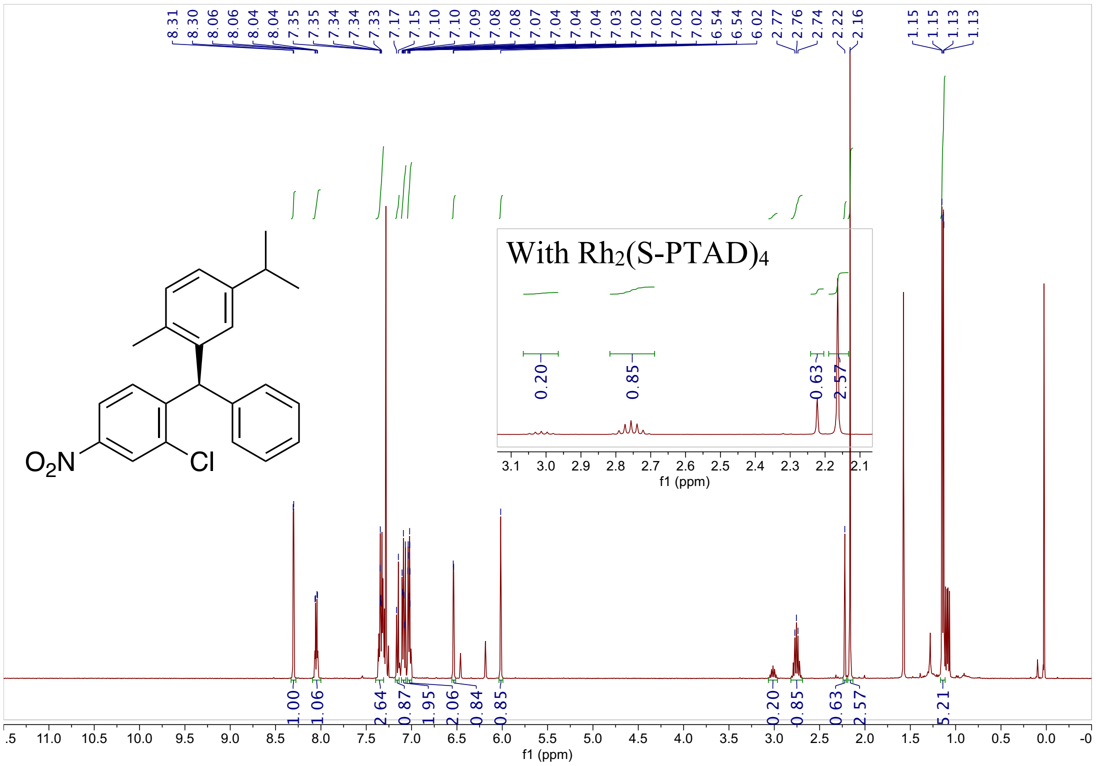

^{13}C NMR (400 MHz, $CDCl_3$)

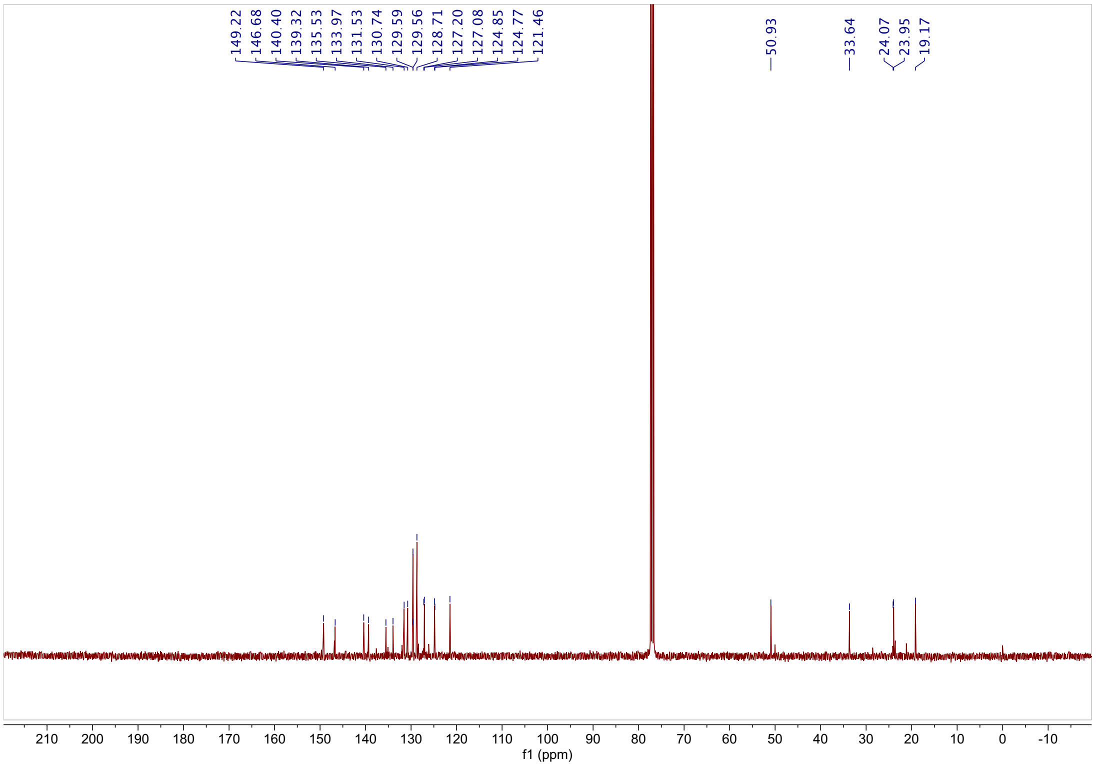

Compound 25 NOE
^{1}H-^{1}H COSY NMR (400 MHz, $CDCl_3$)

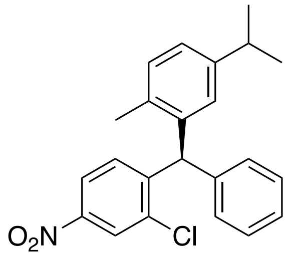

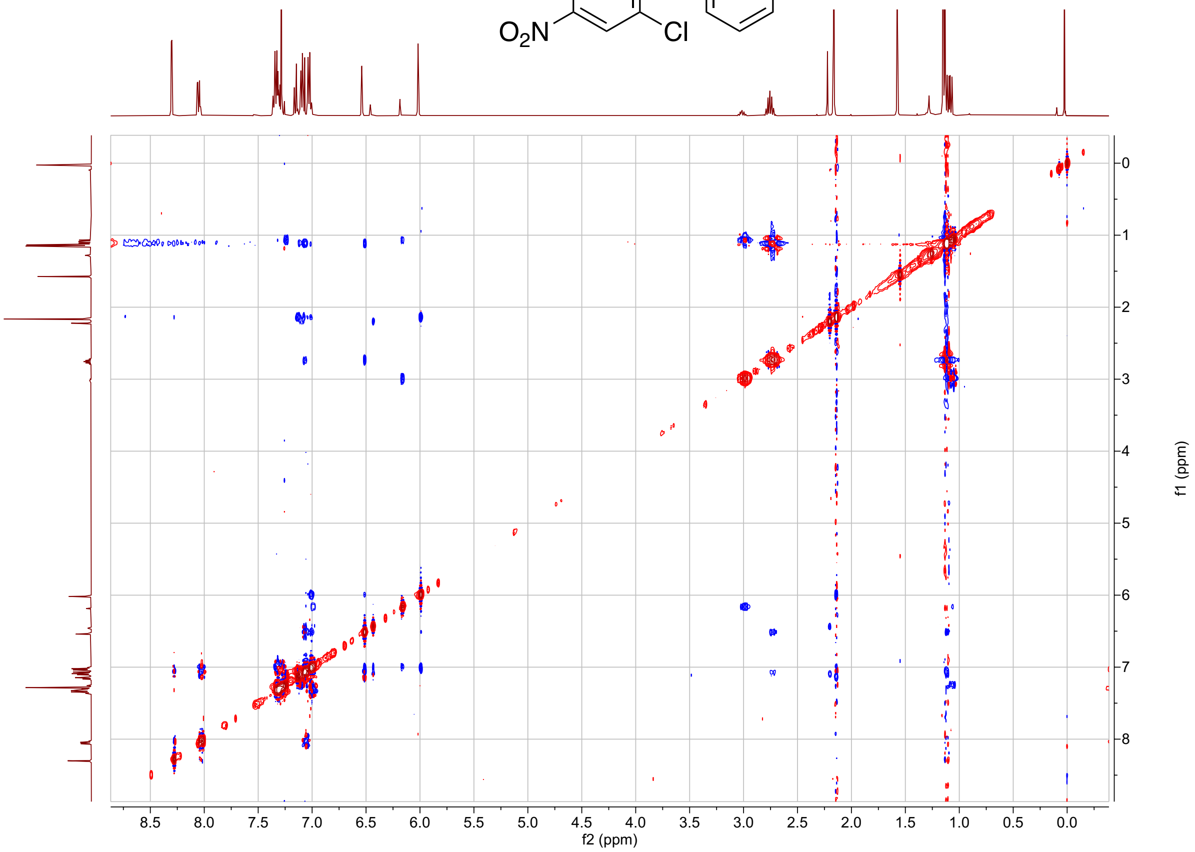

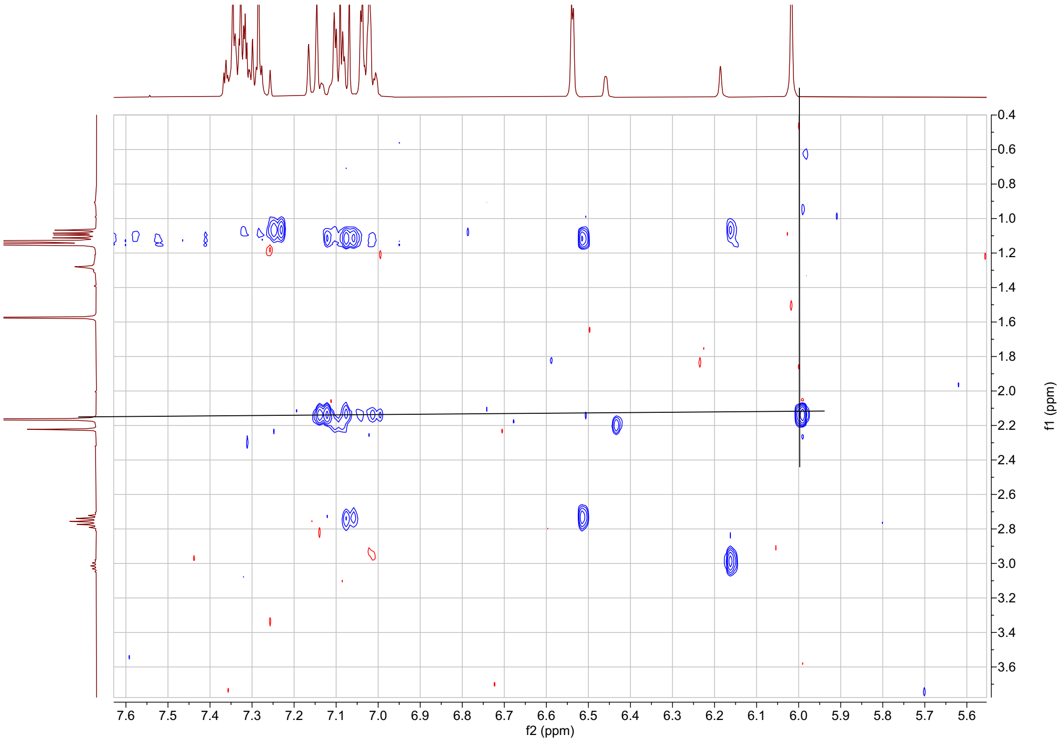

Compound 25
Crude ^{1}**H NMR** (400 MHz, $CDCl_3$)
Selectivity with Rh_2(S-TPPTTL)$_4$

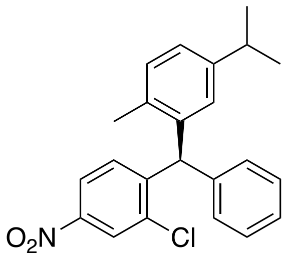

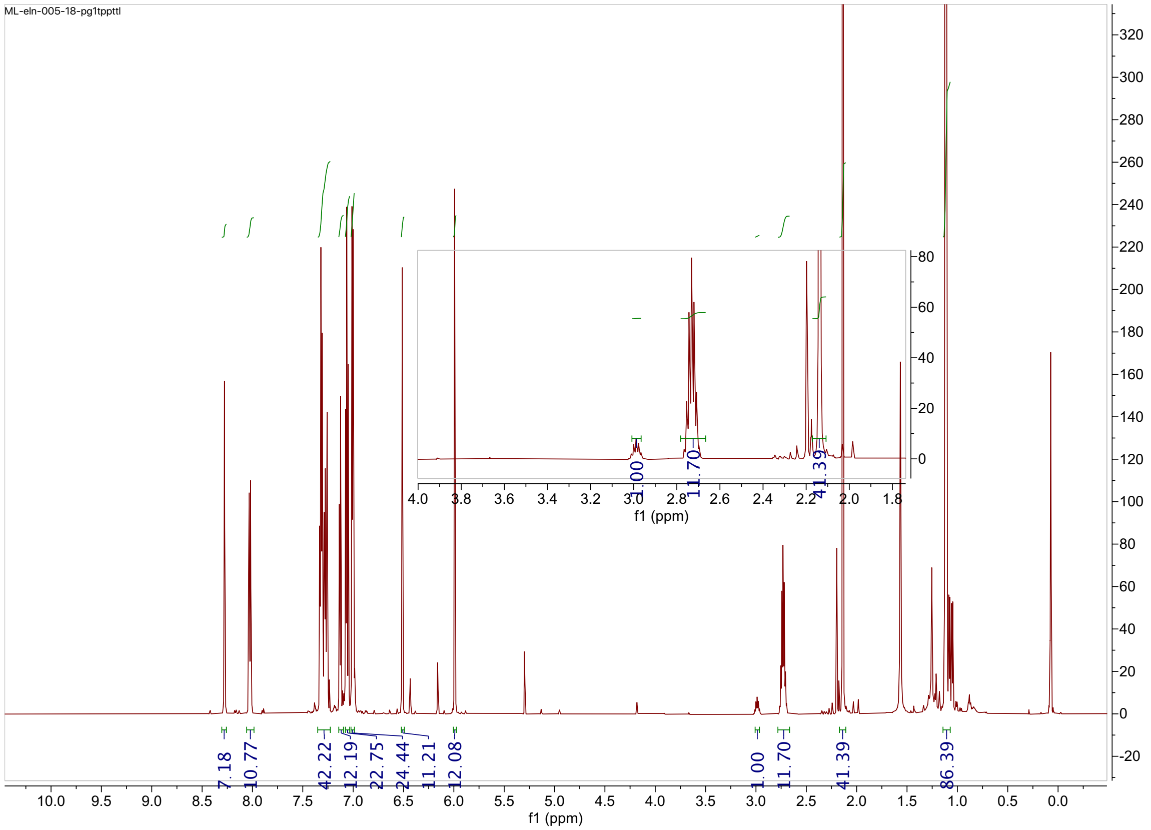

6. X-Ray Crystallographic Data for Compound 11a.

Crystal Data and Experimental

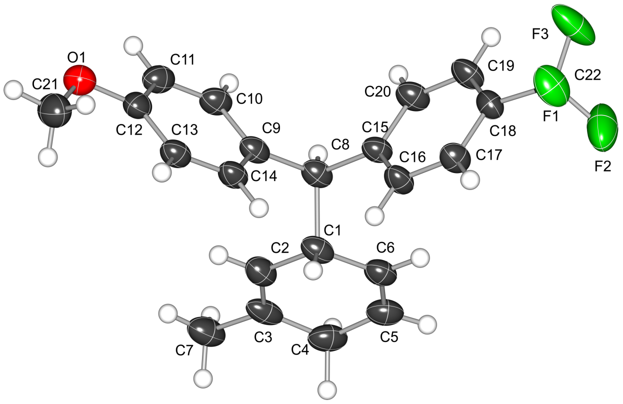

Experimental. Single colorless plate-shaped crystals of **Compound 11a.** were chosen from the sa mple as supplied. A suitable crystal with dimensions 0.36 × 0.27 × 0.14 mm³ was selected and mounted on a loop with paratone on a XtaLAB Synergy diffractometer. The crystal was kept at a steady T = 100.00(10) K during data collection. The structure was solved with the ShelXT 2018/2 (Sheldrick, 2018) solution program using dual methods. The model was refined with **Jana2020** (Palatinus, 2020) using least squares minimisation on $\boldsymbol{F^2}$.

Crystal Data. $C_{22}H_{21}F_3O$, M_r = 358.4, monoclinic, $P2_1$ (No. 4), a = 7.331(2) Å, b = 6.002(2) Å, c = 21.088(4) Å, β = 105.13(3)°, $\alpha = \gamma$ = 90°, V = 895.7(4) Å³, T = 100.00(10) K, Z = 2, Z' = 1, μ(Cu K_α) = 0.846, 11165 reflections measured, 3291 unique (R_{int} = 0.0536) which were used in all calculations. The final wR_2 was 0.1293 (all data) and R_1 was 0.0513 (I≥3 σ(I)).

**Compound**	**I**
Formula	$C_{22}H_{21}F_3O$
$D_{calc.}$/ g cm^{-3}	1.3289
μ/mm^{-1}	0.846
Formula Weight	358.4
Colour	colourless
Shape	plate-shaped
Size/mm³	0.36×0.28×0.14
T/K	100.00(10)
Crystal System	monoclinic
Flack Parameter	?
Space Group	$P2_1$
a/Å	7.331(2)
b/Å	6.002(2)
c/Å	21.088(4)
α/°	90
β/°	105.13(3)
γ/°	90
V/Å³	895.7(4)
Z	2
Z'	1
Wavelength/Å	1.54184
Radiation type	Cu K_α
Θ_{min}/°	4.34
Θ_{max}/°	73.14
Measured Refl's.	11165
Indep't Refl's	3291
Refl's I≥3 σ(I)	3073
R_{int}	0.0536
Parameters	236
Restraints	0
Largest Peak	0.29
Deepest Hole	-0.17
GooF	2.3460
wR_2 (all data)	0.1293
wR_2	0.1281
R_1 (all data)	0.0538
R_1	0.0513

Structure Quality Indicators

**Reflections:**	d min (Cu\a) 2Θ=146.3° 0.81	I/σ(I) CIF 22.3	Rint CIF 5.36%	Full 143.0° 96% to 146.3° 98.0	
**Refinement:**	Shift CIF 0.013	Max Peak CIF 0.3	Min Peak CIF -0.2	GooF CIF 2.346	Hooft ?()

A colourless plate-shaped-shaped crystal with dimensions 0.36 × 0.28 × 0.14 mm³ was mounted on a loop with paratone. Data were collected using a XtaLAB Synergy, Dualflex, HyPix diffractometer operating at T = 100.00(10) K.

Data were measured using ω scans with Cu K_α radiation. The diffraction pattern was indexed and the total number of runs and images was based on the strategy calculation from the program CrysAlisPro 1.171.42.74a (Rigaku OD, 2022). The maximum resolution that was achieved was Θ = 73.14° (0.81 Å).

The unit cell was refined using CrysAlisPro 1.171.42.74a (Rigaku OD, 2022) on 9940 reflections, 89% of the observed reflections.

Data reduction, scaling and absorption corrections were performed using CrysAlisPro 1.171.42.74a (Rigaku OD, 2022). The final completeness is 99.80 % out to 73.211° in Θ. A numerical absorption correction based on gaussian integration over a multifaceted crystal model was performed using CrysAlisPro 1.171.42.74a (Rigaku Oxford Diffraction, 2022). An empirical absorption correction using spherical harmonics, implemented in SCALE3 ABSPACK scaling algorithm was also applied. The absorption coefficient μ of this material is 0.846 mm^{-1} at this wavelength (λ = 1.54184Å) and the minimum and maximum transmissions are 0.523 and 1.000.

The crystal was a reticular pseudomerohedric twin. The unit cell is monoclinic with dimensions a=7.3341 b=6.0032 c=20.4351 89.85 95.13 90.02. The twin is a rotation of 180 degrees about the a-axis. The twin gives two superimposing lattices (the original one and the transformed one). The combined lattice can be indexed with a unit cell with a=7.33 b=6.00 c=40.88 89.85 95.13 90.02 (i.e. with a doubled c-axis). We get much better data if I integrate the data using the combined lattice (the data integration software did not handle the twinning very well). The program Jana2020 was used to separate the two twin domains and refine the structure

The structure was solved and the space group $P2_1$ (# 4) determined by the ShelXT 2018/2 (Sheldrick, 2018) structure solution program using dual methods and refined by full matrix least squares minimisation on $\boldsymbol{F}^2$ using version Jana 2020 (Palatinus, 2020). All non-hydrogen atoms were refined anisotropically. Hydrogen atom positions were calculated geometrically and refined using the riding model. *_geom_special_details*: Bond distances, angles etc. have been calculated using the rounded fractional coordinates. All su's are estimated from the variances of the (full) variance-covariance matrix. The cell esds are taken into account in the estimation of distances, angles and torsion angles

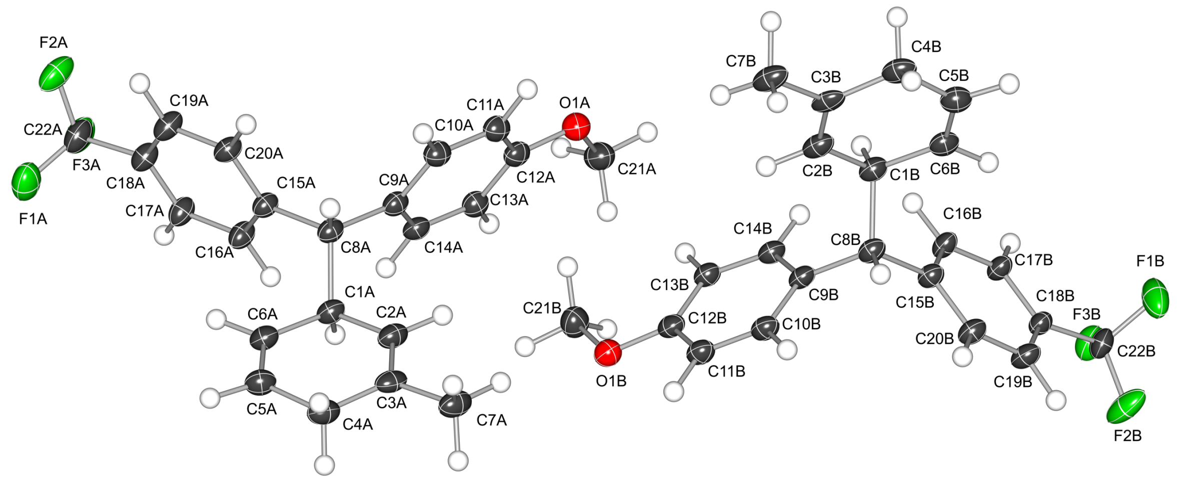

Figure 1: The repeating unit for the two overlapping twin domains treated as a single crystal

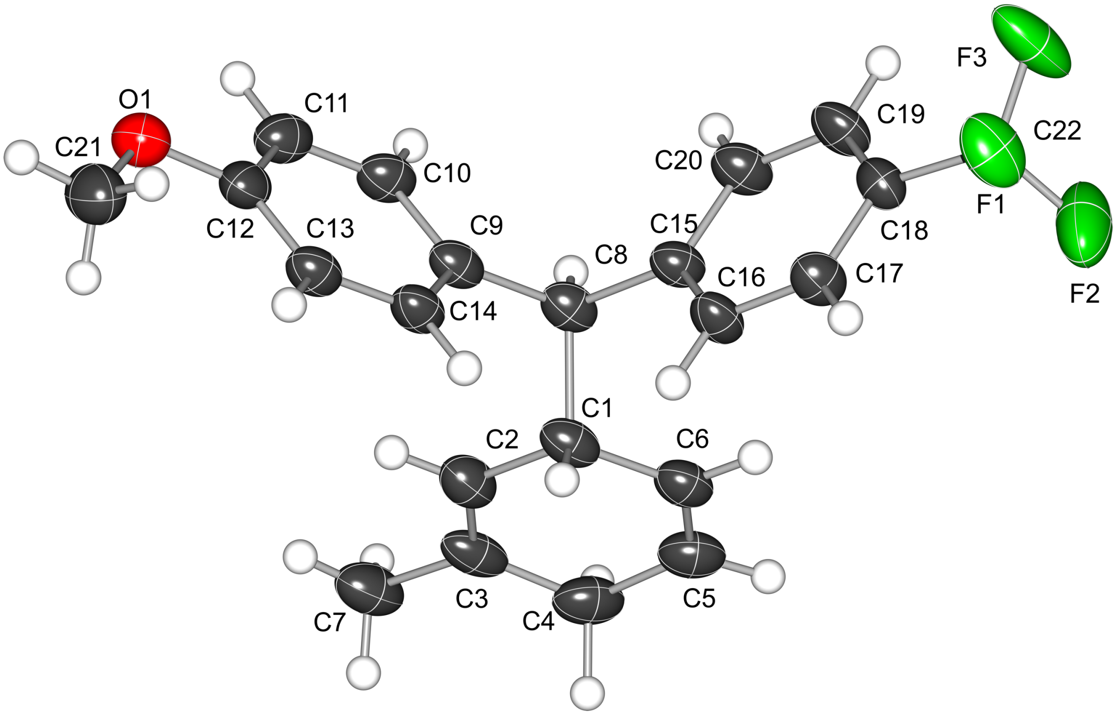

Figure 2: The asymmetric unit of the transformed cell

Table 1: Fractional Atomic Coordinates (×10⁴) and Equivalent Isotropic Displacement Parameters (Å²×10³) for **I**. U_{eq} is defined as 1/3 of the trace of the orthogonalised U_{ij}.

Atom	x	y	z	U_{eq}
F1	7159(3)	10894(3)	4690.1(9)	57.3(7)
O1	2976(3)	7498(4)	24.6(10)	48.8(8)
F2	6500(4)	8314(5)	5265.4(9)	79.1(9)
F3	9000(3)	8115(4)	4913.9(11)	76.4(8)
C18	6084(4)	7548(5)	4139.1(13)	35.3(8)
C16	3222(4)	7250(5)	3278.5(13)	38.8(9)
C13	1878(4)	7855(5)	1022.6(13)	38.7(9)
C14	1826(4)	6954(5)	1618.0(13)	38.3(9)
C9	2681(3)	4923(5)	1842.2(13)	35.5(8)

Atom	x	y	z	U_{eq}
C22	7202(4)	8690(5)	4744.7(14)	42.0(10)
C10	3625(4)	3839(5)	1434.3(14)	39.6(9)
C12	2823(4)	6734(5)	622.7(14)	39.6(10)
C15	3815(4)	5229(4)	3069.0(13)	34.6(8)
C17	4331(4)	8384(5)	3810.1(14)	38.7(9)
C6	484(4)	2721(5)	3211.0(15)	40.1(9)
C3	-1692(4)	424(5)	2092.0(16)	44.3(10)
C2	-599(4)	2139(6)	2011.0(15)	42.5(10)
C11	3705(4)	4728(5)	837.4(14)	41.6(10)
C19	6701(4)	5562(5)	3926.8(14)	43.0(10)
C5	-581(4)	997(6)	3304.6(18)	45.6(11)
C1	535(4)	3579(5)	2551.1(14)	38.0(9)
C20	5586(4)	4445(5)	3405.6(14)	41.9(10)
C8	2599(3)	3910(5)	2494.9(13)	36.4(9)
C7	-2802(4)	-954(6)	1529.1(17)	53.4(12)
C4	-1839(4)	-280(6)	2756.4(16)	50.6(11)
C21	1953(5)	9457(7)	-223.3(16)	55.1(12)

Table 2: Anisotropic Displacement Parameters (×10[4]) for **I.** The anisotropic displacement factor exponent takes the form: $-2\pi^2[h^2a^{*2} \times U_{11} + ... + 2hka^* \times b^* \times U_{12}]$

Atom	U_{11}	U_{22}	U_{33}	U_{23}	U_{13}	U_{12}
F1	62.0(11)	36.6(11)	62.4(11)	-3.6(8)	-3.1(9)	-12.2(8)
O1	41.1(10)	57.4(15)	47.9(11)	3.1(10)	11.4(9)	-0.1(10)
F2	92.5(16)	96.3(19)	43.5(10)	-41.1(14)	8.5(10)	-4.9(11)
F3	44.9(10)	65.9(15)	95.2(16)	11.4(10)	-23.2(10)	-28.8(13)
C18	32.6(12)	34.8(16)	34.4(13)	-0.2(11)	1.5(10)	2.2(10)
C16	31.0(13)	39.3(17)	41.1(14)	9.5(11)	0.3(11)	-5.0(12)
C13	31.8(12)	37.2(16)	44.4(15)	6.0(12)	5.2(11)	-5.7(12)
C14	29.9(12)	40.5(16)	41.5(15)	7.9(11)	4.1(11)	-7.1(12)
C9	27.9(12)	31.4(14)	43.3(14)	3.2(11)	2.5(11)	-9.6(11)
C22	39.8(15)	43.5(19)	37.2(15)	-1.9(12)	0.1(12)	3.4(12)
C10	33.0(13)	35.6(15)	49.7(16)	7.5(11)	9.9(12)	-8.4(13)
C12	30.1(13)	48.9(18)	38.5(15)	-2.4(12)	6.8(11)	-7.2(13)
C15	27.8(12)	31.5(15)	43.2(14)	3.0(10)	6.5(10)	-5.1(11)
C17	37.1(13)	31.8(15)	44.8(15)	6.5(11)	6.3(12)	-7.0(11)
C6	29.1(12)	39.1(17)	49.7(16)	5.1(11)	6.2(12)	-4.3(12)
C3	25.9(12)	36.4(17)	66.2(18)	6.2(10)	4.1(12)	-18.5(14)
C2	35.7(13)	41.1(17)	47.1(16)	3.9(11)	4.3(12)	-10.5(13)
C11	36.1(14)	41.4(17)	48.7(16)	-0.7(12)	13.7(12)	-11.5(13)
C19	32.1(13)	39.5(18)	50.3(16)	9.4(12)	-1.7(12)	-0.3(13)
C5	33.4(14)	41.1(17)	64.3(19)	5.5(12)	16.3(13)	-3.9(14)
C1	29.2(12)	28.9(15)	51.8(16)	3.6(10)	3.5(11)	-6.9(11)
C20	34.4(13)	34.4(17)	53.7(17)	7.2(11)	5.9(12)	-4.9(13)
C8	31.8(13)	28.2(15)	45.9(15)	5.9(10)	4.5(11)	-6.0(11)
C7	35.2(15)	51(2)	70(2)	0.4(13)	7.1(14)	-19.7(16)
C4	36.9(14)	45.3(18)	73(2)	3.6(13)	21.3(14)	-12.1(16)
C21	49.6(17)	64(2)	51.2(18)	1.0(16)	11.8(15)	9.4(16)

Table 3: Bond Lengths in Å for **I.**

Atom	Atom	Length/Å	Atom	Atom	Length/Å
F1	C22	1.328(4)	F2	C22	1.348(4)

Atom	Atom	Length/Å
F3	C22	1.319(4)
O1	C12	1.374(4)
O1	C21	1.419(5)
C1	C2	1.497(4)
C1	C6	1.493(4)
C1	C8	1.561(4)
C2	C3	1.343(5)
C3	C4	1.494(5)
C3	C7	1.500(5)
C4	C5	1.489(5)
C5	C6	1.342(5)
C8	C9	1.520(4)
C8	C15	1.524(4)
C9	C10	1.398(4)
C9	C14	1.396(4)
C10	C11	1.382(4)
C11	C12	1.386(4)
C12	C13	1.396(4)
C13	C14	1.377(4)
C15	C16	1.399(4)
C15	C20	1.389(4)
C16	C17	1.380(4)
C17	C18	1.385(4)
C18	C19	1.390(4)

Atom	Atom	Length/Å
C18	C22	1.492(4)
C19	C20	1.363(4)
C1	H1c1	0.9600
C2	H1c2	0.9600
C4	H1c4	1.0600
C4	H2c4	1.0600
C5	H1c5	0.9600
C6	H1c6	0.9600
C7	H1c7	0.9600
C7	H2c7	0.9600
C7	H3c7	0.9600
C8	H1c8	0.9600
C10	H1c10	0.9600
C11	H1c11	0.9600
C13	H1c13	0.9600
C14	H1c14	0.9600
C16	H1c16	0.9600
C17	H1c17	0.9600
C19	H1c19	0.9600
C20	H1c20	0.9600
C21	H1c21	0.9600
C21	H2c21	0.9600
C21	H3c21	0.9600

Table 4: Bond Angles in ° for **I.**

Atom	Atom	Atom	Angle/°
C12	O1	C21	116.9(2)
C2	C1	C6	111.5(3)
C2	C1	C8	111.9(2)
C6	C1	C8	112.0(2)
C1	C2	C3	125.3(3)
C2	C3	C4	121.9(3)
C2	C3	C7	122.7(3)
C4	C3	C7	115.4(3)
C3	C4	C5	113.6(3)
C4	C5	C6	123.3(3)
C1	C6	C5	124.1(3)
C1	C8	C9	112.8(2)
C1	C8	C15	112.5(2)
C9	C8	C15	111.0(2)
C8	C9	C10	120.9(3)
C8	C9	C14	122.4(2)
C10	C9	C14	116.7(3)
C9	C10	C11	121.8(3)
C10	C11	C12	120.4(3)
O1	C12	C11	116.6(3)
O1	C12	C13	124.5(3)
C11	C12	C13	119.0(3)
C12	C13	C14	119.9(3)
C9	C14	C13	122.4(3)
C8	C15	C16	122.4(3)
C8	C15	C20	120.3(2)
C16	C15	C20	117.3(3)
C15	C16	C17	121.2(3)
C16	C17	C18	120.1(3)

Atom	Atom	Atom	Angle/°
C17	C18	C19	119.1(3)
C17	C18	C22	119.5(3)
C19	C18	C22	121.2(3)
C18	C19	C20	120.3(3)
C15	C20	C19	122.0(3)
F1	C22	F2	103.4(3)
F1	C22	F3	106.5(3)
F1	C22	C18	112.9(2)
F2	C22	F3	107.6(3)
F2	C22	C18	112.3(3)
F3	C22	C18	113.5(3)
C2	C1	H1c1	107.00
C6	C1	H1c1	107.00
C8	C1	H1c1	107.00
C1	C2	H1c2	117.00
C3	C2	H1c2	117.00
C3	C4	H1c4	109.00
C3	C4	H2c4	109.00
C5	C4	H1c4	109.00
C5	C4	H2c4	109.00
H1c4	C4	H2c4	105.00
C4	C5	H1c5	118.00
C6	C5	H1c5	118.00
C1	C6	H1c6	118.00
C5	C6	H1c6	118.00
C3	C7	H1c7	109.00
C3	C7	H2c7	109.00
C3	C7	H3c7	109.00
H1c7	C7	H2c7	109.00

Atom	Atom	Atom	Angle/°
H1c7	C7	H3c7	109.00
H2c7	C7	H3c7	109.00
C1	C8	H1c8	105.00
C9	C8	H1c8	107.00
C15	C8	H1c8	107.00
C9	C10	H1c10	119.00
C11	C10	H1c10	119.00
C10	C11	H1c11	120.00
C12	C11	H1c11	120.00
C12	C13	H1c13	120.00
C14	C13	H1c13	120.00
C9	C14	H1c14	119.00
C13	C14	H1c14	119.00
C15	C16	H1c16	119.00

Atom	Atom	Atom	Angle/°
C17	C16	H1c16	119.00
C16	C17	H1c17	120.00
C18	C17	H1c17	120.00
C18	C19	H1c19	120.00
C20	C19	H1c19	120.00
C15	C20	H1c20	119.00
C19	C20	H1c20	119.00
O1	C21	H1c21	109.00
O1	C21	H2c21	109.00
O1	C21	H3c21	109.00
H1c21	C21	H2c21	109.00
H1c21	C21	H3c21	109.00
H2c21	C21	H3c21	109.00

Table 5: Torsion Angles in ° for **I**.

Atom	Atom	Atom	Atom	Angle/°
C21	O1	C12	C11	175.1(3)
C21	O1	C12	C13	-6.0(4)
C6	C1	C2	C3	6.5(4)
C8	C1	C2	C3	132.8(3)
C2	C1	C6	C5	-5.6(4)
C8	C1	C6	C5	-131.8(3)
C2	C1	C8	C9	57.7(3)
C2	C1	C8	C15	-175.8(2)
C6	C1	C8	C9	-176.3(2)
C6	C1	C8	C15	-49.8(3)
C1	C2	C3	C4	-2.0(5)
C1	C2	C3	C7	179.8(3)
C2	C3	C4	C5	-3.7(4)
C7	C3	C4	C5	174.7(3)
C3	C4	C5	C6	4.5(5)
C4	C5	C6	C1	0.4(5)
C1	C8	C9	C10	-123.3(3)
C1	C8	C9	C14	56.5(4)
C15	C8	C9	C10	109.4(3)
C15	C8	C9	C14	-70.9(3)
C1	C8	C15	C16	-49.5(4)
C1	C8	C15	C20	130.7(3)
C9	C8	C15	C16	78.0(3)
C9	C8	C15	C20	-101.8(3)
C8	C9	C10	C11	179.6(3)
C14	C9	C10	C11	-0.2(4)
C8	C9	C14	C13	-179.1(3)
C10	C9	C14	C13	0.7(4)
C9	C10	C11	C12	-0.6(5)
C10	C11	C12	O1	179.9(3)
C10	C11	C12	C13	0.9(4)
O1	C12	C13	C14	-179.3(3)
C11	C12	C13	C14	-0.4(4)
C12	C13	C14	C9	-0.5(5)
C8	C15	C16	C17	178.4(3)
C20	C15	C16	C17	-1.8(4)
C8	C15	C20	C19	-179.1(3)
C16	C15	C20	C19	1.1(4)
C15	C16	C17	C18	1.3(4)

Atom	Atom	Atom	Atom	Angle/°
C16	C17	C18	C19	-0.1(4)
C16	C17	C18	C22	-175.1(3)
C17	C18	C19	C20	-0.6(4)
C22	C18	C19	C20	174.4(3)
C17	C18	C22	F1	-42.3(4)
C17	C18	C22	F2	74.2(4)
C17	C18	C22	F3	-163.5(3)
C19	C18	C22	F1	142.8(3)
C19	C18	C22	F2	-100.8(3)
C19	C18	C22	F3	21.6(4)
C18	C19	C20	C15	0.0(5)

Table 6: Hydrogen Fractional Atomic Coordinates (×10^4) and Equivalent Isotropic Displacement Parameters (Å^2×10^3) for **I**. U_{eq} is defined as 1/3 of the trace of the orthogonalised U_{ij}.

Atom	x	y	z	U_{eq}
H1c14	1178.87	7751.81	1888.73	45.9
H1c13	1265.18	9250.9	882.65	46.5
H1c11	4374.75	3950.38	570.01	49.9
H1c10	4234.73	2440.21	1572.39	47.5
H1c21	2132.09	9799.43	-647.65	66.1
H2c21	633.73	9222.14	-261.35	66.1
H3c21	2401.95	10674.33	71.42	66.1
H1c20	6037.32	3075.54	3266.82	50.2
H1c8	3123.49	2438.36	2513.69	43.6
H1c17	3887.34	9753.12	3952	46.5
H1c16	2021.73	7860.24	3048.18	46.6
H1c19	7918.36	4974.43	4148.34	51.6
H1c7	-2352.53	-2462.68	1579.01	64.1
H2c7	-4115.1	-924.6	1524.49	64.1
H3c7	-2650.17	-356.63	1123.77	64.1
H1c1	-44.51	5025.48	2501.79	45.6
H1c6	1260.8	3448.59	3591.85	48.1
H1c5	-529.06	563.71	3747.18	54.7
H1c2	-540.27	2468.12	1571.32	51
H1c4	-3258.85	-123.26	2781.14	60.7
H2c4	-1531.71	-2003.24	2821.93	60.7

Table 7: Hydrogen Bond information for **I**.

D	H	A	d(D-H)/Å	d(H-A)/Å	d(D-A)/Å	D-H-A/deg
C6	H1c6	F2[1]	0.9600	2.5400	3.410(4)	151.00

––––

[1]1-x,-1/2+y,1-z

Table 8: Selected Bond Lengths in Å for **I**.

Atom	Atom	Length/Å
F1	C22	1.328(4)
F2	C22	1.348(4)
F3	C22	1.319(4)

Atom	Atom	Length/Å
O1	C12	1.374(4)
O1	C21	1.419(5)

Table 9: Selected Bond Angles in ° for **I**.

**Atom**	**Atom**	**Atom**	**Angle/°**
C12	O1	C21	116.9(2)
O1	C12	C11	116.6(3)
O1	C12	C13	124.5(3)
F1	C22	F2	103.4(3)
F1	C22	F3	106.5(3)
F1	C22	C18	112.9(2)
F2	C22	F3	107.6(3)
F2	C22	C18	112.3(3)
F3	C22	C18	113.5(3)

7. References

1.a) A. Ghanem, M. G. Gardiner, R. M. Williamson, P. Muller, Chem. Eur. J. 2010, 16, 3291; b) H. Tsutsui, T. Abe, S. Nakamura, M. Anada, S. Hashimoto, Chem Pharm Bull (Tokyo) 2005, 53, 1366; c) R. P. Reddy, H. M. Davies, Org. Lett. 2006, 8, 5013; d) R. P. Reddy, G. H. Lee, H. M. Davies, Org. Lett. 2006, 8, 3437; e) J. Fu, Z. Ren, J. Bacsa, D. G. Musaev, H. M. L. Davies, Nature 2018, 564, 395; f) C. Qin, H. M. Davies, J. Am. Chem. Soc. 2014, 136, 9792; g) H. M. L. Davies, P. R. Bruzinski, D. H. Lake, N. Kong, M. J. Fall, J. Am. Chem. Soc. 1996, 118, 6897; h) W. Liu, Z. Ren, A. T. Bosse, K. Liao, E. L. Goldstein, J. Bacsa, D. G. Musaev, B. M. Stoltz, H. M. L. Davies, J. Am. Chem. Soc. 2018, 140, 12247.

2. a.) Lee, M.; Ren, Z.; Musaev, D. G.; Davies, H. M. L., Rhodium-Stabilized Diarylcarbenes Behaving as Donor/Acceptor Carbenes. *ACS Catalysis* **2020,** *10* , 6240-6247. b.) Yang, L. L.; Evans, D.; Xu, B.; Li, W. T.; Li, M. L.; Zhu, S. F.; Houk, K. N.; Zhou, Q. L., Enantioselective Diarylcarbene Insertion into Si-H Bonds Induced by Electronic Properties of the Carbenes. *J. Am. Chem. Soc.* **2020,** *142*, 12394-12399. C.) Liu, W. B.; Twilton, J.; Wei, B.; Lee, M.; Hopkins, M. N.; Bacsa, J.; Stahl, S. S.; Davies, H. M. L., Copper-Catalyzed Oxidation of Hydrazones to Diazo Compounds Using Oxygen as the Terminal Oxidant. *ACS Catalysis* **2021,** *11*, 2676-2683.

3. Nicolle, S. M.; Moody, C. J. Potassium N-iodo p-toluenesulfonamide (TsNIK, Iodamine-T): a new reagent for the oxidation of hydrazones to diazo compounds. *Chem. Eur. J.* **2014,** *20*, 4420.

4. Yu, J.-Y.; Kuwano, R., Suzuki-Miyaura Coupling of Diarylmethyl Carbonates with Arylboronic Acids: A New Access to Triarylmethanes. *Org. Lett.* **2008,** *10*, 973-976.

5.CrysAlisPro (Rigaku, V1.171.41.98a, 2021)

6. CrysAlisPro (ROD), Rigaku Oxford Diffraction, Poland.

8. HPLC Spectra for Enantioselectivity Determination

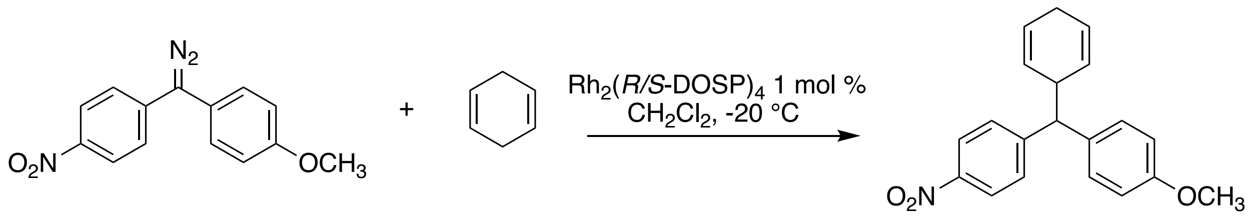

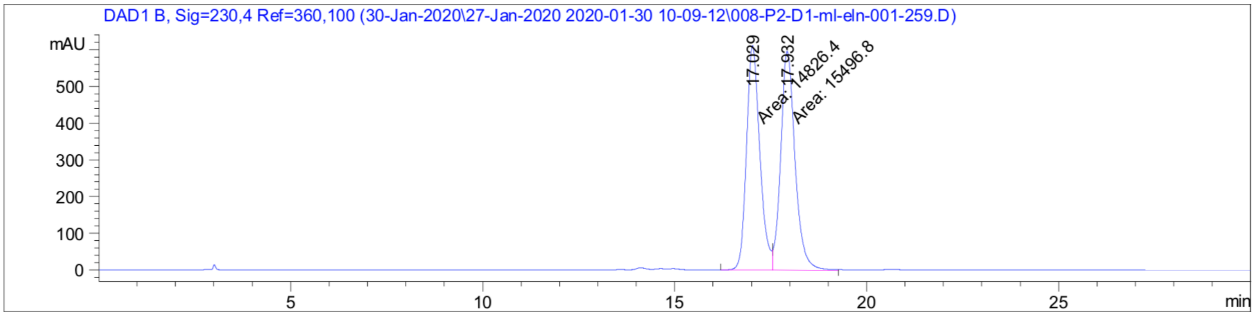

Si gnal 2: DAD1 B, Si g=230, 4 Ref =360, 100

Peak #	Ret Ti me [mi n]	Type	Wi dt h [mi n]	Ar ea [mAU* s]	Hei ght [mAU]	Ar ea %
1	17. 029	MF	0. 4038	1. 48264e4	611. 92981	48. 8946
2	17. 932	FM	0. 4331	1. 54968e4	596. 41248	51. 1054
Tot al s :				3. 03232e4	1208. 34229	

HPLC (ADH column, 1.0 mL/min 1% i-PrOH in n-hexane 30 min, UV 230 nm)

Compound 3 Table 1 catalyst Screen.

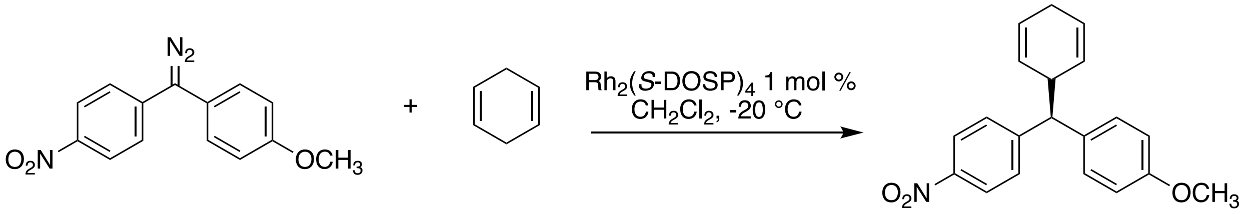

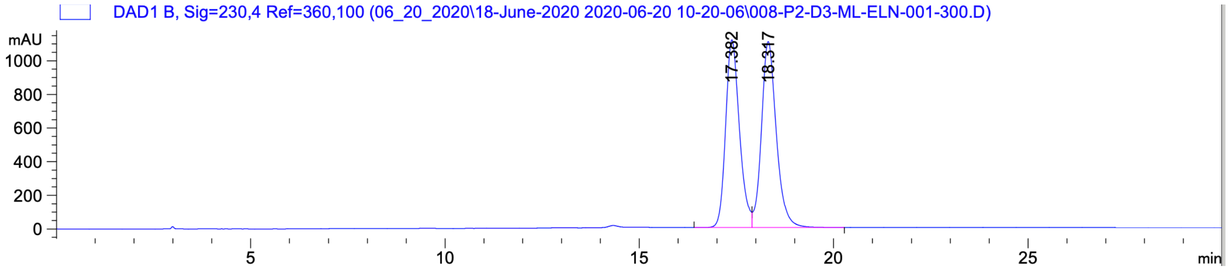

Signal 2: DAD1 B, Sig=230,4 Ref=360,100

Peak #	RetTime [min]	Type	Width [min]	Area [mAU*s]	Height [mAU]	Area %
1	17.382	BV	0.3794	2.76866e4	1116.22778	48.3530
2	18.317	VB	0.4061	2.95726e4	1105.29822	51.6470
Totals :				5.72592e4	2221.52600	

HPLC (ADH column, 1.0 mL/min 1% i-PrOH in n-hexane 30 min, UV 230 nm) retention times of 17.38 (minor) and 18.32 min (major) 3 % ee with Rh_2(*S*-DOSP)$_4$.

Compound 3 Table 1 catalyst Screen

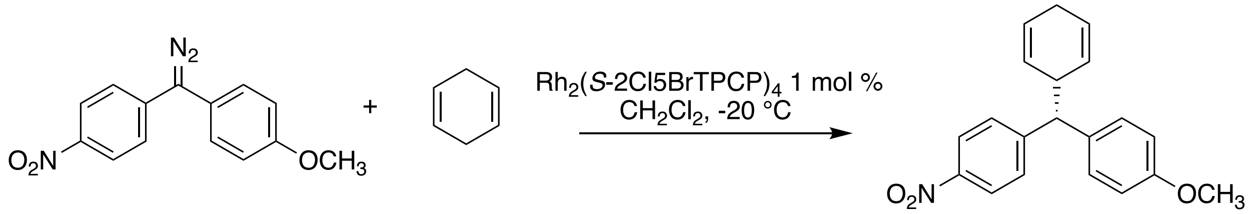

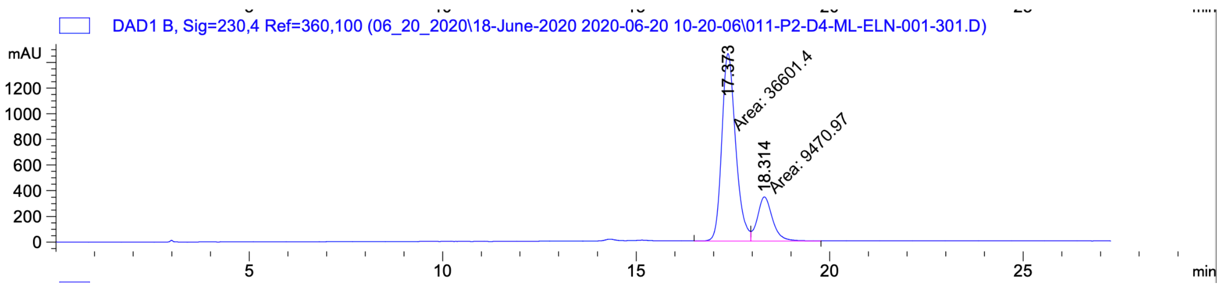

Signal 2: DAD1 B, Sig=230,4 Ref=360,100

Peak #	RetTime [min]	Type	Width [min]	Area [mAU*s]	Height [mAU]	Area %
1	17.373	MF	0.4172	3.66014e4	1462.27087	79.4433
2	18.314	FM	0.4577	9470.96973	344.90491	20.5567
Totals :				4.60724e4	1807.17578	

HPLC (ADH column, 1.0 mL/min 1% i-PrOH in n-hexane 30 min, UV 230 nm) retention times of 17.37 (minor) and 18.31 min (major) -79 % ee with Rh_2(*S*-2CL5BrTPCP)$_4$.

Compound 3 Table 1 catalyst Screen

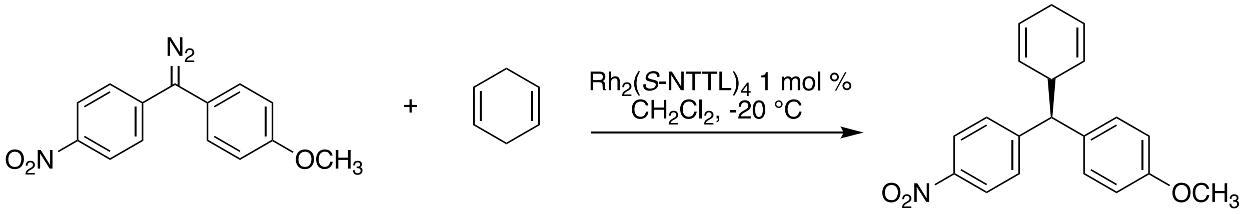

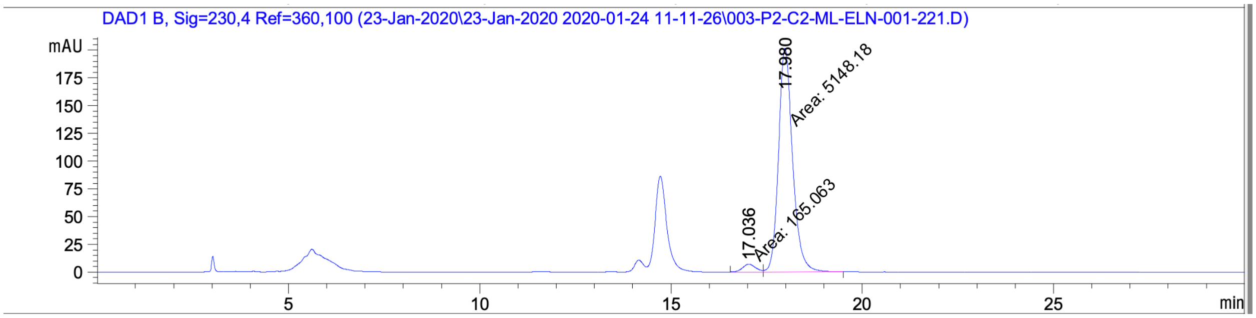

Signal 2: DAD1 B, Sig=230,4 Ref=360,100

Peak #	RetTime [min]	Type	Width [min]	Area [mAU*s]	Height [mAU]	Area %
1	17.036	MF	0.3915	165.06316	7.02773	3.1066
2	17.980	FM	0.4257	5148.18408	201.54683	96.8934
Totals :				5313.24724	208.57456	

HPLC (ADH column, 1.0 mL/min 1% i-PrOH in n-hexane 30 min, UV 230 nm) retention times of 17.1(minor) and 18.0min (major) 94 % ee with Rh_2(*S*-TPPTTL)$_4$.

Compound 3 Table 1 catalyst Screen

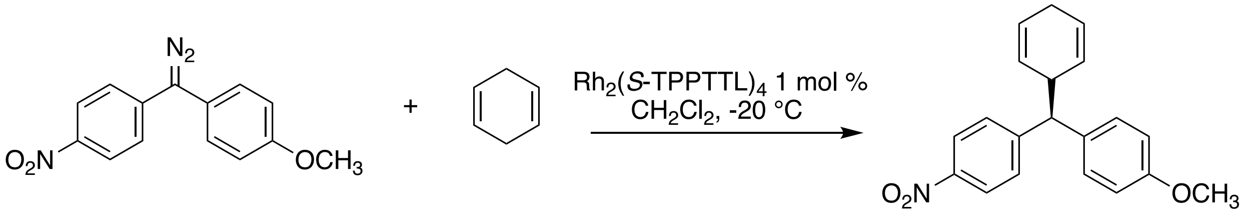

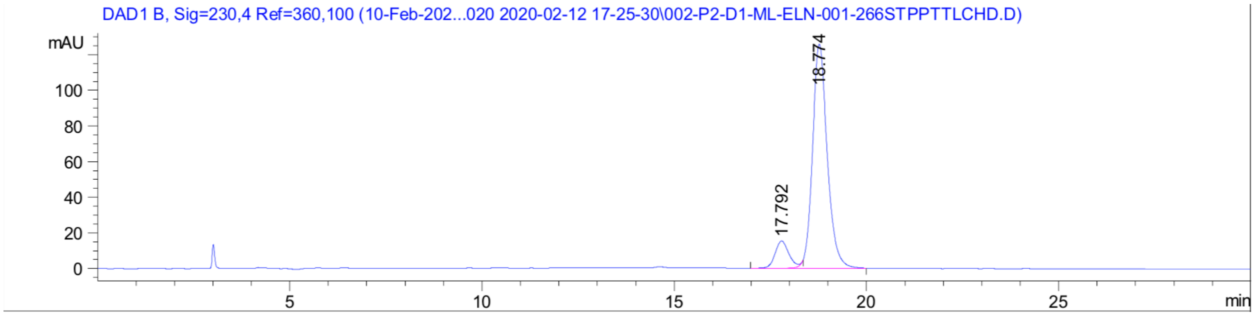

Signal 2: DAD1 B, Sig=230, 4 Ref =360, 100

Peak #	RetTime [min]	Type	Width [min]	Area [mAU*s]	Height [mAU]	Area %
1	17.792	BV E	0.3768	378.00079	15.27041	10.1628
2	18.774	VB R	0.4041	3341.45801	125.69870	89.8372

Totals : 3719.45880 140.96911

HPLC (ADH column, 1.0 mL/min 1% i-PrOH in n-hexane 30 min, UV 230 nm) retention times of 17.80 (minor) and 18.77 min (major) 79 % ee with Rh_2(*S*-TPPTTL)$_4$.

Compound 3 Table 1 catalyst Screen

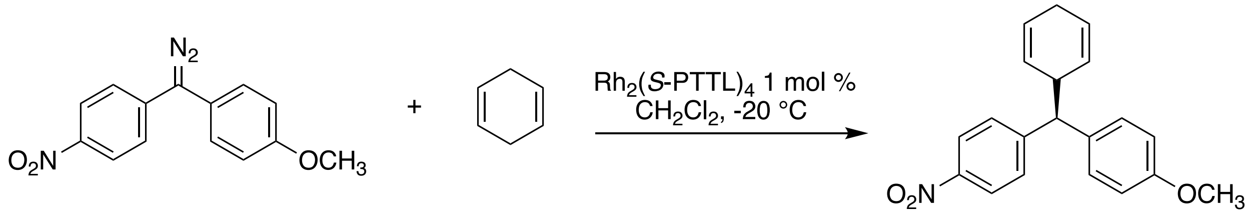

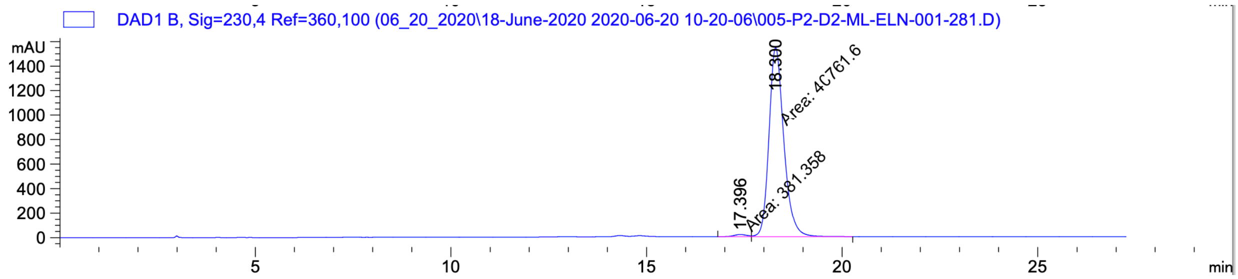

Signal 2: DAD1 B, Sig=230,4 Ref=360,100

Peak #	RetTime [min]	Type	Width [min]	Area [mAU*s]	Height [mAU]	Area %
1	17.396	MF	0.3778	381.35757	16.82251	0.9269
2	18.300	FM	0.4406	4.07616e4	1541.95007	99.0731
Totals :				4.11430e4	1558.77259	

HPLC (ADH column, 1.0 mL/min 1% i-PrOH in n-hexane 30 min, UV 230 nm) retention times of 17.40 (minor) and 18.3 min (major) 98 % ee with Rh_2(*S*-PTTL)$_4$.

Compound 3 Table 1 catalyst Screen

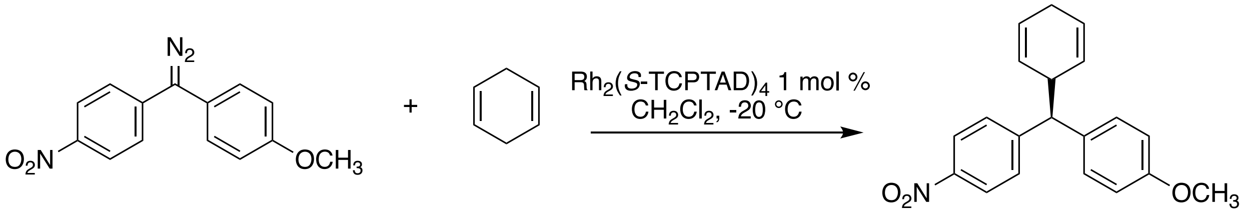

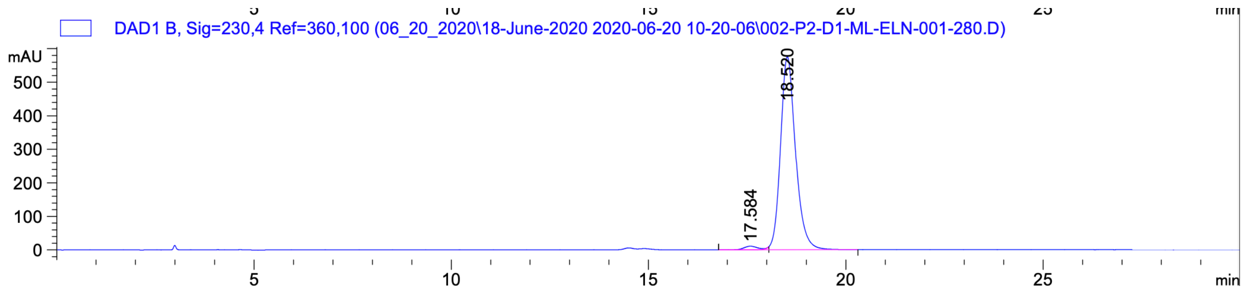

Signal 2: DAD1 B, Sig=230,4 Ref=360,100

Peak #	RetTime [min]	Type	Width [min]	Area [mAU*s]	Height [mAU]	Area %
1	17.584	BV E	0.3710	262.74722	10.83318	1.6928
2	18.520	VB R	0.4034	1.52584e4	575.31097	98.3072
Totals :				1.55211e4	586.14416	

HPLC (ADH column, 1.0 mL/min 1% i-PrOH in n-hexane 30 min, UV 230 nm) retention times of 17.58 (minor) and 18.52 min (major) 97 % ee with Rh_2(*S*-TCPTAD)$_4$.

Compound 3 Table 1 catalyst Screen

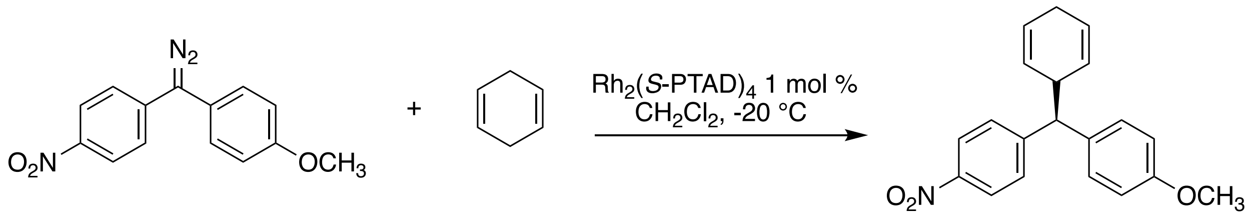

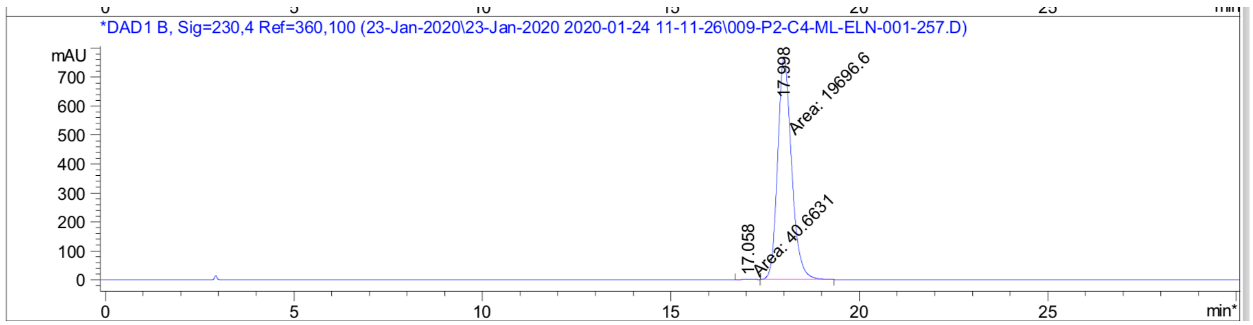

Peak #	RetTime [min]	Type	Width [min]	Area [mAU*s]	Height [mAU]	Area %
1	17.058	MM	0.3362	40.66310	2.01585	0.2060
2	17.998	MM	0.4271	1.96966e4	768.57556	99.7940

Totals : 1.97372e4 770.59141

HPLC (ADH column,1.0 mL/min 1% i-PrOH in n-hexane 30 min, UV 230 nm) retention times of 17.06 (minor) and 18.00 min (major) 99 % ee with Rh_2(*S*-PTAD)$_4$.

Compound 4-Racemic

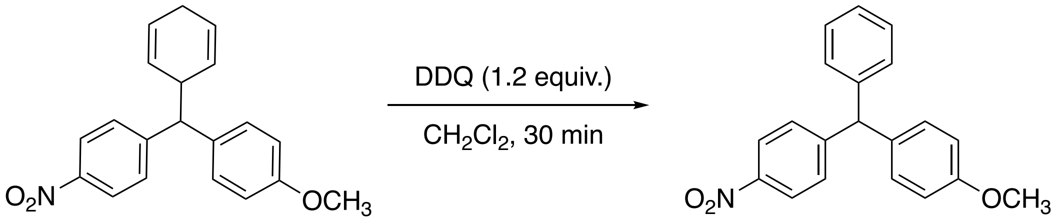

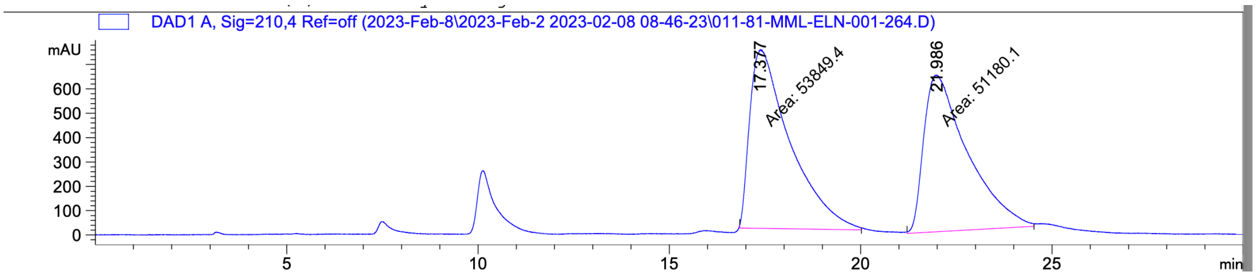

Peak #	RetTime [min]	Type	Width [min]	Area [mAU*s]	Height [mAU]	Area %
1	17.390	MM	1.2610	2.61256e4	345.30624	50.7963
2	21.995	MM	1.4203	2.53064e4	296.96356	49.2037
Totals :				5.14320e4	642.26981	

Compound 4

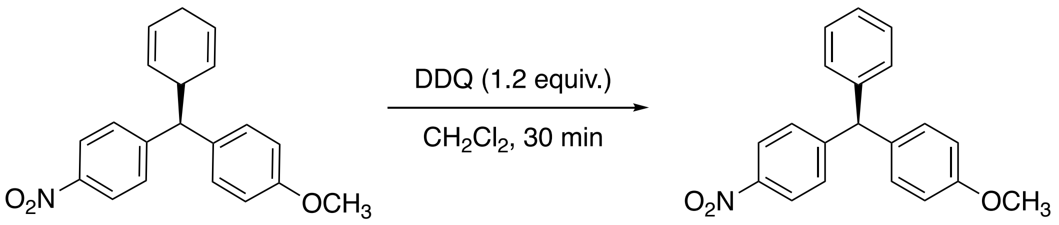

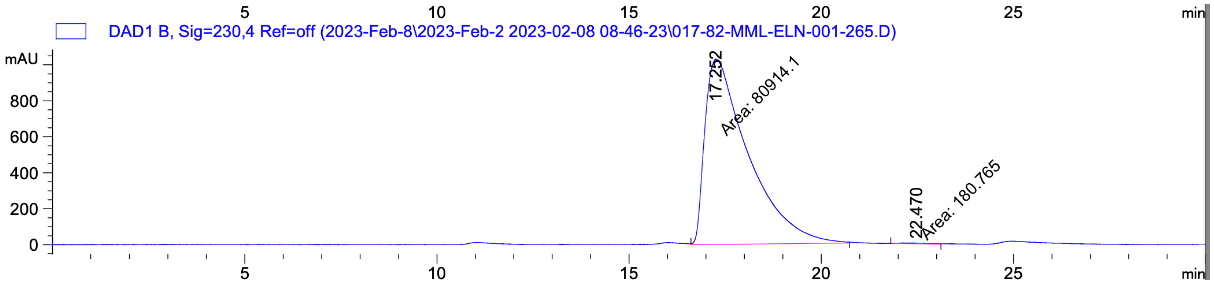

Signal 2: DAD1 B, Sig=230,4 Ref=off

Peak #	RetTime [min]	Type	Width [min]	Area [mAU*s]	Height [mAU]	Area %
1	17.252	MM	1.3081	8.09141e4	1030.96216	99.7771
2	22.470	MM	0.8065	180.76530	3.73546	0.2229
Totals :				8.10949e4	1034.69762	

HPLC (OD column,1.0 mL/min 1% i-PrOH in n-hexane 30 min, UV 230 nm) retention times of 17.3 (major) and 22.5 min (minor) 99 % ee with Rh_2(*S*-PTAD)$_4$.

Compound 4 1mmol scale

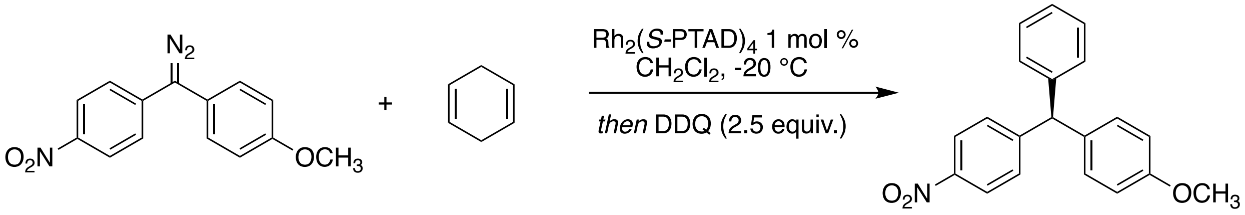

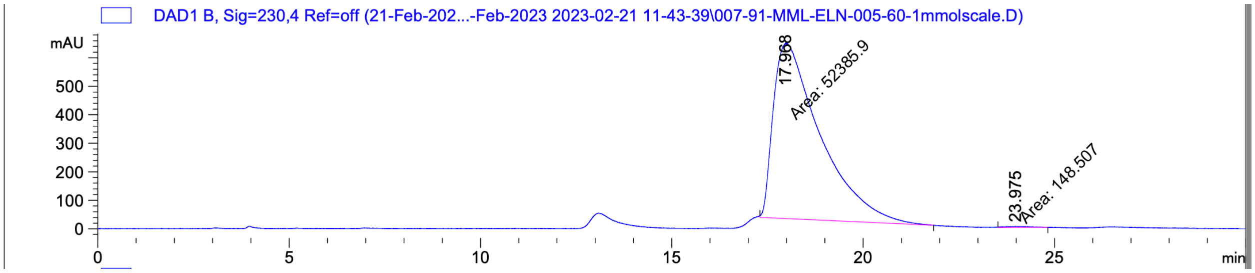

Peak #	RetTime [min]	Type	Width [min]	Area [mAU*s]	Height [mAU]	Area %
1	17.968	MM	1.4183	5.23859e4	615.59192	99.7173
2	23.975	MM	0.6302	148.50720	3.92752	0.2827

Totals :				5.25345e4	619.51944	

Compound 5-Racemic

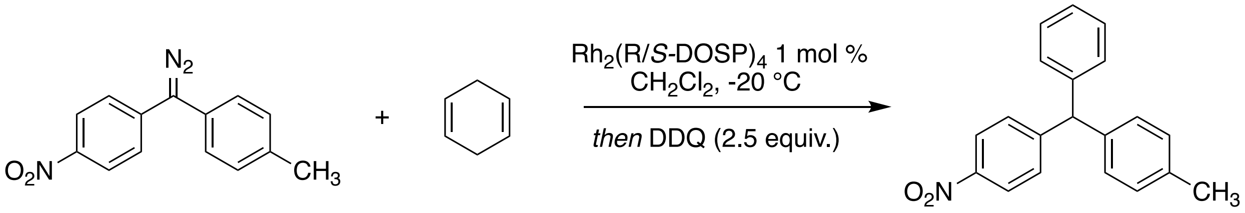

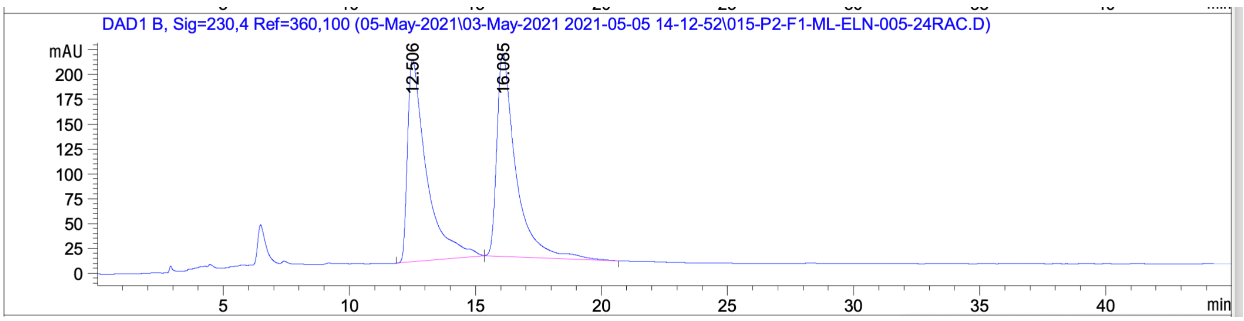

Signal 2: DAD1 B, Sig=230,4 Ref=360,100

Peak #	RetTime [min]	Type	Width [min]	Area [mAU*s]	Height [mAU]	Area %
1	12.506	BB	0.7440	1.06622e4	203.26964	49.9128
2	16.085	BB	0.7555	1.06995e4	204.14397	50.0872
Totals :				2.13617e4	407.41360	

HPLC (ODH column, 1.0 mL/min 0.5% i-PrOH in n-hexane 45 min, UV 230 nm) retention times of 12.51 (major) and 16.09 min (minor) 0 % ee with Rh_2(*R/*S-DOSP)$_4$.

Compound 5-Chiral

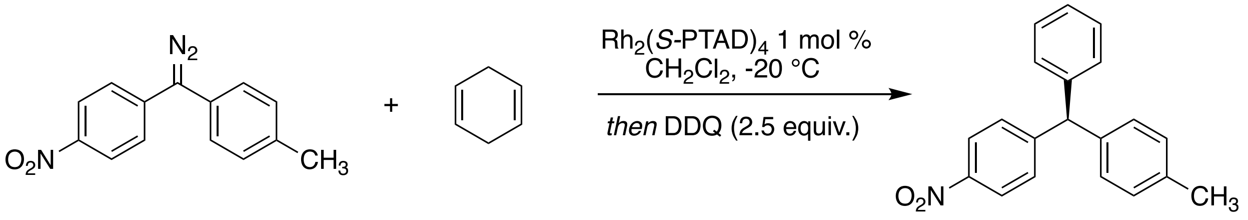

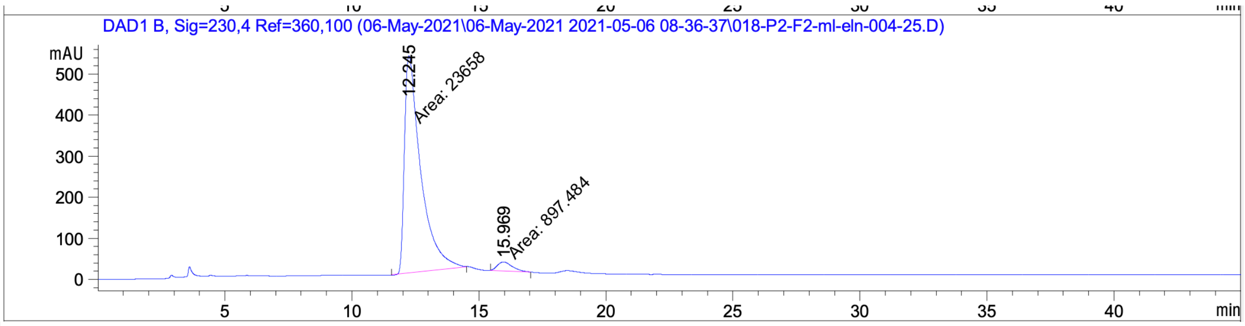

Signal 2: DAD1 B, Sig=230,4 Ref=360,100

Peak #	RetTime [min]	Type	Width [min]	Area [mAU*s]	Height [mAU]	Area %
1	12.245	MM	0.7448	2.36580e4	529.37201	96.3451
2	15.969	MM	0.6764	897.48407	22.11265	3.6549
Totals :				2.45555e4	551.48466	

HPLC (ODH column, 1.0 mL/min 0.5 % i-PrOH in n-hexane 45 min, UV 230 nm) retention times of 12.25 (major) and 15.97 min (minor) 93 % ee with Rh_2(S-PTAD)$_4$.

Compound 6-Racemic

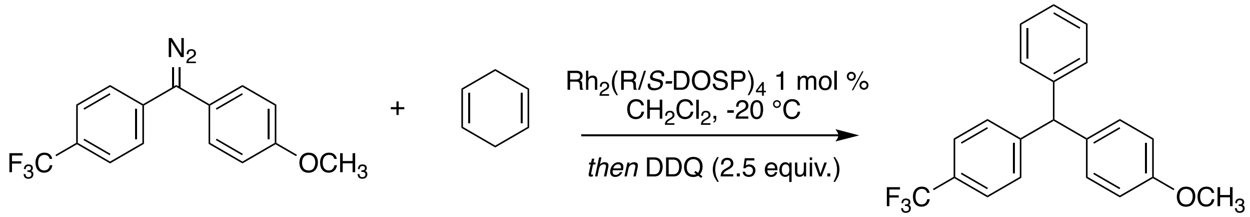

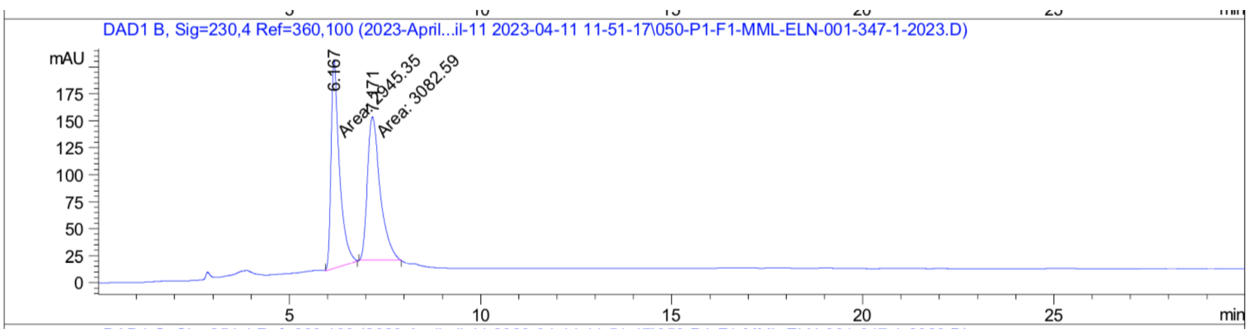

Si gnal 2: DAD1 B, Si g=230, 4 Ref =360, 100

Peak #	Ret Ti me [mi n]	Type	Wi dt h [mi n]	Ar ea [mAU*s]	Hei ght [mAU]	Ar ea %
1	6. 167	MM	0. 2535	2945. 35327	193. 61526	48. 8617
2	7. 171	MM	0. 3869	3082. 59058	132. 77647	51. 1383
Tot al s :				6027. 94385	326. 39174	

HPLC (ODH column1.0 mL/min 0.5% *i*-PrOH in *n*-hexane 30 min, UV 230 nm) retention times of 6.2 and 7.2min 0 % ee with Rh_2(*R/*S-DOSP)$_4$.

Compound 6-Chiral

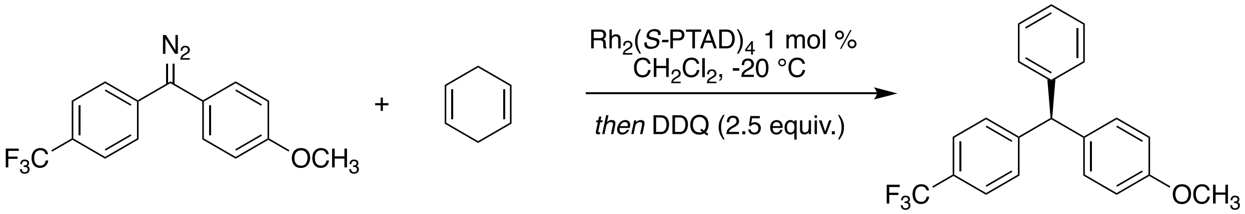

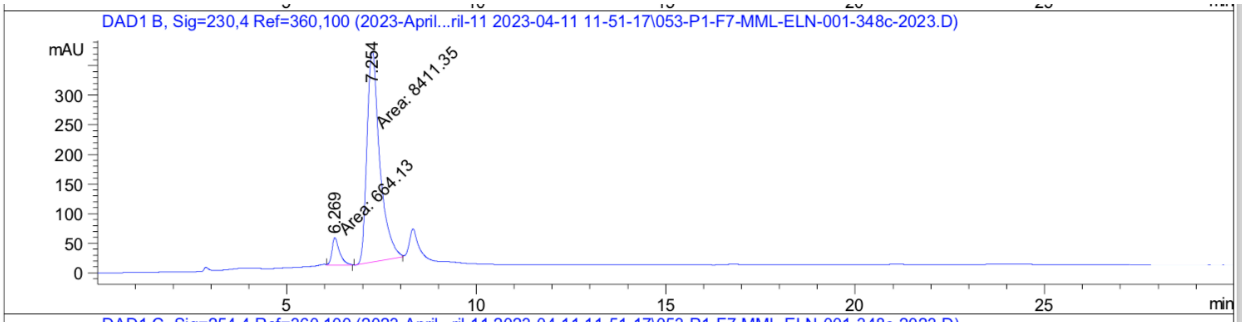

Signal 2: DAD1 B, Sig=230,4 Ref=360,100

Peak #	RetTime [min]	Type	Width [min]	Area [mAU*s]	Height [mAU]	Area %
1	6.269	MM	0.2369	664.13013	46.72340	7.3179
2	7.254	MM	0.3939	8411.34668	355.90387	92.6821
Totals :				9075.47681	402.62727	

HPLC (ODH column, hexane, 1.0 mL/min 0.5% *i*-PrOH in *n*-hexane 25 min, UV 230 nm) retention times of 6.2 (minor) and 7.2 min (major) 85 % ee with Rh_2(S-PTAD)$_4$.

Compound 7-Racemic

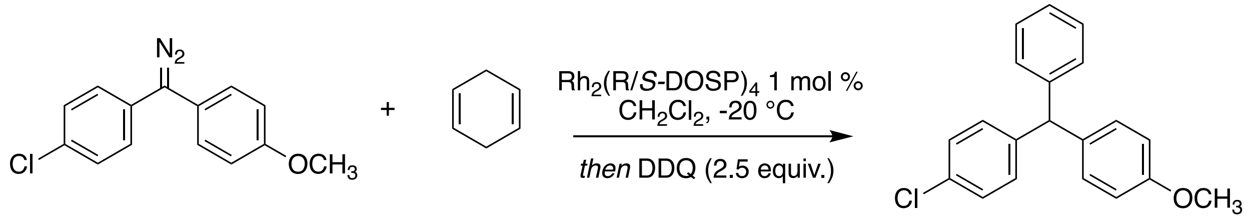

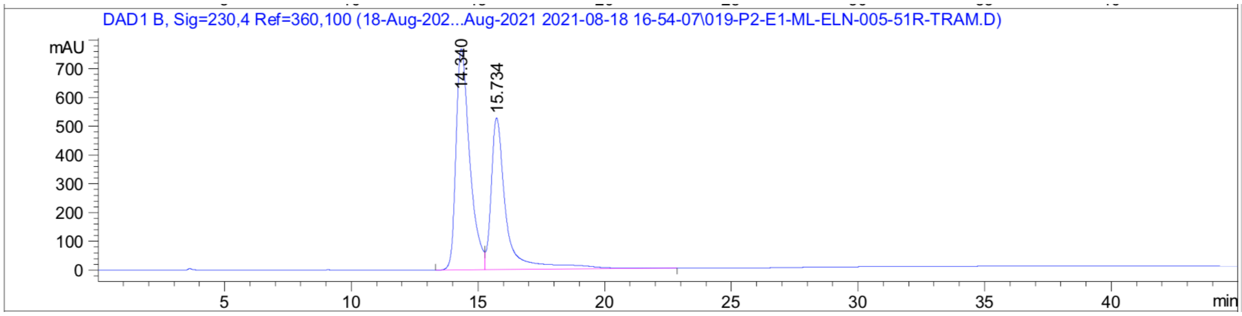

Signal 2: DAD1 B, Sig=230,4 Ref=360,100

Peak #	RetTime [min]	Type	Width [min]	Area [mAU*s]	Height [mAU]	Area %
1	14.340	BV	0.5670	2.90592e4	768.75085	56.6860
2	15.734	VB	0.6112	2.22043e4	527.37427	43.3140

Totals : 5.12636e4 1296.12512

HPLC (ODH column 0.80 mL/min 0.2% i-PrOH in n-hexane 20 min, UV 230 nm) retention times of 14.3 and 15.7 min with Rh_2(*R*/S-DOSP)$_4$..

Compound 7-Chiral

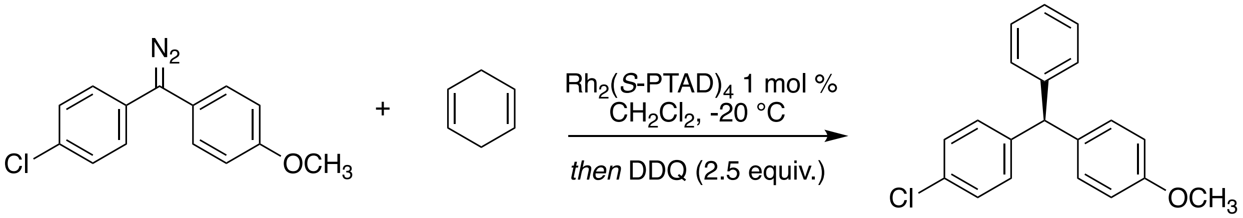

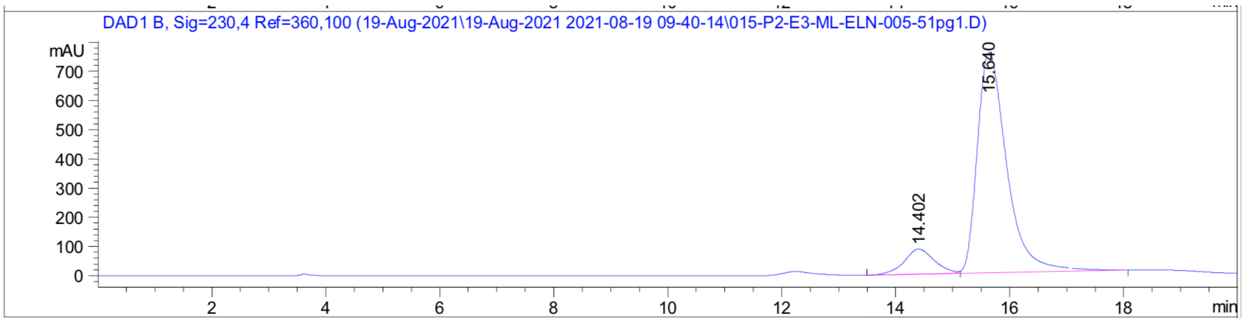

Signal 2: DAD1 B, Sig=230, 4 Ref =360, 100

Peak #	Ret Time [min]	Type	Width [min]	Area [mAU*s]	Height [mAU]	Area %
1	14.402	BV E	0.5514	3197.94141	86.48994	10.5457
2	15.640	VB R	0.5489	2.71266e4	755.56897	89.4543

Totals : 3.03246e4 842.05891

HPLC (ODH column, hexane, 0.8 mL/min 0.2% i-PrOH in n-hexane 20 min, UV 230 nm) retention times of 14.40 (minor) and 15.64 min (major) 79 % ee with Rh_2(S-PTAD)$_4$.

Compound 8 (C-H) intermediate -Racemic

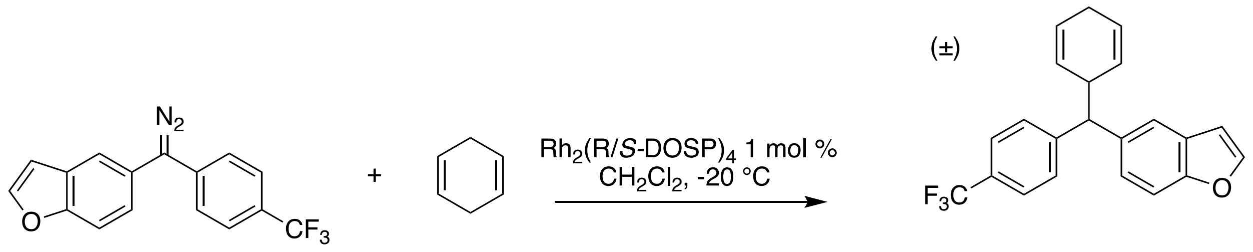

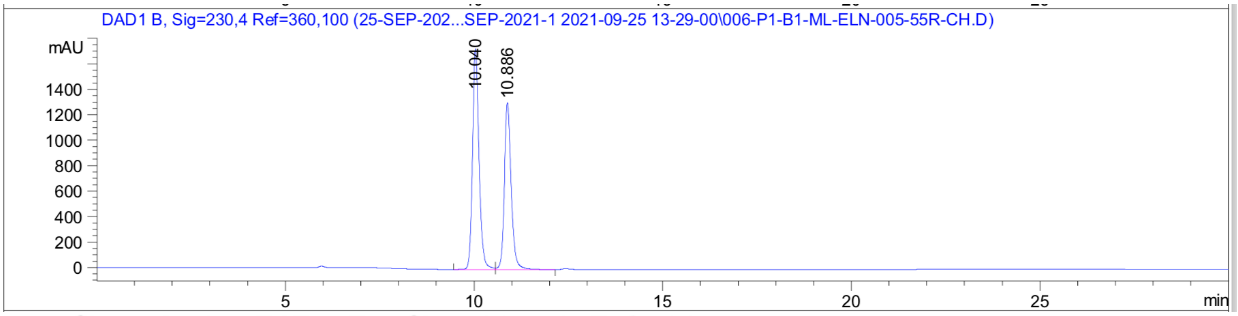

Signal 2: DAD1 B, Sig=230,4 Ref=360,100

Peak #	RetTime [min]	Type	Width [min]	Area [mAU*s]	Height [mAU]	Area %
1	10.040	VV R	0.1827	2.09414e4	1733.34412	55.9860
2	10.886	VB	0.1884	1.64633e4	1312.39636	44.0140

Totals : 3.74048e4 3045.74048

Compound 8(C-H) intermediate -Chiral

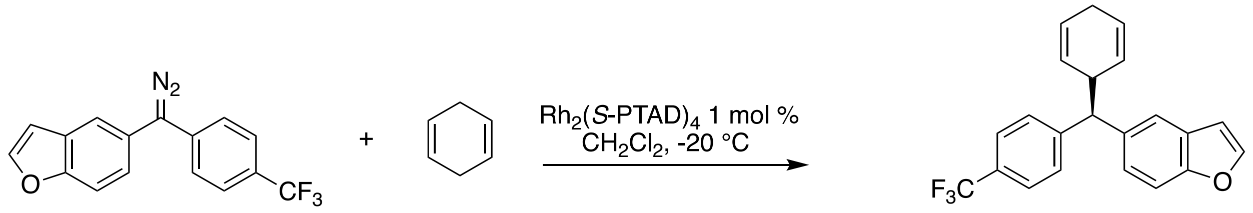

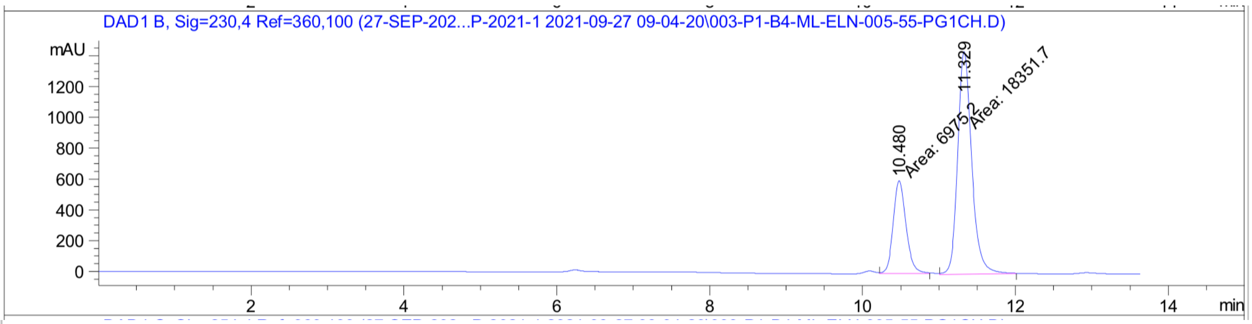

Signal 2: DAD1 B, Sig=230,4 Ref=360,100

Peak #	RetTime [min]	Type	Width [min]	Area [mAU*s]	Height [mAU]	Area %
1	10.480	MM	0.1932	6975.20166	601.83905	27.5407
2	11.329	MM	0.2114	1.83517e4	1446.64294	72.4593

Totals : 2.53269e4 2048.48199

Compound 9 (C-H) intermediate -Racemic

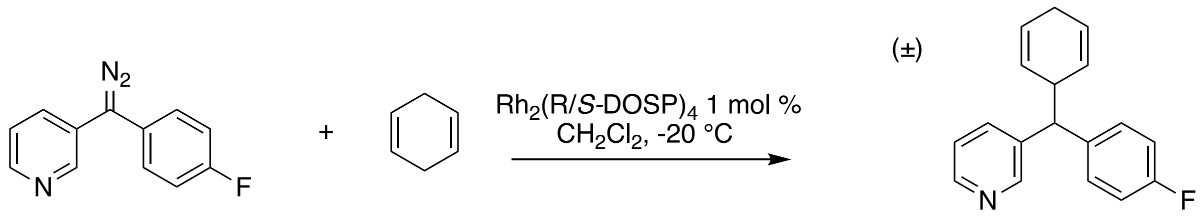

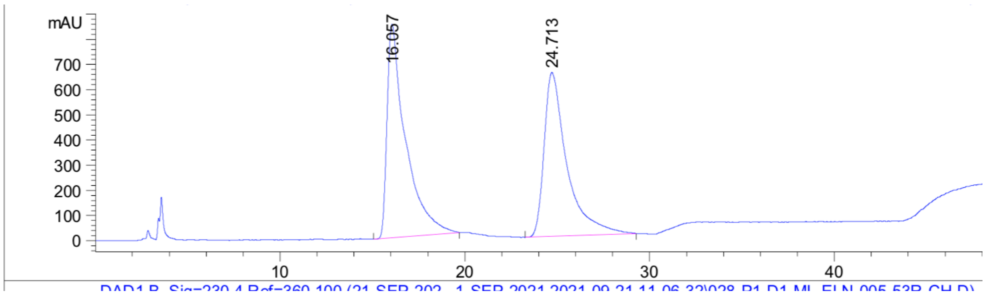

Signal 2: DAD1 B, Sig=230,4 Ref=360,100

Peak #	RetTime [min]	Type	Width [min]	Area [mAU*s]	Height [mAU]	Area %
1	16.057	BB	0.9639	1.82063e4	264.89426	50.8038
2	24.711	BB	1.2796	1.76302e4	204.14148	49.1962

1290 LC 9/22/2021 9:42:37 AM SYSTEM

Data File C:\Users\P...-2021\21-SEP-2021 2021-09-21 11-06-32\0
Sample Name: ML-ELN-005-53R-CH

Peak #	RetTime [min]	Type	Width [min]	Area [mAU*s]	Height [mAU]	Area %
Totals :				3.58366e4	469.03574	

Compound 9 (C-H) intermediate -Chiral

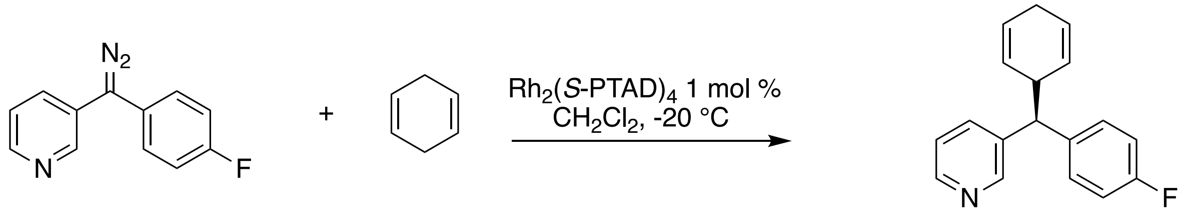

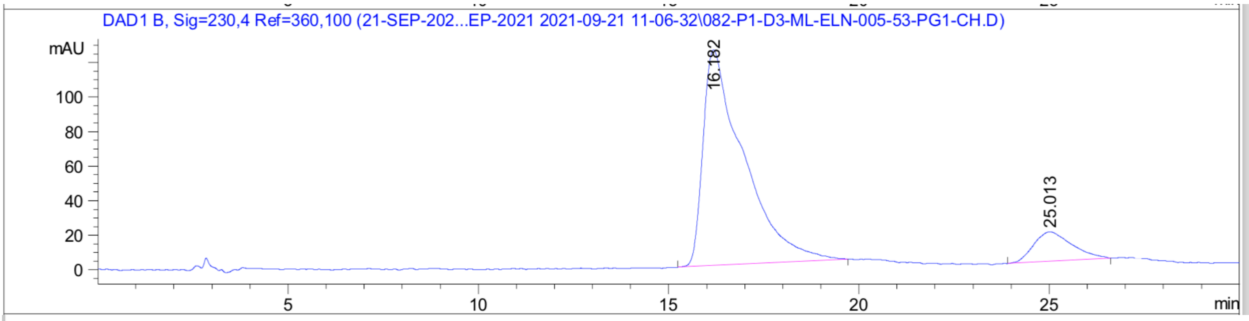

Signal 2: DAD1 B, Sig=230,4 Ref=360,100

Peak #	RetTime [min]	Type	Width [min]	Area [mAU*s]	Height [mAU]	Area %
1	16.182	BB	1.0178	9286.60059	124.84396	88.5410
2	25.013	BB	0.9458	1201.87012	17.10319	11.4590
Totals :				1.04885e4	141.94715	

HPLC (ADH column, 1.0 mL/min 2.25 % i-PrOH in n-hexane 30 min, UV 230 nm) retention times of 16.2 (major) and 25.03 min (minor) 77 % ee with Rh_2(S-PTAD)$_4$.

Compound 11 (C-H) intermediate -Racemic

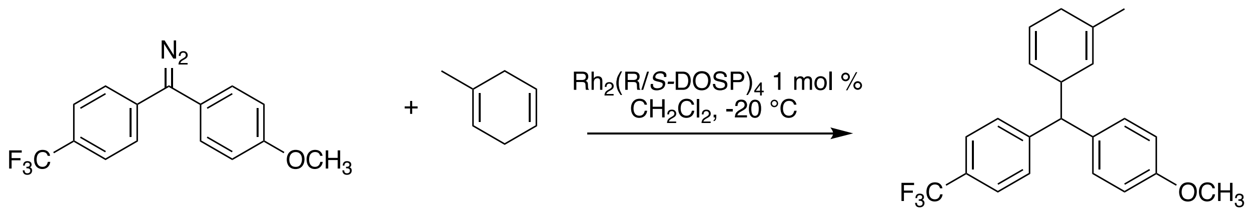

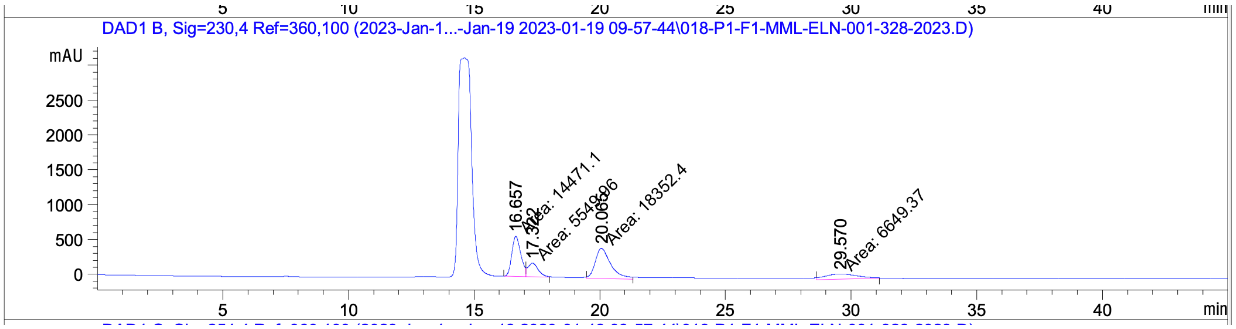

Signal 2: DAD1 B, Sig=230,4 Ref=360,100

Peak #	RetTime [min]	Type	Width [min]	Area [mAU*s]	Height [mAU]	Area %
1	16.657	MF	0.4187	1.44711e4	576.01093	32.1417
2	17.322	FM	0.4712	5549.96289	196.31697	12.3270
3	20.065	MM	0.7132	1.83524e4	428.88205	40.7624
4	29.570	MM	1.4402	6649.37451	76.95222	14.7689
Totals :				4.50229e4	1278.16217	

This trace tells us peaks 1 and 3 are the peaks corresponding to the major diastereomer and 2 and 4 are the other set peaks corresponding to the minor diastereomers.

Compound 11 (C-H) intermediate –Chiral

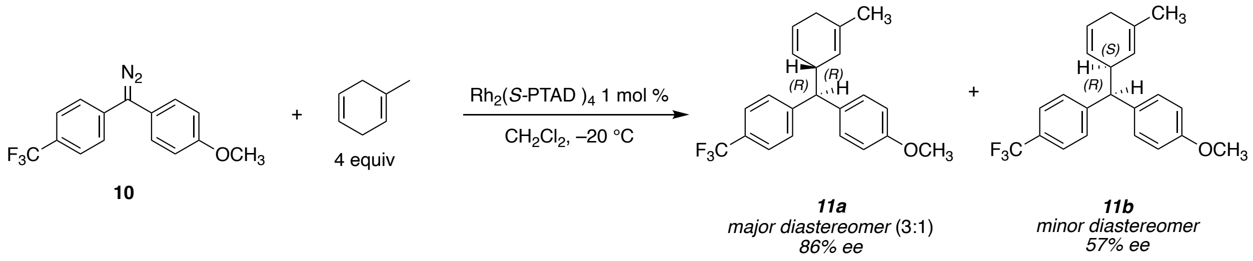

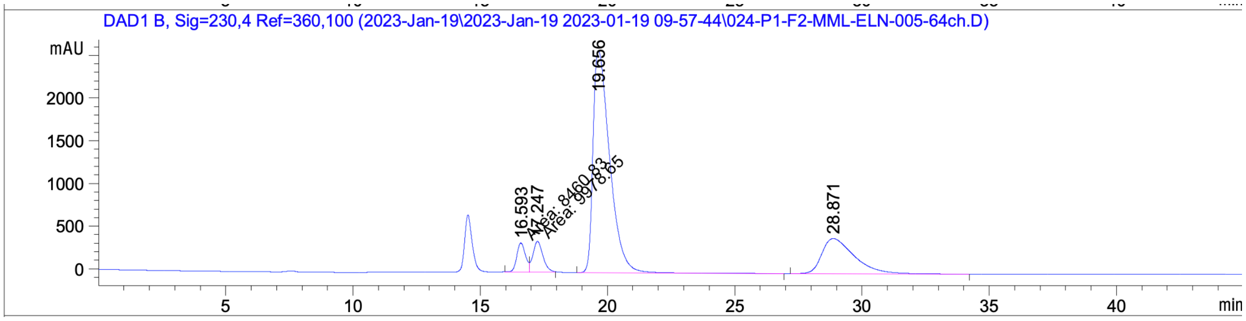

Signal 2: DAD1 B, Sig=230,4 Ref=360,100

Peak #	RetTime [min]	Type	Width [min]	Area [mAU*s]	Height [mAU]	Area %
1	16.593	MF	0.4102	8460.83008	343.75934	4.8815
2	17.247	FM	0.4557	9978.64746	364.95328	5.7572
3	19.656	BB	0.6851	1.18426e5	2601.37769	68.3262
4	28.871	BB	1.3181	3.64587e4	411.30997	21.0350
Totals :				1.73324e5	3721.40027	

Major Diasteriomer(**11a**): peak 3 and 1.
HPLC (ADH column, 0.4 mL/min 0.2 % *i*-PrOH in *n*-hexane 30 min, UV 230 nm) retention times of 16.6 (minor) and 19.7 min (major) 86 % ee with Rh_2(S-PTAD)$_4$.

Minor Diasteriomer(**11b**): peak 4 and 2.
HPLC (ADH column, 0.4 mL/min 0.2 % *i*-PrOH in *n*-hexane 30 min, UV 230 nm) retention times of 17.3 (minor) and28.9 min (major) 57 % ee with Rh_2(S-PTAD)$_4$.

Compound 12 -Racemic

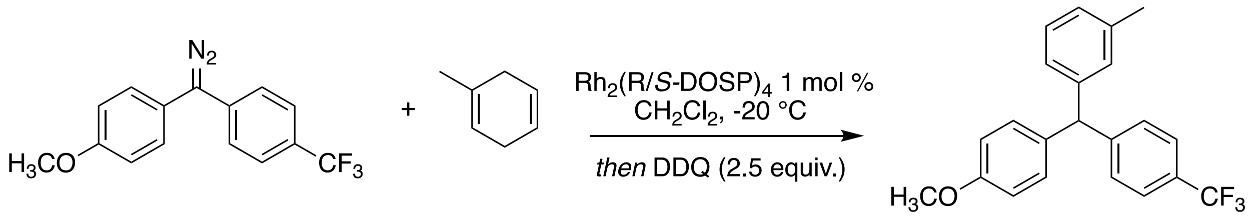

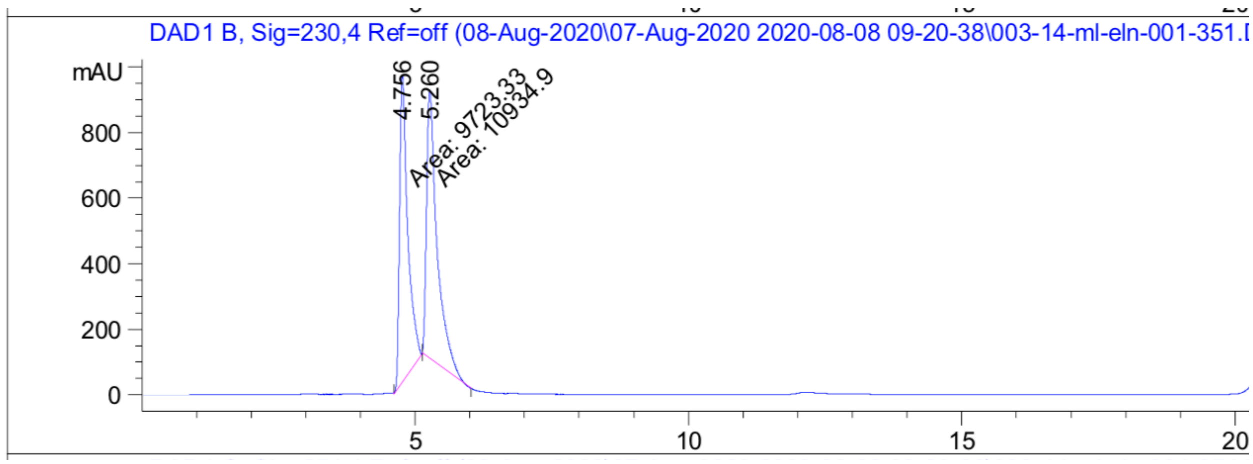

Signal 2: DAD1 B, Sig=230,4 Ref=off

Peak #	RetTime [min]	Type	Width [min]	Area [mAU*s]	Height [mAU]	Area %
1	4.756	MM	0.1732	9723.33008	935.85950	47.0675
2	5.260	MM	0.2226	1.09349e4	818.84296	52.9325
Totals :				2.06583e4	1754.70245	

HPLC (ODH column, 1.0 mL/min 1% i-PrOH in n-hexane 25 min, UV 230 nm) retention times of 4.76 and 5.26 min with Rh_2(*R/*S-DOSP)$_4$.

Compound 12 -Chiral

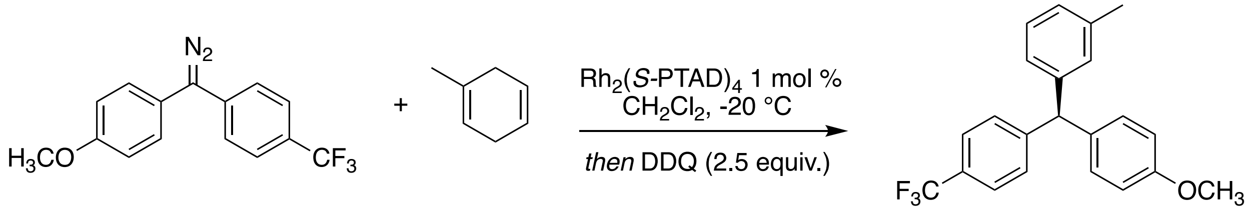

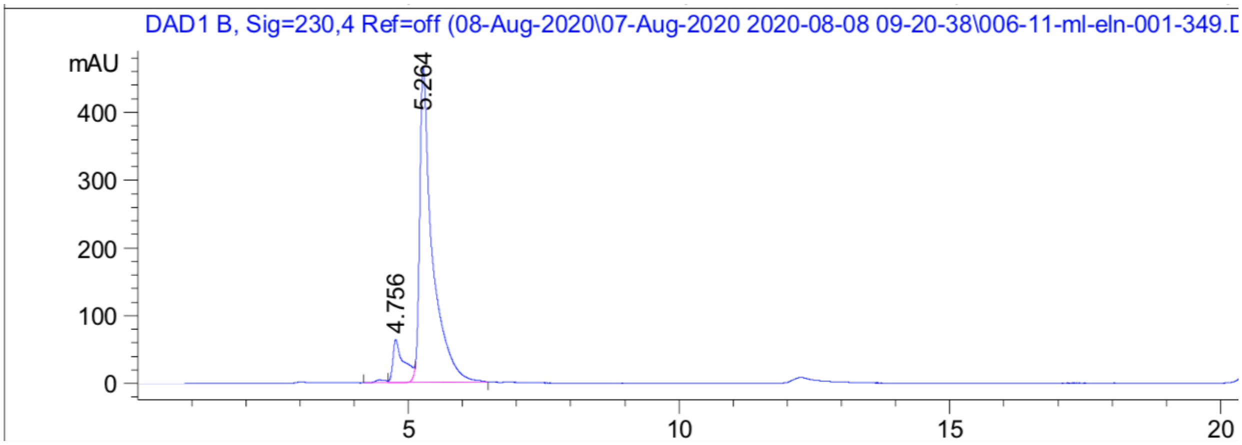


```
Signal 2: DAD1 B, Sig=230,4 Ref=off
```

Peak #	RetTime [min]	Type	Width [min]	Area [mAU*s]	Height [mAU]	Area %
1	4.756	VV E	0.1864	897.16034	63.45586	10.4860
2	5.264	VV R	0.2241	7658.66748	466.45120	89.5140

Totals : 8555.82782 529.90706

HPLC (ODH column, 1.0 mL/min 1% i-PrOH in n-hexane 25 min, UV 230 nm) retention times of 4.76 (minor) and 5.26 min (major) 79 % ee with Rh_2(S-PTAD)$_4$.

Compound 13 -Racemic

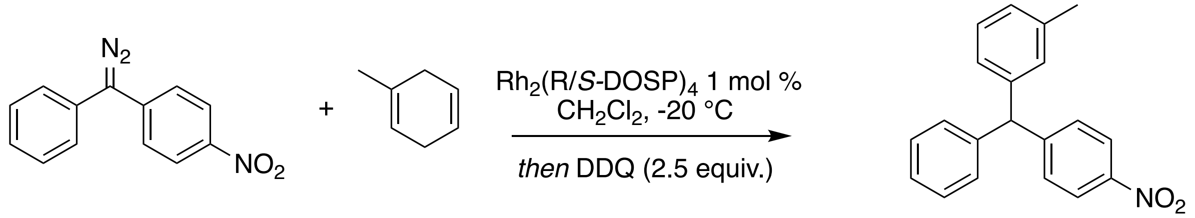

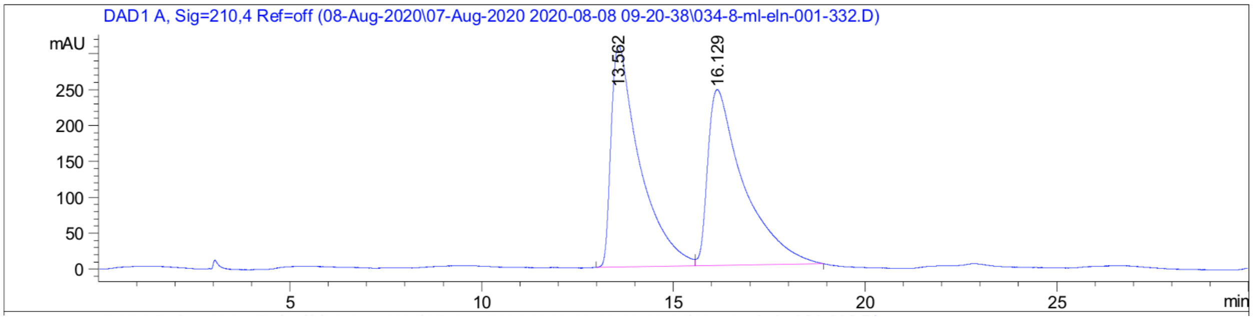

Signal 1: DAD1 A, Sig=210,4 Ref=off

Peak #	RetTime [min]	Type	Width [min]	Area [mAU*s]	Height [mAU]	Area %
1	13.562	VV R	0.6122	1.60869e4	308.32913	50.0392
2	16.129	VV R	0.7686	1.60617e4	245.07031	49.9608
Totals :				3.21485e4	553.39944	

HPLC (OD column, 1.0 mL/min 0.5 % i-PrOH in n-hexane 30 min, UV 210 nm) retention times of 13.56 (major) and 16.13 min (minor) 0 % ee with Rh_2(*R/*S-DOSP)$_4$.

Compound 13 -Chiral

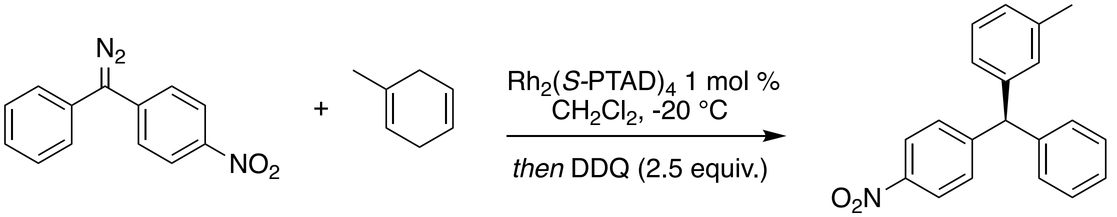

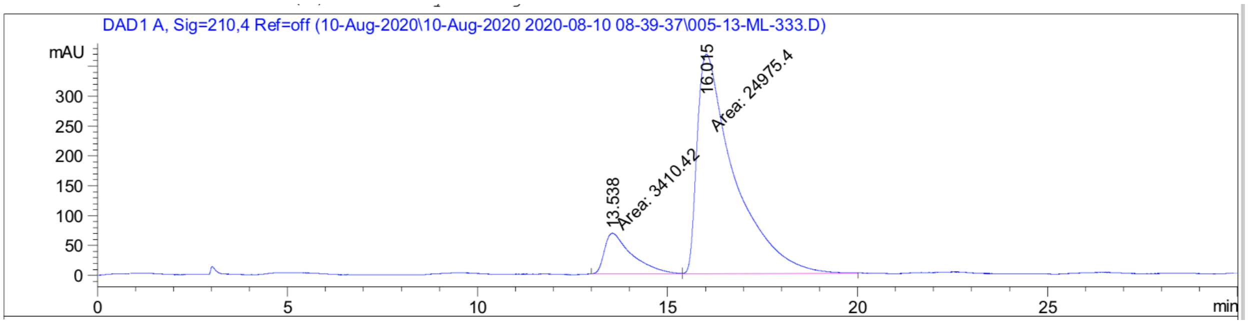

Signal 1: DAD1 A, Sig=210,4 Ref=off

Peak #	RetTime [min]	Type	Width [min]	Area [mAU*s]	Height [mAU]	Area %
1	13.538	MF	0.8326	3410.42017	68.26711	12.0145
2	16.015	FM	1.1330	2.49754e4	367.40823	87.9855
Totals :				2.83858e4	435.67534	

HPLC (OD column, 1.0 mL/min 0.5 % i-PrOH in n-hexane 30 min, UV 210 nm) retention times of 13.54 (minor) and 16.02 min (majorr) 76 % ee with Rh_2(S-PTAD)$_4$.

Compound 14 -Racemic

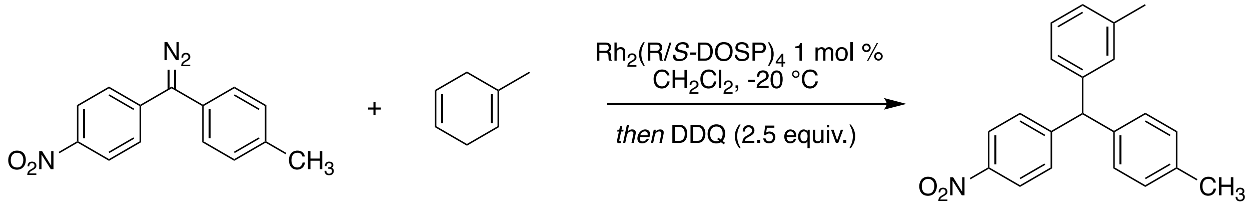

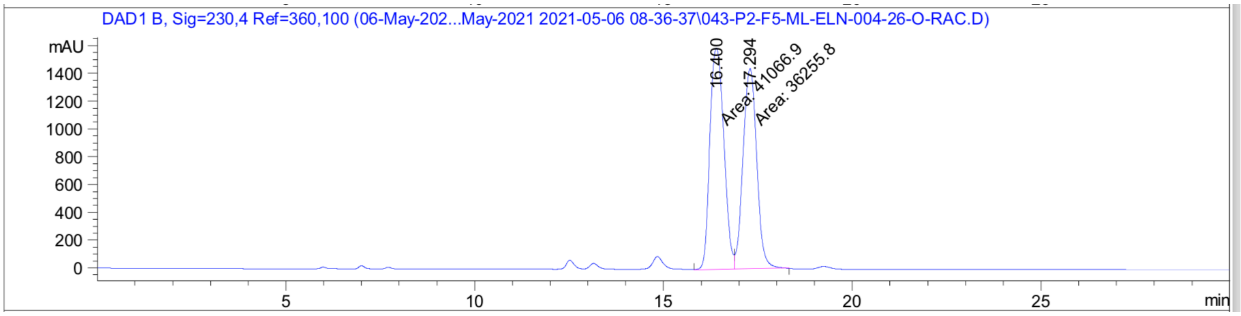

Signal 2: DAD1 B, Sig=230,4 Ref=360,100

Peak #	RetTime [min]	Type	Width [min]	Area [mAU*s]	Height [mAU]	Area %
1	16.400	MF	0.4299	4.10669e4	1592.10583	53.1110
2	17.294	FM	0.4195	3.62558e4	1440.44507	46.8890

Totals : 7.73227e4 3032.55090

Compound 14 -Chiral

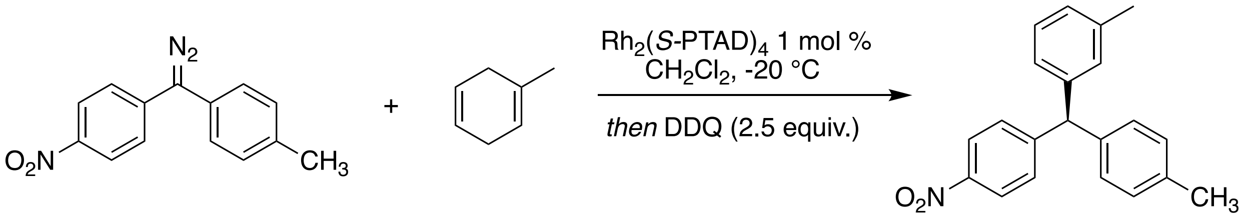

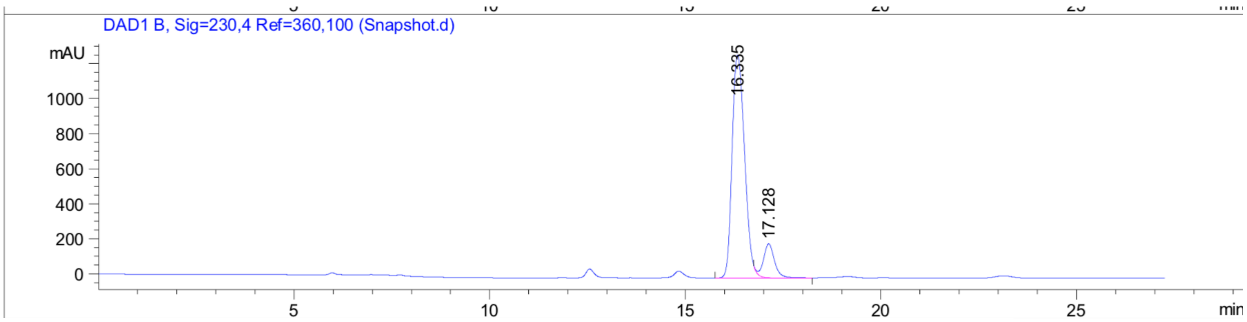

Signal 2: DAD1 B, Sig=230,4 Ref=360,100

Peak #	RetTime [min]	Type	Width [min]	Area [mAU*s]	Height [mAU]	Area %
1	16.335	BV R	0.3530	2.82586e4	1272.74890	88.2069
2	17.128	VB E	0.2953	3778.10498	193.92400	11.7931

Totals : 3.20367e4 1466.67290

Compound 15 –Racemic HPLC traces for SI Table 1.

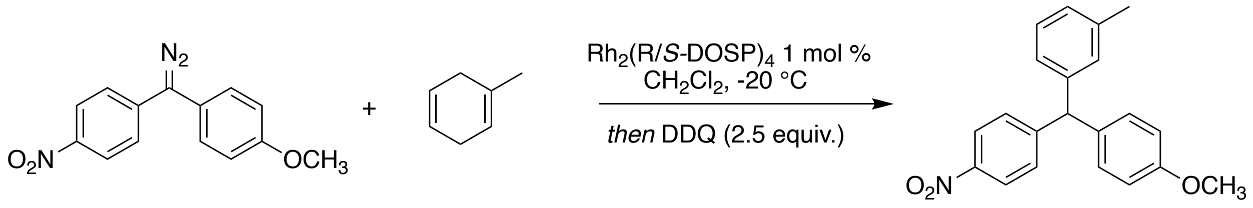

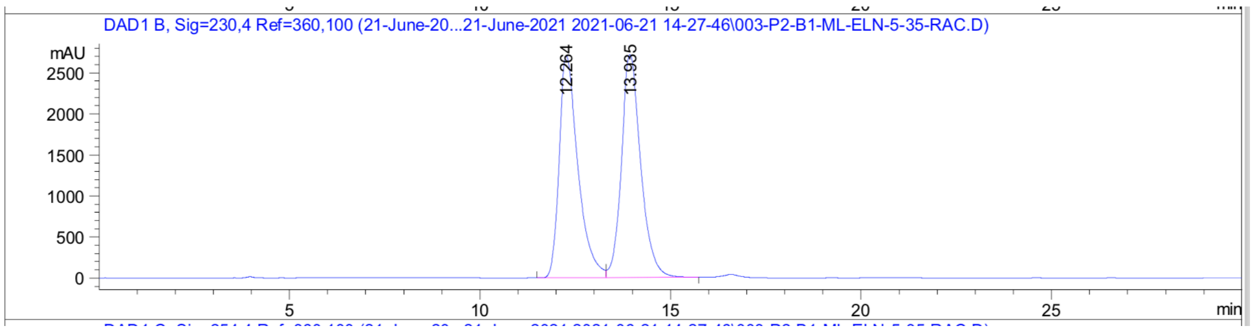

Signal 2: DAD1 B, Sig=230,4 Ref=360,100

Peak #	RetTime [min]	Type	Width [min]	Area [mAU*s]	Height [mAU]	Area %
1	12.264	BV	0.5077	9.25909e4	2702.52637	49.1916
2	13.935	VB	0.5229	9.56341e4	2715.59790	50.8084
Totals :				1.88225e5	5418.12427	

HPLC (ODH column, 0.8 mL/min 2.25 % i-PrOH in n-hexane 30 min, UV 230 nm) retention times of 12.26 and 13.94 min catalyzed by Rh_2(*R/*S-DOSP)$_4$.

Compound 15-Chiral

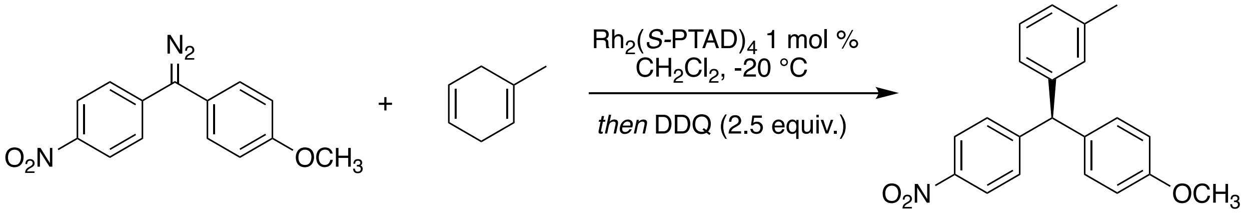

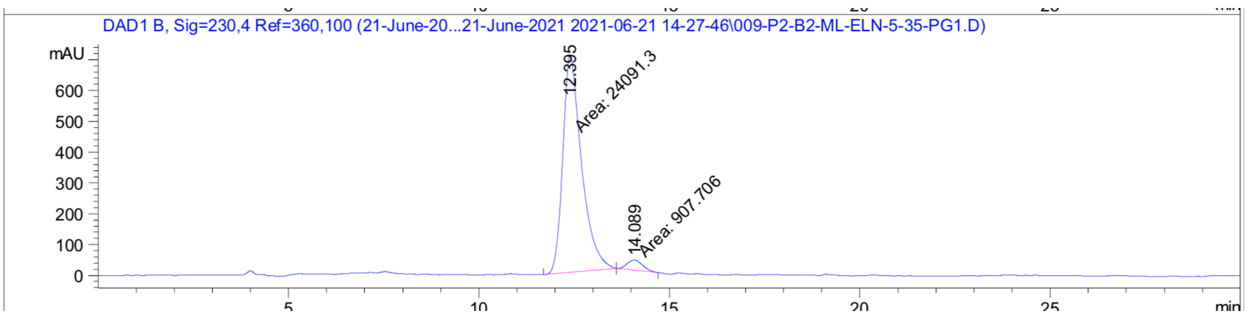

Signal 2: DAD1 B, Sig=230,4 Ref=360,100

Peak #	RetTime [min]	Type	Width [min]	Area [mAU*s]	Height [mAU]	Area %
1	12.395	MM T	0.6539	2.40913e4	705.28912	96.3690
2	14.089	MM T	0.4632	907.70624	32.66312	3.6310
Totals :				2.49990e4	737.95225	

HPLC (ODH column, 0.8 mL/min 2.25 % i-PrOH in n-hexane 30 min, UV 230 nm) retention times of 12.40 (major) and 14.09 min (minor) 93 % ee with Rh_2(S-PTAD)$_4$.

Compound 15-Chiral

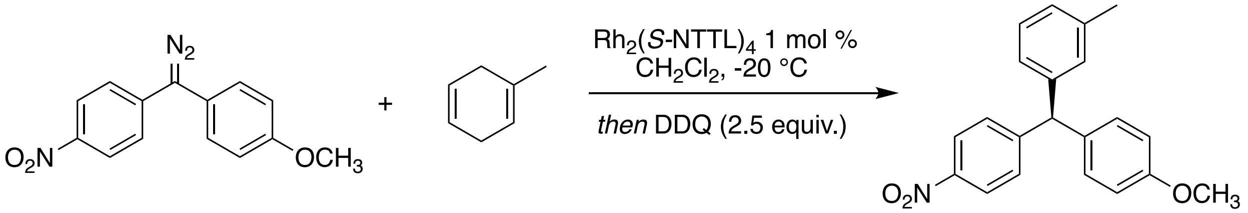

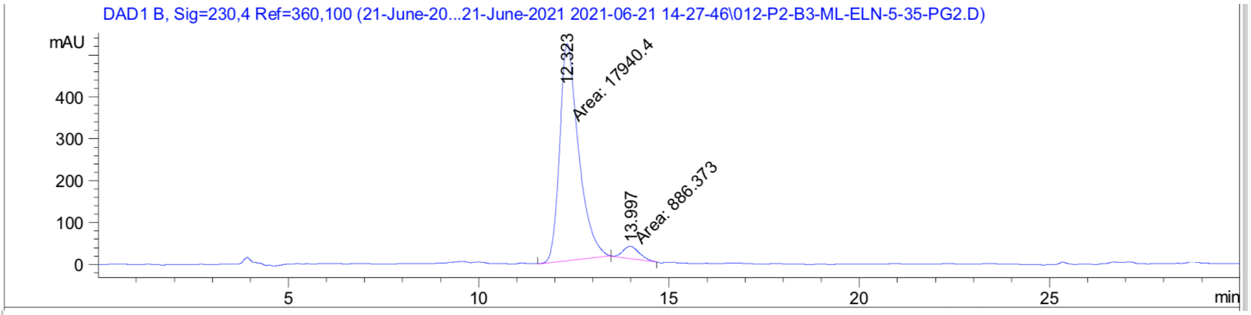

Signal 2: DAD1 B, Sig=230,4 Ref=360,100

Peak #	RetTime [min]	Type	Width [min]	Area [mAU*s]	Height [mAU]	Area %
1	12.323	MM T	0.6867	1.79404e4	518.19061	95.2920
2	13.997	MM T	0.5478	886.37262	29.70613	4.7080
Totals :				1.88268e4	547.89674	

HPLC (ODH column, 0.8 mL/min 2.25 % i-PrOH in n-hexane 30 min, UV 230 nm) retention times of 12.32(major) and 14.0 min (minor) 91 % ee with Rh_2(S-NTTL)$_4$.

Compound 15-Chiral

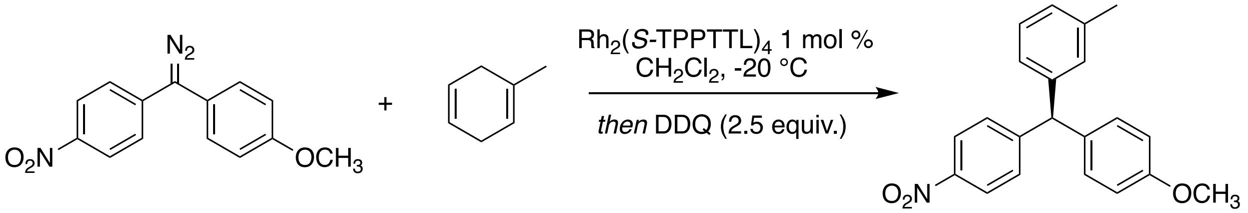

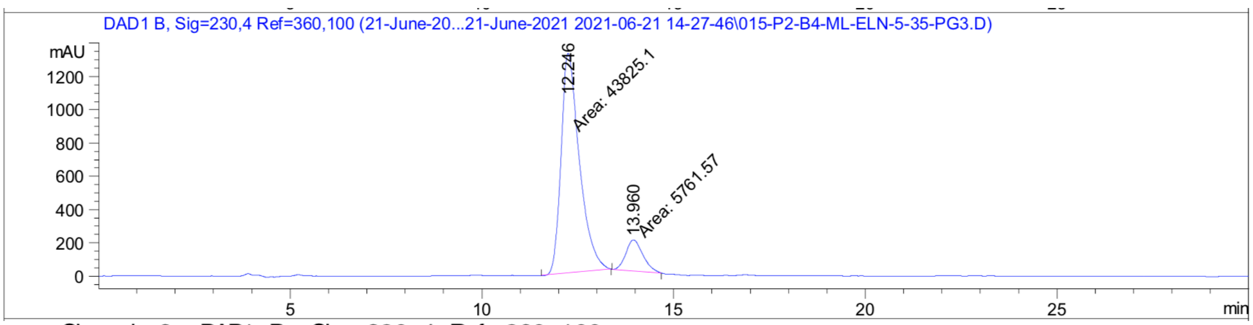

Signal 2: DAD1 B, Sig=230,4 Ref=360,100

Peak #	RetTime [min]	Type	Width [min]	Area [mAU*s]	Height [mAU]	Area %
1	12.246	MM T	0.6479	4.38251e4	1319.21460	88.3808
2	13.960	MM T	0.5150	5761.57227	186.45192	11.6192

Totals : 4.95866e4 1505.66652

HPLC (ODH column, 0.8 mL/min 2.25 % i-PrOH in n-hexane 30 min, UV 230 nm) retention times of 12.26 (major) and 13.96 min (minor) 77 % ee with Rh_2(S-TPPTTL)$_4$.

Compound 15-Chiral

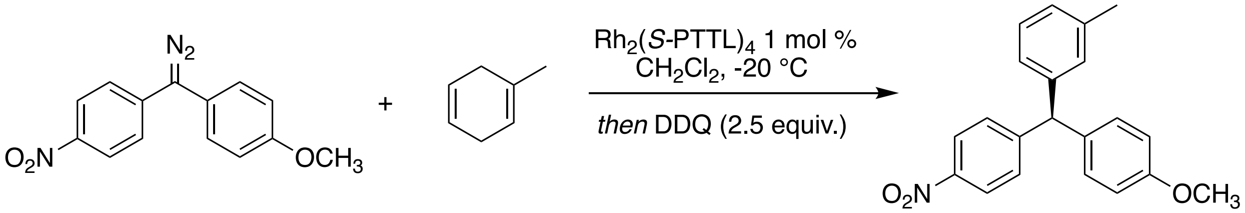

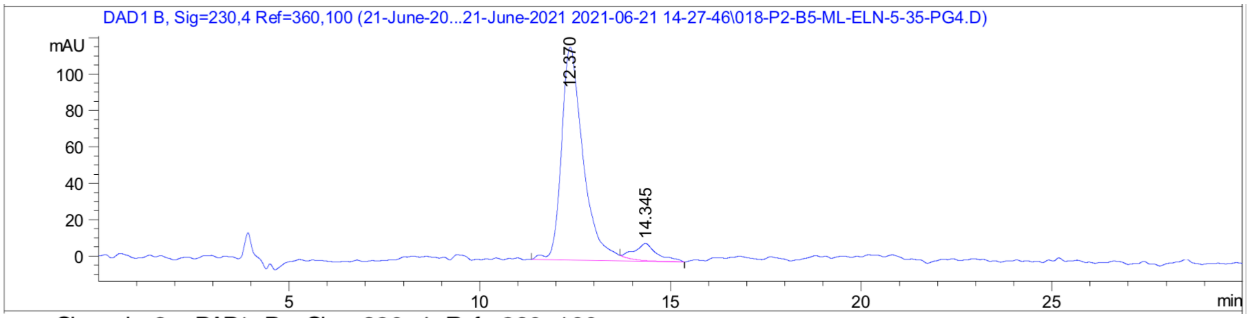

Signal 2: DAD1 B, Sig=230,4 Ref=360,100

Peak #	RetTime [min]	Type	Width [min]	Area [mAU*s]	Height [mAU]	Area %
1	12.370	VV R	0.5787	4645.92920	116.84219	92.2497
2	14.345	VB E	0.5517	390.32297	9.45325	7.7503

Totals : 5036.25217 126.29545

HPLC (ODH column, 0.8 mL/min 2.25 % i-PrOH in n-hexane 30 min, UV 230 nm) retention times of 12.26 (major) and 13.96 min (minor) 84 % ee with Rh_2(S-PTTL)$_4$.

Compound 17 -Racemic Table 2. Catalyst Screen

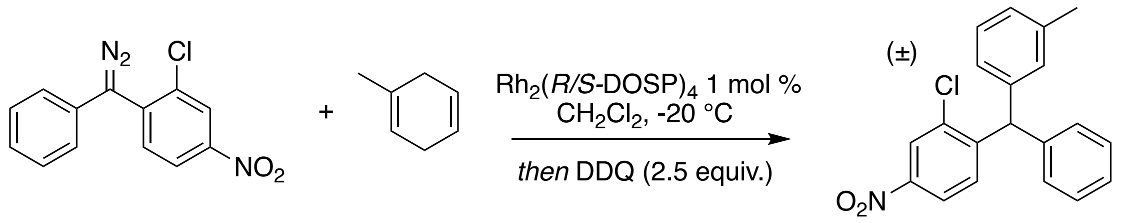

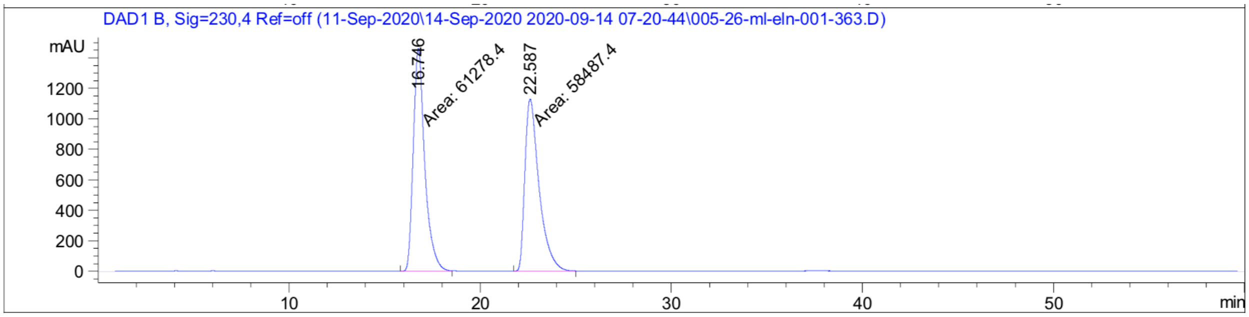

Signal 2: DAD1 B, Sig=230,4 Ref=off

Peak #	RetTime [min]	Type	Width [min]	Area [mAU*s]	Height [mAU]	Area %
1	16.746	MM	0.6988	6.12784e4	1461.53943	51.1652
2	22.587	MM	0.8622	5.84874e4	1130.60669	48.8348
Totals :				1.19766e5	2592.14612	

Compound 17 –Chiral Table 2. Catalyst Screen

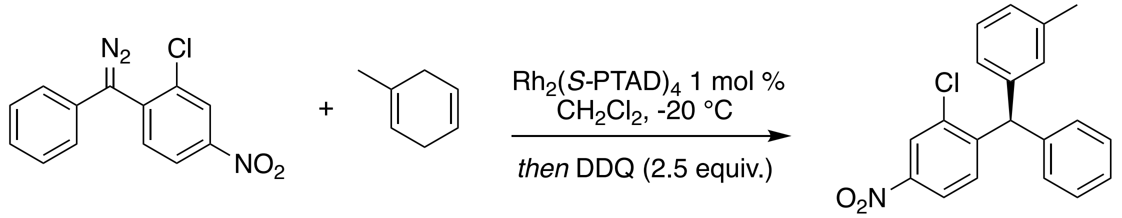

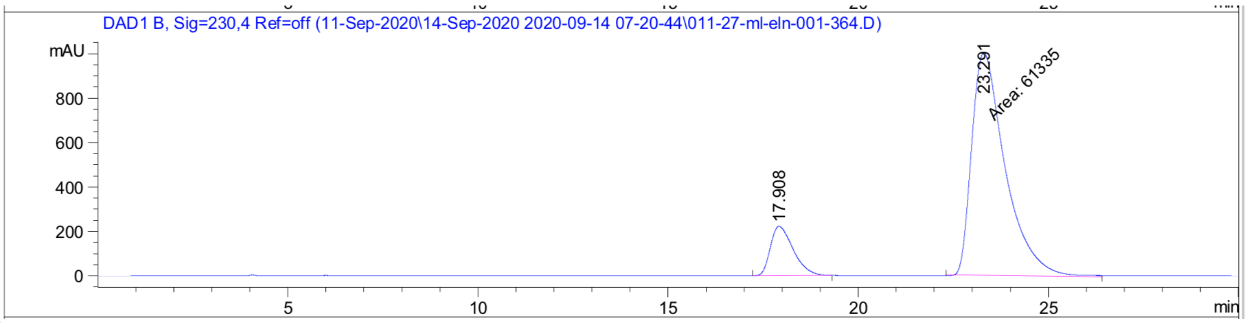

Signal 2: DAD1 B, Sig=230,4 Ref=off

Peak #	RetTime [min]	Type	Width [min]	Area [mAU*s]	Height [mAU]	Area %
1	17.908	VV R	0.4904	9232.09668	222.14276	13.0827
2	23.291	MM	1.0208	6.13350e4	1001.42139	86.9173

Totals : 7.05671e4 1223.56415

Compound 17 –Chiral Table 2. Catalyst Screen

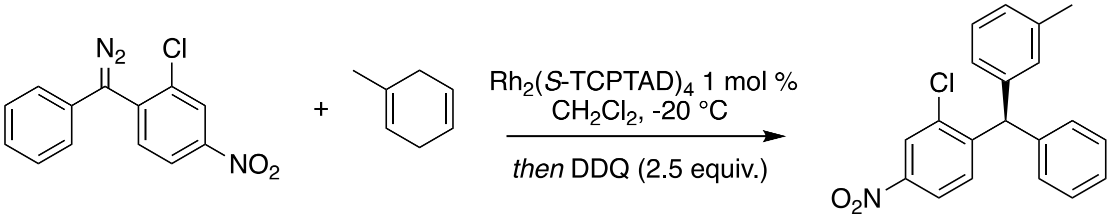

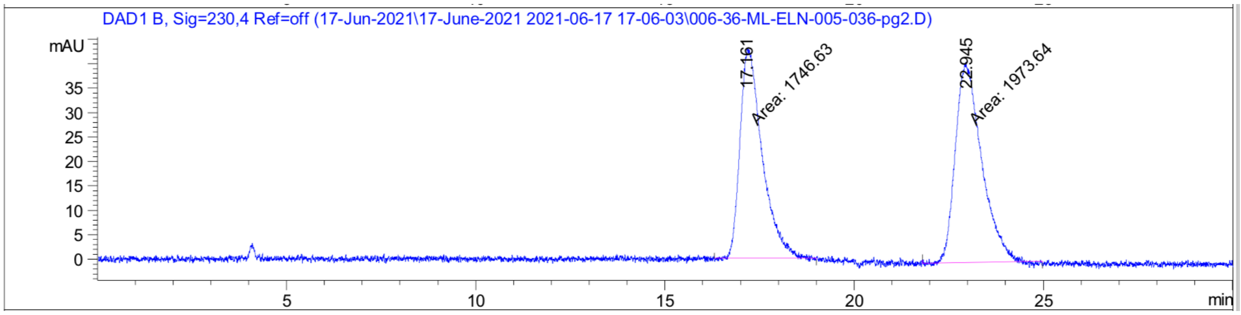

Signal 2: DAD1 B, Sig=230,4 Ref=off

Peak #	RetTime [min]	Type	Width [min]	Area [mAU*s]	Height [mAU]	Area %
1	17.161	MM	0.6777	1746.63354	42.95704	46.9490
2	22.945	MM	0.8026	1973.64307	40.98562	53.0510
Totals :				3720.27661	83.94266	

Compound 17 –Chiral Table 2. Catalyst Screen

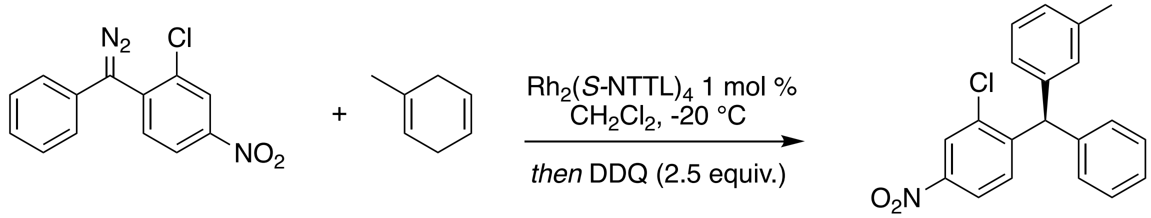

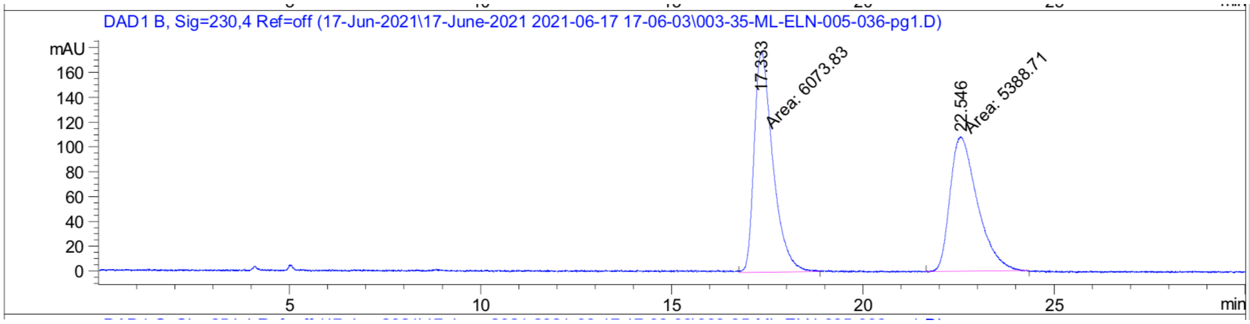

Signal 2: DAD1 B, Sig=230,4 Ref=off

Peak #	RetTime [min]	Type	Width [min]	Area [mAU*s]	Height [mAU]	Area %
1	17.333	MM	0.5676	6073.83252	178.35365	52.9885
2	22.546	MM	0.8256	5388.71484	108.78693	47.0115

Totals : 1.14625e4 287.14058

Compound 17 –Chiral Table 2. Catalyst Screen

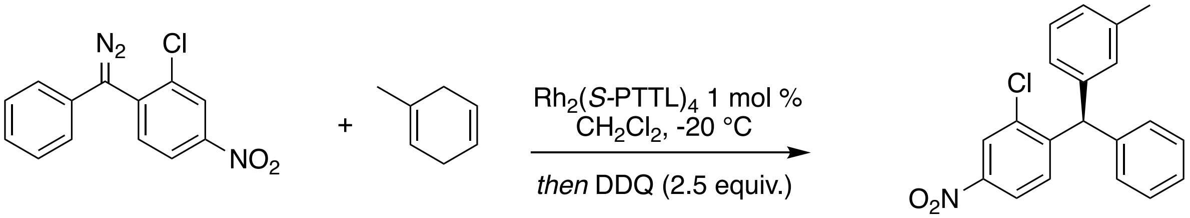

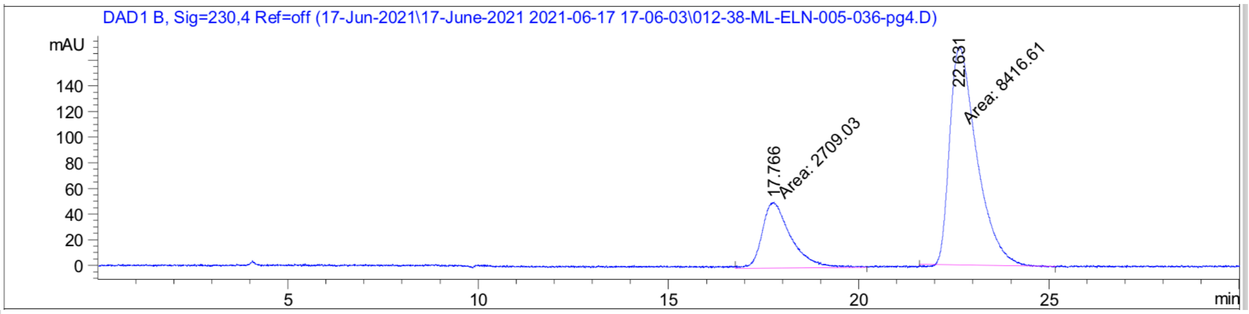

Signal 2: DAD1 B, Sig=230,4 Ref=off

Peak #	RetTime [min]	Type	Width [min]	Area [mAU*s]	Height [mAU]	Area %
1	17.766	MM	0.8732	2709.02710	51.70412	24.3494
2	22.631	MM	0.8247	8416.60938	170.09402	75.6506

Totals : 1.11256e4 221.79815

Compound 17 –Chiral Table 2. Catalyst Screen

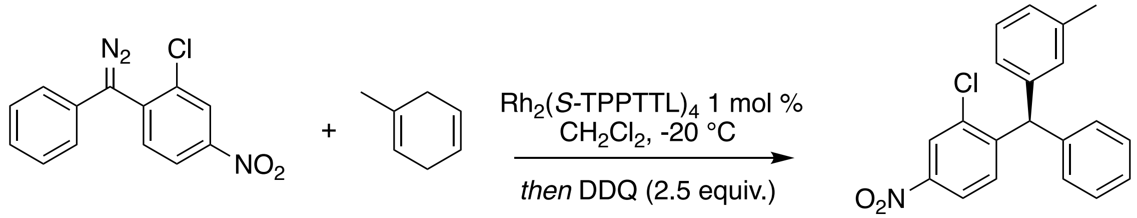

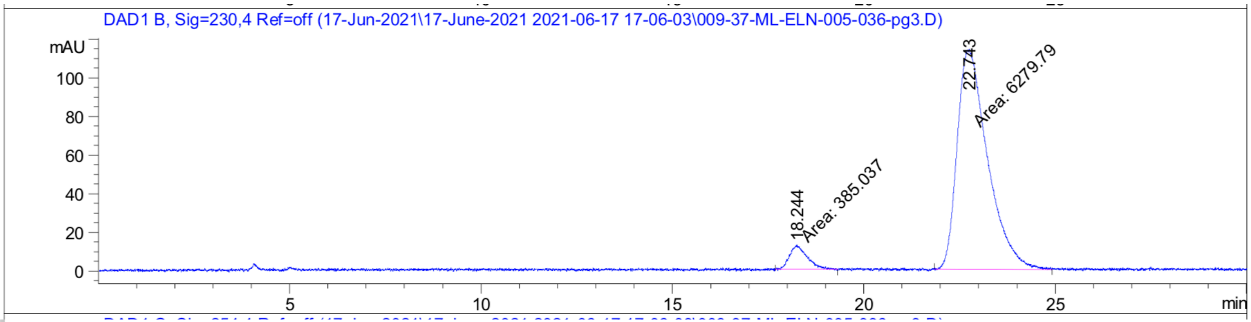

Signal 2: DAD1 B, Sig=230,4 Ref=off

Peak #	RetTime [min]	Type	Width [min]	Area [mAU*s]	Height [mAU]	Area %
1	18.244	MM	0.5177	385.03741	12.39615	5.7772
2	22.743	MM	0.9188	6279.78906	113.91827	94.2228
Totals :				6664.82648	126.31442	

HPLC (ODH column, 0.8 mL/min 0.3 % i-PrOH in n-hexane 30 min, UV 230 nm) retention times of 18.24(minor) and 22.74 min (major) 88 % ee with Rh_2(S-TPPTTL)$_4$.

Compound 18 –Racemic

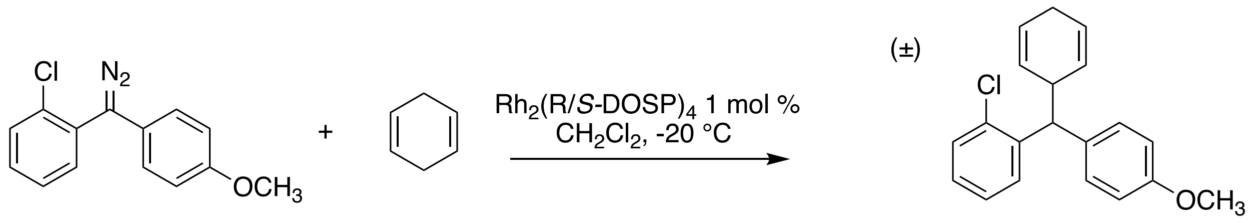

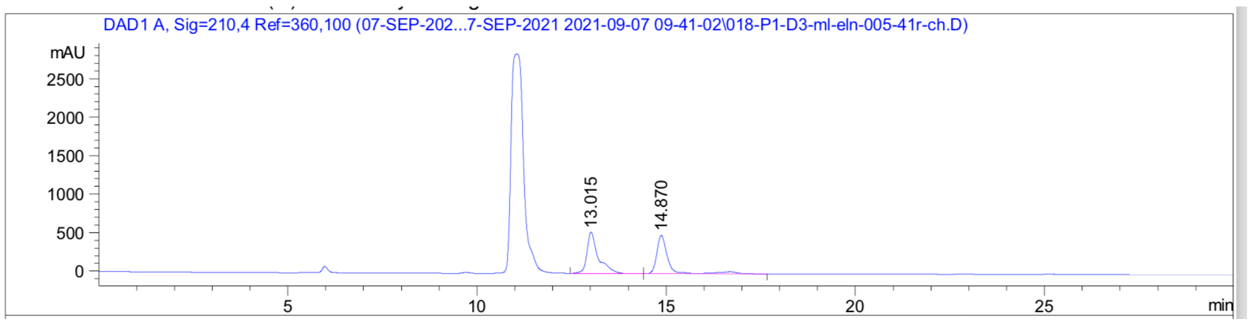

Signal 1: DAD1 A, Sig=210,4 Ref=360,100

Peak #	RetTime [min]	Type	Width [min]	Area [mAU*s]	Height [mAU]	Area %
1	13.015	VB	0.3017	1.13815e4	540.45325	51.9915
2	14.870	BV R	0.2821	1.05096e4	500.89468	48.0085

Totals :				2.18910e4	1041.34793	

Compound 18 –Chiral

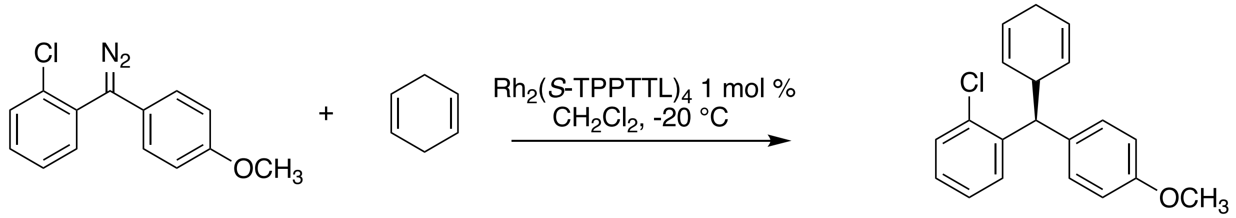

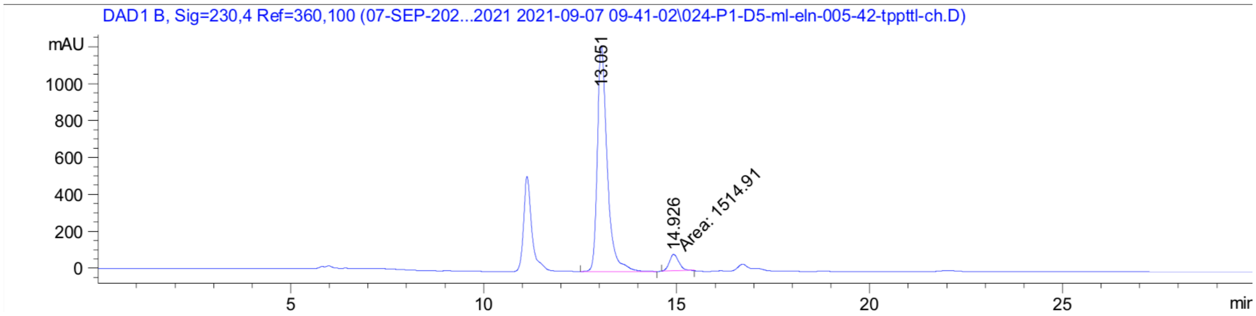

Signal 2: DAD1 B, Sig=230,4 Ref=360,100

Peak #	RetTime [min]	Type	Width [min]	Area [mAU*s]	Height [mAU]	Area %
1	13.051	BB	0.2660	2.18171e4	1223.70215	93.5071
2	14.926	MM	0.2836	1514.91260	89.03326	6.4929
Totals :				2.33320e4	1312.73540	

s-tppttl HPLC (ADH column, 0.5 mL/min 0.5 % i-PrOH in n-hexane 30 min, UV 230 nm) retention times of 13.86 (major) and 14.90 min (minor) 87 % ee with $Rh_2(S\text{-}TPPTTL)_4$.

Compound 18 –Chiral

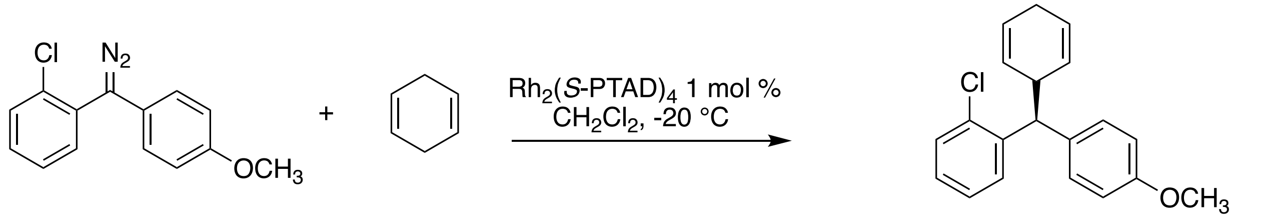

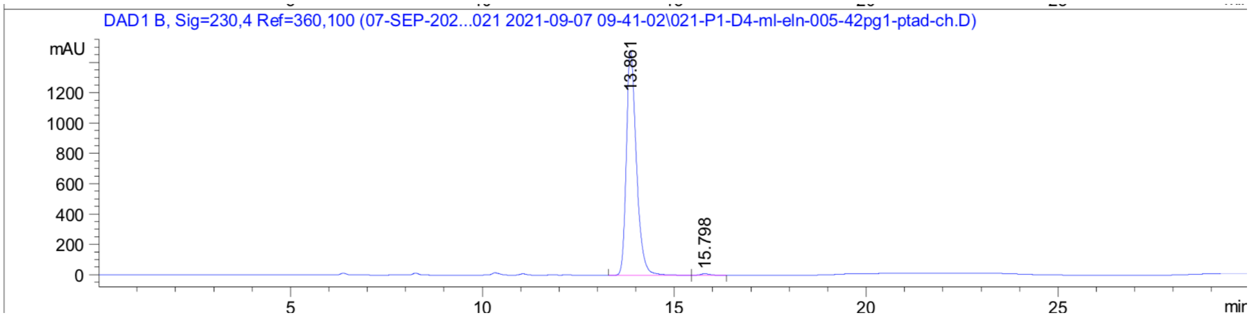

Signal 2: DAD1 B, Sig=230,4 Ref=360,100

Peak #	RetTime [min]	Type	Width [min]	Area [mAU*s]	Height [mAU]	Area %
1	13.861	BB	0.2767	2.70252e4	1482.66650	99.3728
2	15.798	BB	0.2710	170.55885	9.70782	0.6272

Totals : 2.71957e4 1492.37432

s-ptad HPLC (ADH column, 0.5 mL/min 0.5 % i-PrOH in n-hexane 30 min, UV 230 nm) retention times of 13.86 (major) and 15.80 min (minor) 98 % ee with Rh_2(S-PTAD)$_4$.

Compound 19 as C-H Insertion Intermediate –Racemic

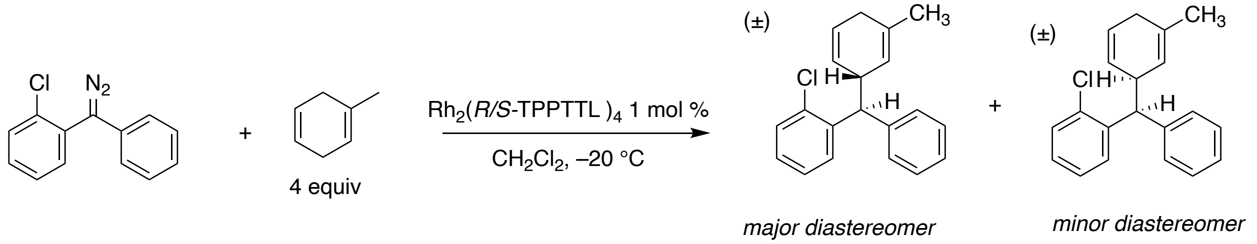

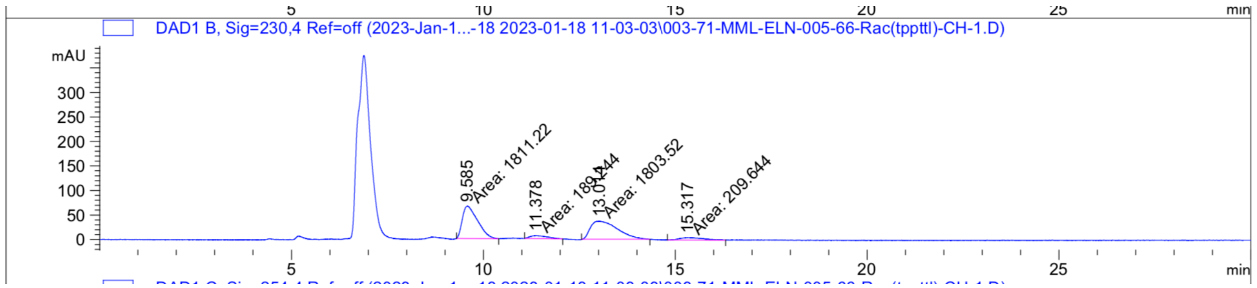

Signal 2: DAD1 B, Sig=230,4 Ref=off

Peak #	RetTime [min]	Type	Width [min]	Area [mAU*s]	Height [mAU]	Area %
1	9.585	MM	0.4569	1811.21509	66.06657	45.1267
2	11.378	MM	0.5046	189.24393	6.25117	4.7150
3	13.014	MM	0.8125	1803.51831	36.99650	44.9349
4	15.317	MM	0.6769	209.64355	5.16168	5.2233

Totals : 4013.62088 114.47591

ODH_30min_0.8ML_0 .1%.M (Sequence Method)

This racemic trace shows us the major diastereomer corresponds to peaks 1 and 3. The minor diastereomer corresponds to peaks 2 and 4.

Compound 19 as C-H Insertion Intermediate –Chiral

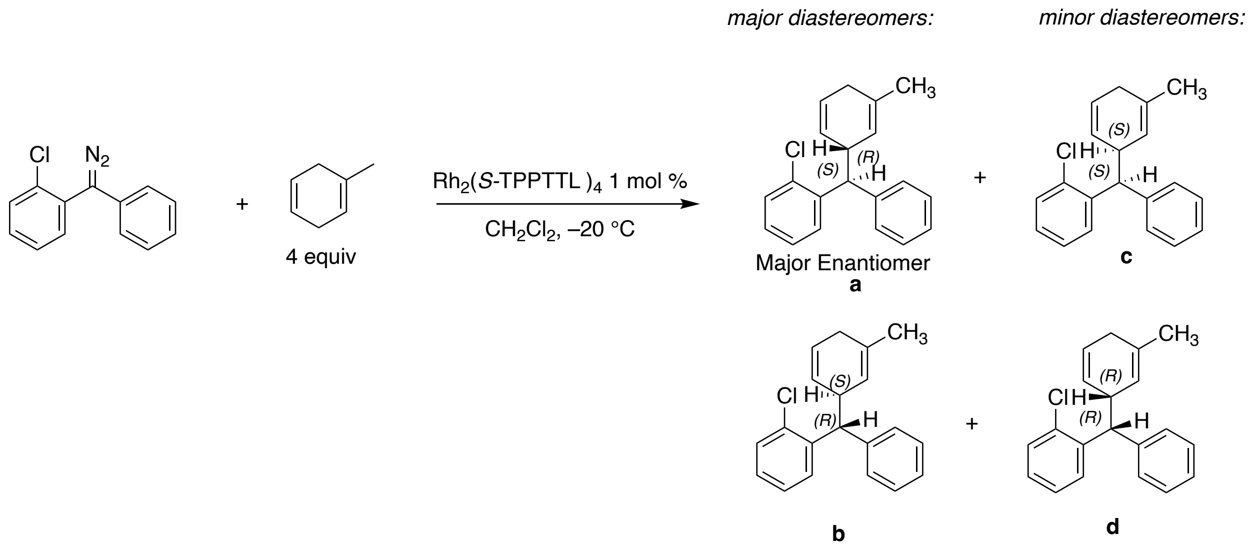

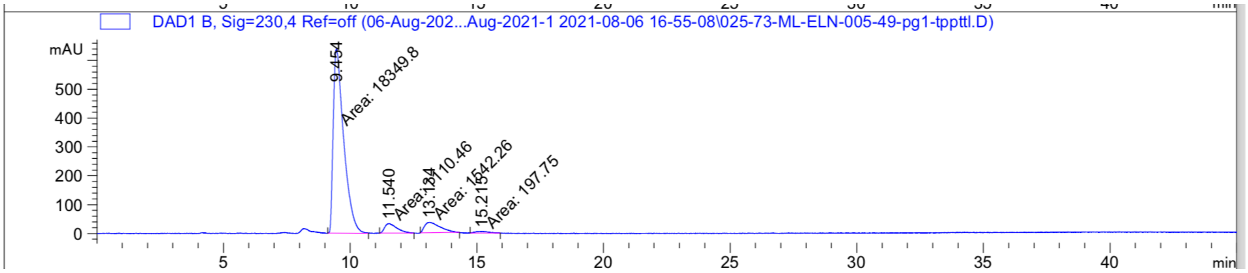


```
Signal 2: DAD1 B, Sig=230,4 Ref=off
```

Peak #	RetTime [min]	Type	Width [min]	Area [mAU*s]	Height [mAU]	Area %
1	9.454	MM	0.4790	1.83498e4	638.52008	86.5546
2	11.540	MM	0.5602	1110.45618	33.03613	5.2379
3	13.134	MM	0.7036	1542.26001	36.53149	7.2747
4	15.215	MM	0.5374	197.74982	6.13309	0.9328
Totals :				2.12003e4	714.22078	

HPLC (ODH column, 0.8 mL/min .1 % i-PrOH in n-hexane 45 min, UV 230 nm) retention times of 9.5 (major) and 13.1 min (minor) 85 % ee with Rh_2(S-TPPTTL)$_4$ for the major diastereomer; 11 .6 (major) and 15.2 min (minor) 70 % ee with Rh_2(S-TPPTTL)$_4$ for the minor diastereomers.

The major enantiomer was assigned to the major peak based on the assumption that this compound will follow the pattern shown in Scheme 5. The major enantiomer is assigned based on the crystal structure obtained from compound **11a.** The crude C-H insertion (both diastereomers) were oxidized to the final triarylmethane compound **19**. To account for the minor diastereomer, we obtain a calculated ee of 83% based on the 90:10 ratio and an estimated ee value of 83% was assigned.

Compound 20 –Racemic

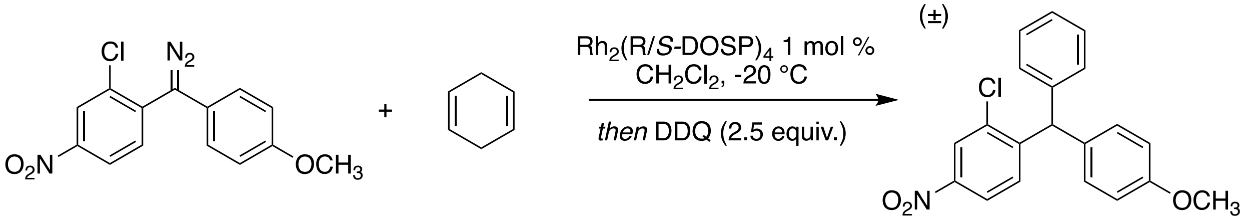

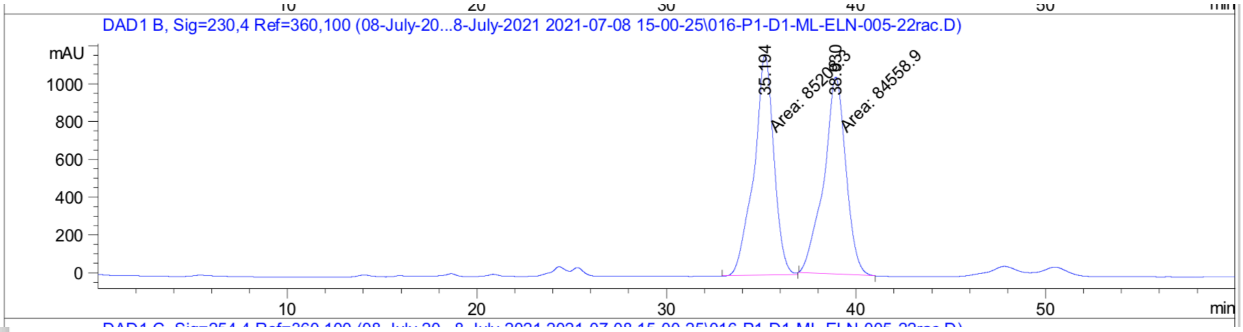

Signal 2: DAD1 B, Sig=230,4 Ref=360,100

Peak #	RetTime [min]	Type	Width [min]	Area [mAU*s]	Height [mAU]	Area %
1	35.194	MM	1.2200	8.52083e4	1164.01221	50.1912
2	38.930	MM	1.3548	8.45589e4	1040.22314	49.8088

Totals : 1.69767e5 2204.23535

Compound 20 –Chiral

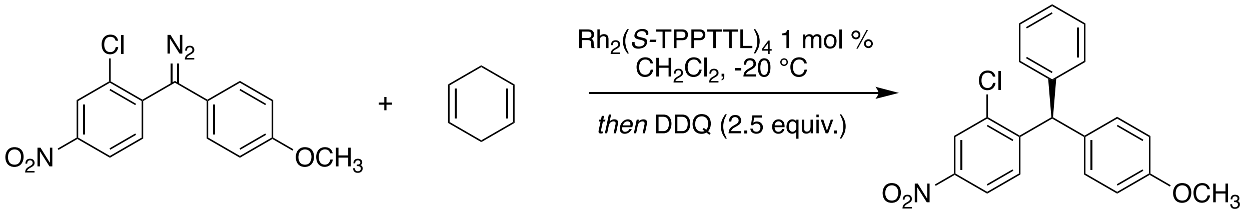

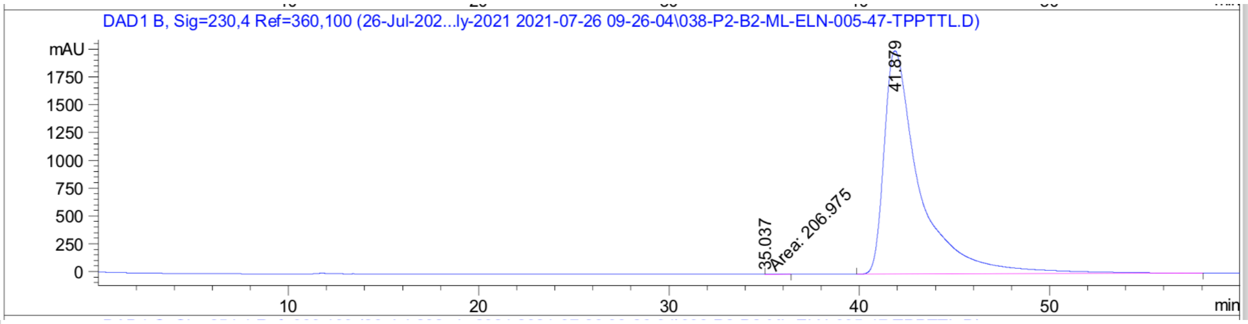

Signal 2: DAD1 B, Sig=230,4 Ref=360,100

Peak #	RetTime [min]	Type	Width [min]	Area [mAU*s]	Height [mAU]	Area %
1	35.037	MM	0.4681	206.97479	5.24418	0.0786
2	41.879	BB	1.8818	2.63210e5	2009.94714	99.9214
Totals :				2.63417e5	2015.19132	

HPLC (ODH column, 0.25 mL/min 2.0 % i-PrOH in n-hexane 60 min, UV 230 nm) retention times of 35.04 (minor) and 41.88 min (major) 98 % ee with Rh_2(S-TPPTTL)$_4$.

Compound 21 as C-H Insertion Intermediate –Racemic

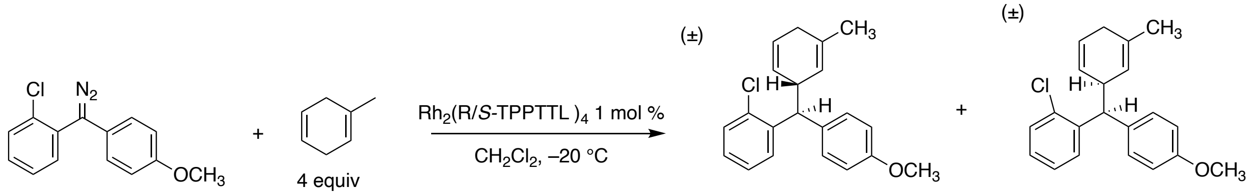

1: 1 ratio

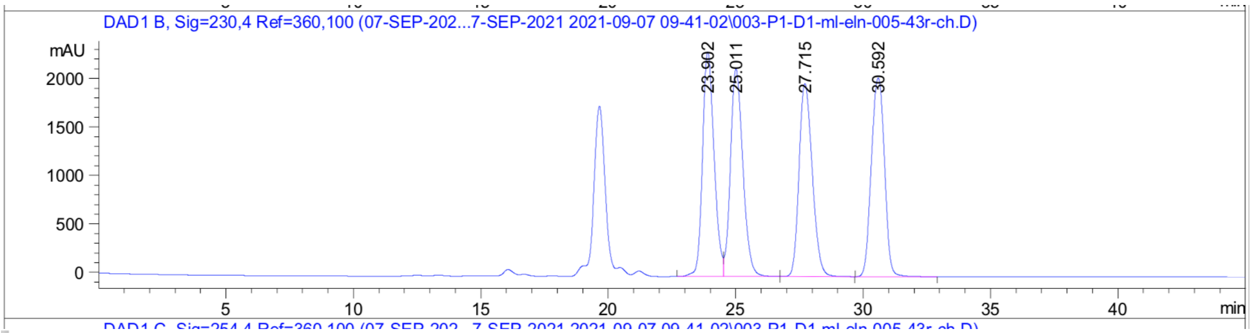

Signal 2: DAD1 B, Sig=230,4 Ref=360,100

Peak #	RetTime [min]	Type	Width [min]	Area [mAU*s]	Height [mAU]	Area %
1	23.902	BV	0.4861	7.31142e4	2317.15845	25.0995
2	25.011	VB	0.5242	7.28261e4	2144.24634	25.0005
3	27.715	BB	0.5606	7.18309e4	1992.59241	24.6589
4	30.592	BB	0.5824	7.35267e4	2050.28882	25.2411
Totals :				2.91298e5	8504.28601	

In racemic reaction d.r. is 1:1; making it unclear which peak corresponds to which enantiomer.

Compound 21 as C-H Insertion Intermediate –Chiral

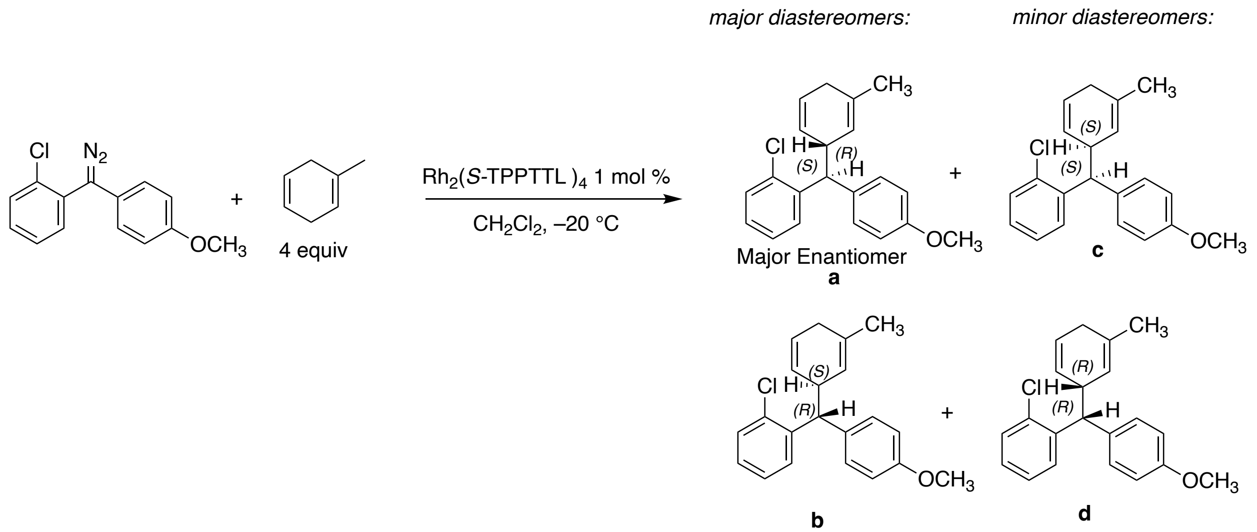

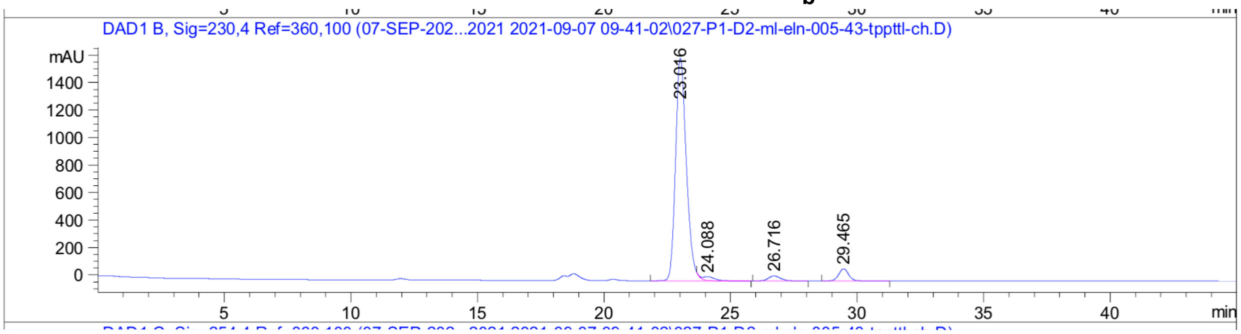

Signal 2: DAD1 B, Sig=230,4 Ref=360,100

Peak #	RetTime [min]	Type	Width [min]	Area [mAU*s]	Height [mAU]	Area %
1	23.016	BV R	0.4585	4.83723e4	1619.46423	91.5082
2	24.088	VB E	0.4772	895.12067	27.39291	1.6933
3	26.716	BB	0.5045	1197.92212	36.53453	2.2662
4	29.465	BB	0.4188	2395.80298	88.21896	4.5323
Totals :				5.28611e4	1771.61064	

HPLC (ADH column, 0.25 mL/min **0.5 % i-PrOH in n-hexane** 45 min, UV 230 nm) retention times of 23.0 min (major) and 24.1 min, 26.7 min and 29.5 min as minor peaks with Rh_2(S-TPPTTL)$_4$. The ratio of peaks is 91.5:1.7:2.3: 4.5.

The major enantiomer was assigned to the major peak based on the assumption that this compound will follow the pattern shown in Scheme 5. The major enantiomer is assigned based on the crystal structure obtained from compound **11a,** however which peak corresponds to the minor enantiomer is unclear.

Ee was calculated to be >82% based on the assumption that the diasteriomeric peak could be any of the minor peaks, and the ee is then assumed subtracting the sum of the 3 minor peaks (91.5-8.5).

Compound 22 –Racemic

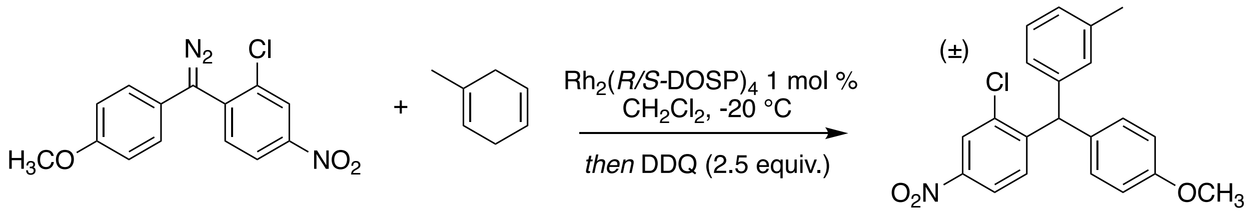

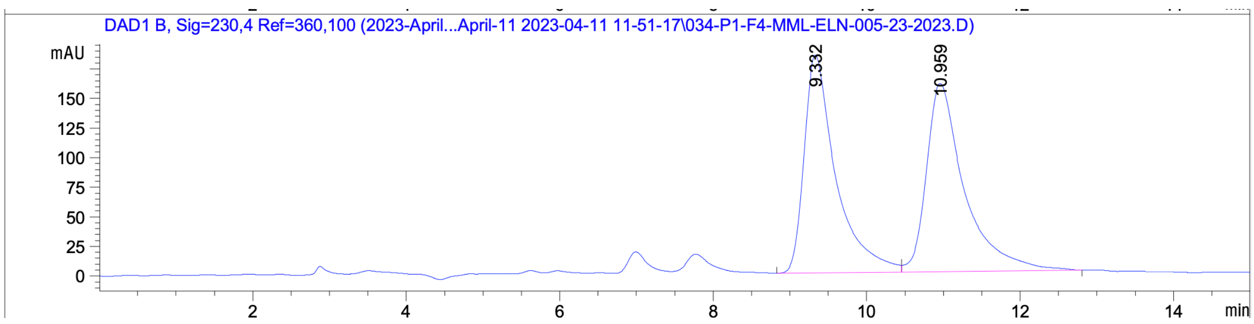

Signal 2: DAD1 B, Sig=230,4 Ref=360,100

Peak #	RetTime [min]	Type	Width [min]	Area [mAU*s]	Height [mAU]	Area %
1	9.332	BV	0.4152	5286.01904	183.98650	49.1916
2	10.959	VB	0.4965	5459.74756	159.04063	50.8084
Totals :				1.07458e4	343.02713	

Compound 22 –Chiral

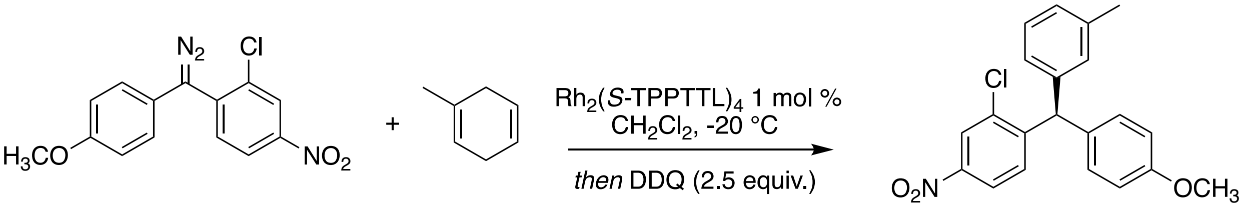

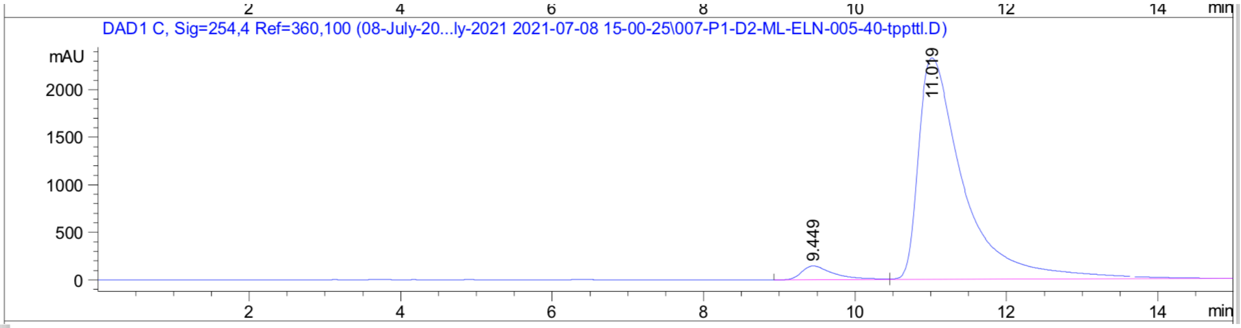

Signal 3: DAD1 C, Sig=254,4 Ref=360,100

Peak #	RetTime [min]	Type	Width [min]	Area [mAU*s]	Height [mAU]	Area %
1	9.449	BV	0.4316	4328.86230	146.15254	4.2674
2	11.019	VBA	0.6084	9.71105e4	2329.03613	95.7326
Totals :				1.01439e5	2475.18867	

HPLC (ODH column, 1.0mL/min 1.0 % i-PrOH in n-hexane 15 min, UV 230 nm) retention times of 9.45 (minor) and 11.02 min (major) 91 % ee with Rh_2(S-TPPTTL)$_4$

Compound 23 –Racemic

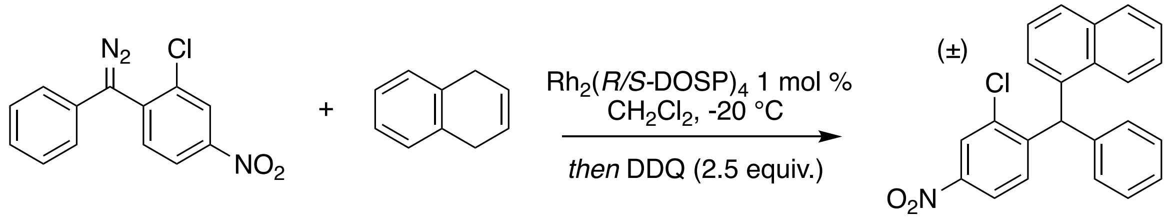

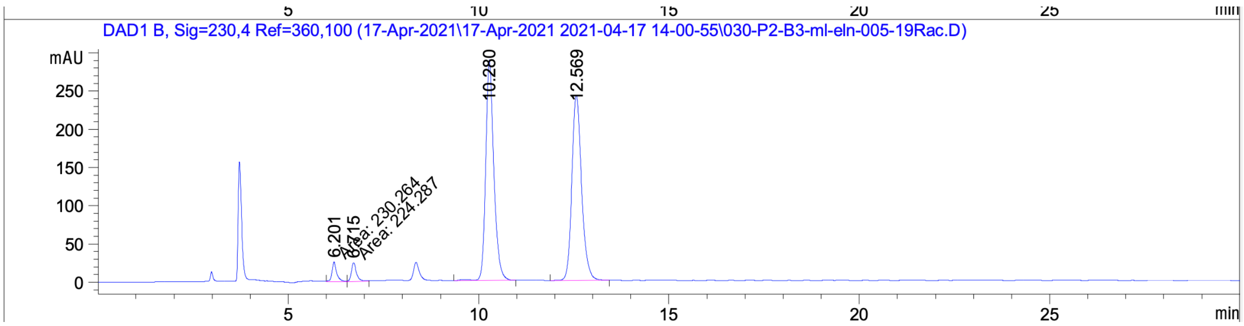

Signal 2: DAD1 B, Sig=230,4 Ref=360,100

Peak #	RetTime [min]	Type	Width [min]	Area [mAU*s]	Height [mAU]	Area %
1	6.201	MF	0.1490	230.26440	25.76005	2.5482
2	6.715	FM	0.1532	224.28708	24.39639	2.4821
3	10.280	VB R	0.2223	4286.21191	288.43939	47.4331
4	12.569	BB	0.2707	4295.56934	242.47028	47.5366

Compound 23 –Chiral

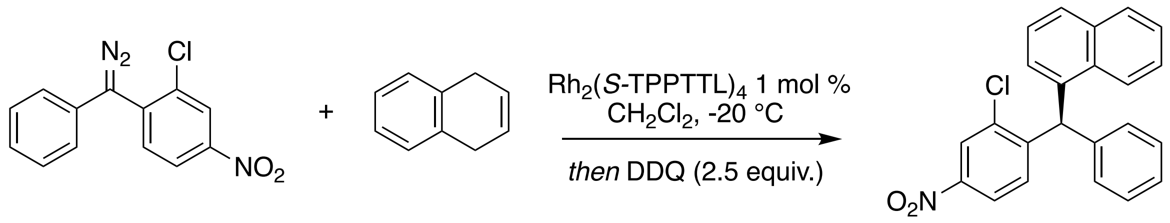

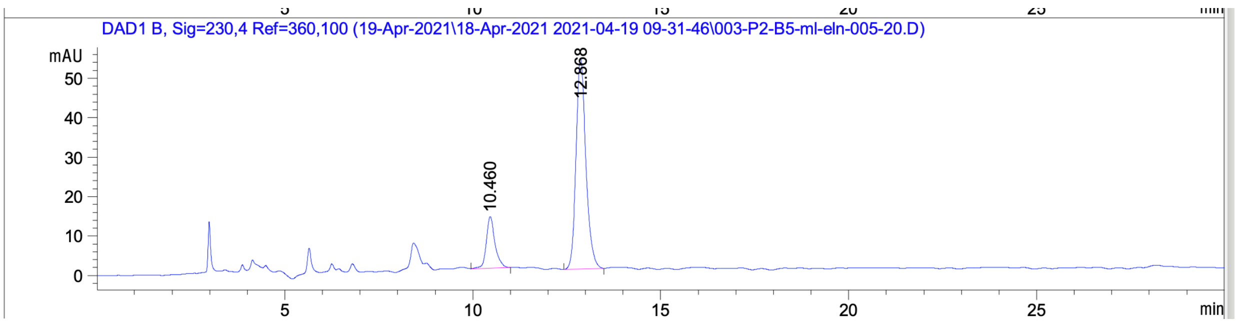

Signal 2: DAD1 B, Sig=230,4 Ref=360,100

Peak #	RetTime [min]	Type	Width [min]	Area [mAU*s]	Height [mAU]	Area %
1	10.460	BB	0.2359	208.43634	13.08079	17.6692
2	12.868	BB	0.2758	971.22510	53.50193	82.3308
Totals :				1179.66144	66.58272	

Compound 24 –Racemic

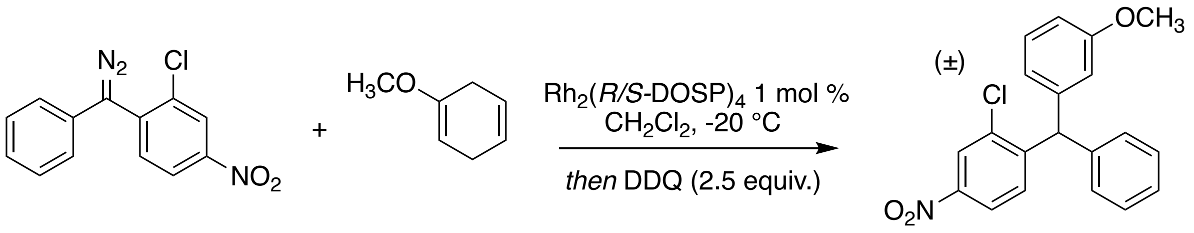

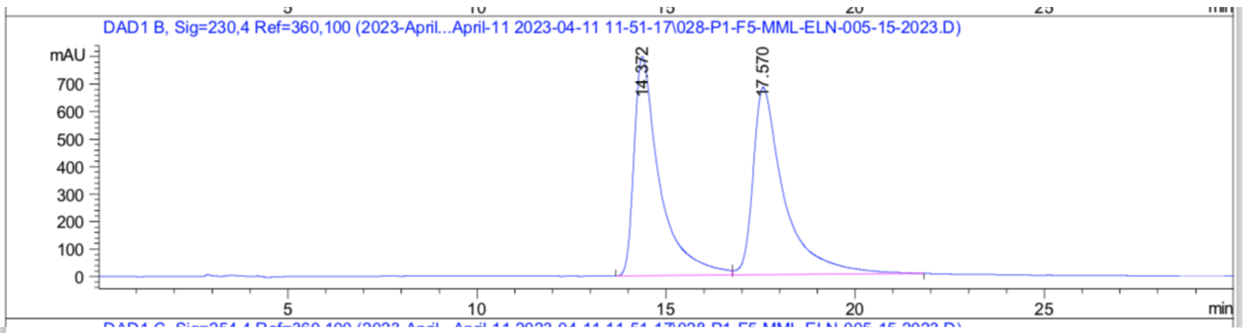

Signal 2: DAD1 B, Sig=230,4 Ref=360,100

Peak #	RetTime [min]	Type	Width [min]	Area [mAU*s]	Height [mAU]	Area %
1	14.372	BV	0.6814	3.77297e4	798.30634	49.0370
2	17.570	VB	0.8222	3.92116e4	682.61328	50.9630
Totals :				7.69413e4	1480.91962	

HPLC (ODH column, 1.0 mL/min 1% i-PrOH in n-hexane 30 min, UV 230 nm) retention times of 14.3 and 17.6 min with Rh_2(*R/S*-DOSP)$_4$.

Compound 24 –Chiral

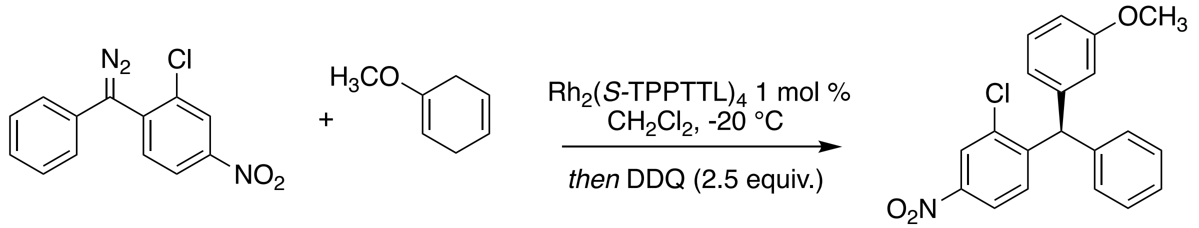

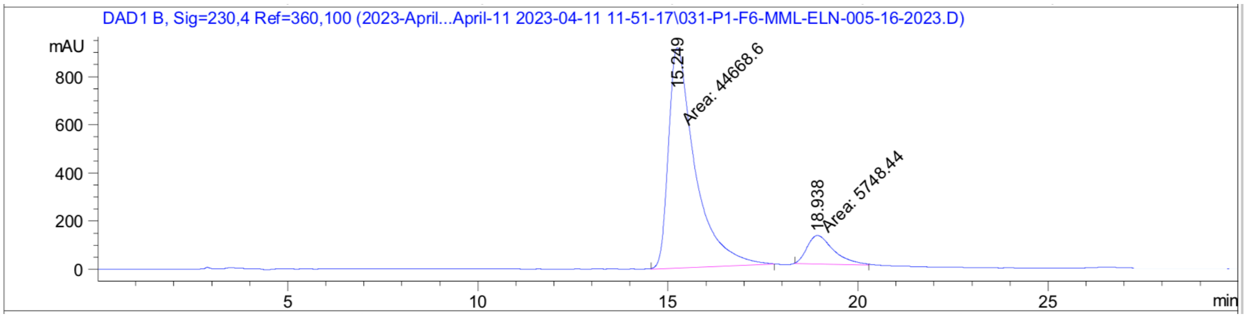

Signal 2: DAD1 B, Sig=230,4 Ref=360,100

Peak #	RetTime [min]	Type	Width [min]	Area [mAU*s]	Height [mAU]	Area %
1	15.249	MM	0.8137	4.46686e4	914.94696	88.5982
2	18.938	MM	0.8110	5748.44043	118.13127	11.4018
Totals :				5.04170e4	1033.07823	

HPLC (ODH column, 1.0 mL/min 1% i-PrOH in n-hexane 30 min, UV 230 nm) retention times of 15.2 (major) and 19.0 min (minor) 75 % ee with Rh_2(*S*-TPPTTL)$_4$.

Compound 25 –Racemic

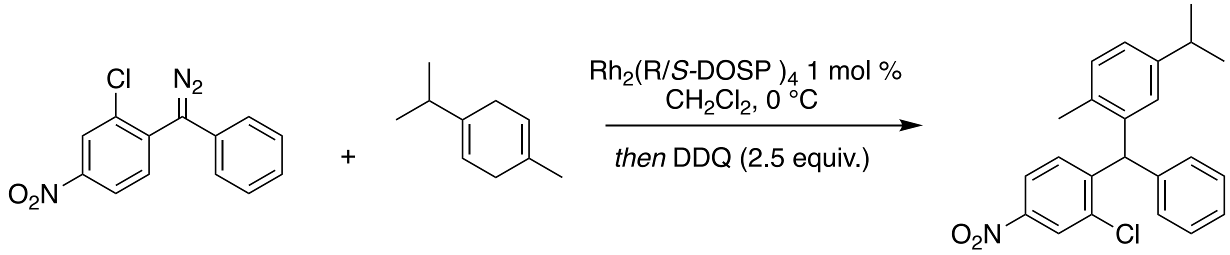

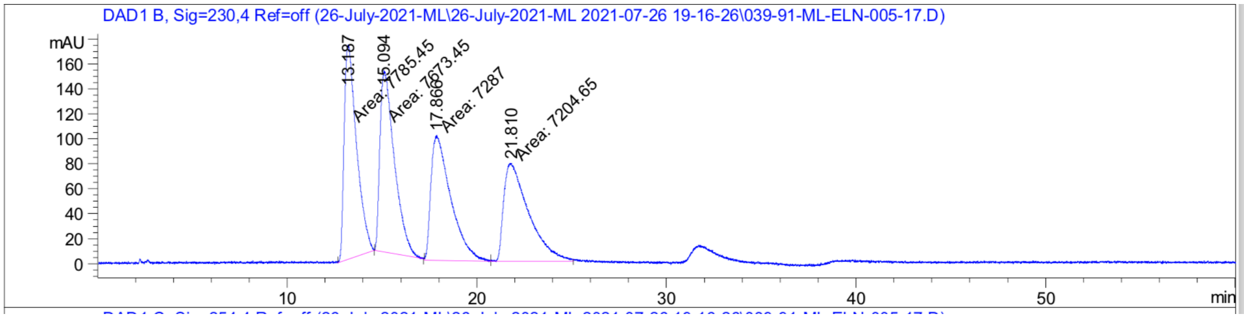

Signal 2: DAD1 B, Sig=230,4 Ref=off

Peak #	RetTime [min]	Type	Width [min]	Area [mAU*s]	Height [mAU]	Area %
1	13.187	MM T	0.7546	7785.45361	171.95609	25.9944
2	15.094	MM T	0.7704	7673.44727	145.45210	25.6204
3	17.866	MM	1.2102	7287.00342	100.35814	24.3301
4	21.810	MM	1.5218	7204.64697	78.90544	24.0551

Totals : 2.99506e4 496.67177

ODH_ 60min_1.5ML_0%.M

HPLC (ODH column, 1.5 mL/min 0% i-PrOH in n-hexane 60 min, UV 230 nm) retention times of and min with Rh_2(*R/S*-DOSP)$_4$.

Compound 25 –Chiral

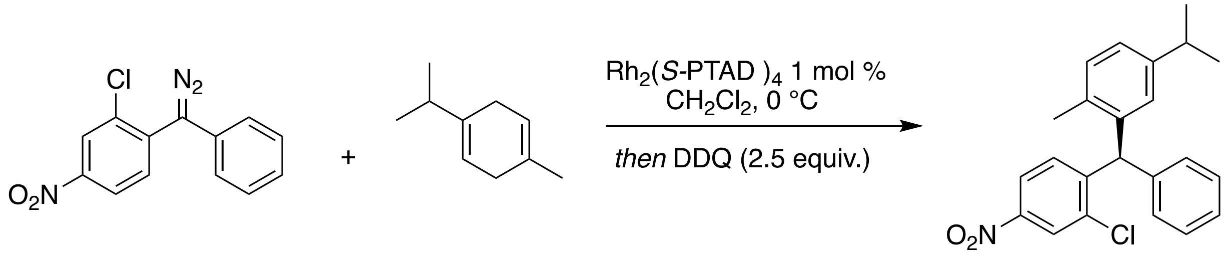

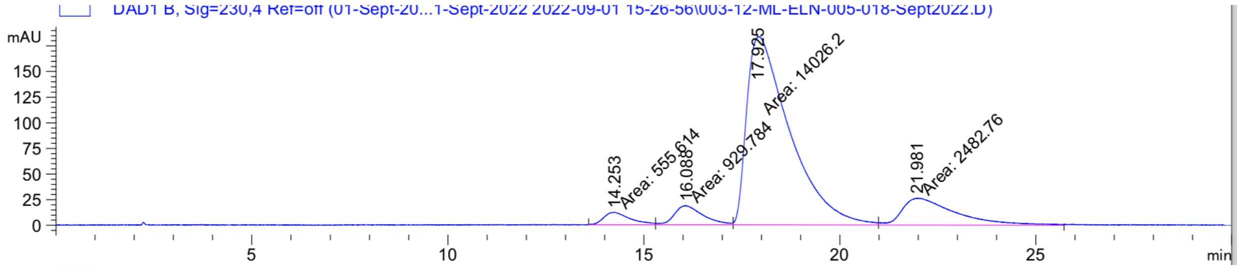

Signal 2: DAD1 B, Sig=230,4 Ref=off

Peak #	RetTime [min]	Type	Width [min]	Area [mAU*s]	Height [mAU]	Area %
1	14.253	MF	0.7573	555.61395	12.22836	3.0877
2	16.088	FM	0.8361	929.78375	18.53372	5.1671
3	17.925	MF	1.2735	1.40262e4	183.55954	77.9478
4	21.981	FM	1.5767	2482.75537	26.24434	13.7974

Totals : 1.79943e4 240.56597
